# Aryl acrylonitriles synthesis enabled by palladium-catalyzed α-alkenylation of arylacetonitriles with vinyl halides/triflates

**DOI:** 10.3389/fchem.2022.1091566

**Published:** 2022-12-15

**Authors:** Yonggang Jiang, Bijun Wang, Dongxiang Liu, Dazhen Xia, Zhengfen Liu, Liang Li, Guogang Deng, Xiaodong Yang

**Affiliations:** Key Laboratory of Medicinal Chemistry for Natural Resource, Ministry of Education, Yunnan Provincial Center for Research & Development of Natural Products, School of Pharmacy, Yunnan University, Kunming, China

**Keywords:** arylacetonitrile, palladium catalysis, alkenylation, isomerization, aryl acrylonitrile

## Abstract

Aryl acrylonitriles are an important subclass of acrylonitriles in the medicinal chemistry and pharmaceutical industry. Herein, an efficient synthesis of aryl acrylonitrile derivatives using a Palladium/NIXANTPHOS-based catalyst system was developed. This approach furnishes a variety of substituted and functionalized aryl acrylonitriles (up to 95% yield). The scalability of the transformation and the synthetic versatility of aryl acrylonitrile were demonstrated.

## Introduction

Acrylonitriles, especially substituted acrylonitriles, are versatile building blocks widely occurring in the pharmaceutical industry, natural products and synthetic organic chemistry (Fringuelli et al., 1994; Fleming, 1999; Fleming et al., 2010; Carta et al., 2011; Shen et al., 2015; Baker et al., 2020; Sirim et al., 2020; Solangi et al., 2020; Baker et al., 2021). Among acrylonitrile-containing molecules, aryl acrylonitriles are an important subclass in the medicinal chemistry and pharmaceutical industry (ANI-7 (Tarleton et al., 2011), CDCPA (Baker et al., 2018), TPAT-AN-XF (Niu et al., 2019), CC-5079 (Zhang et al., 2006), Entacapone (Seeberger and Hauser, 2009), and Rilpivirine (Clercq, 2005) Figure 1). Therefore, the development of efficient and practical approaches for the synthesis of aryl acrylonitriles remains in demand.

**FIGURE 1:**
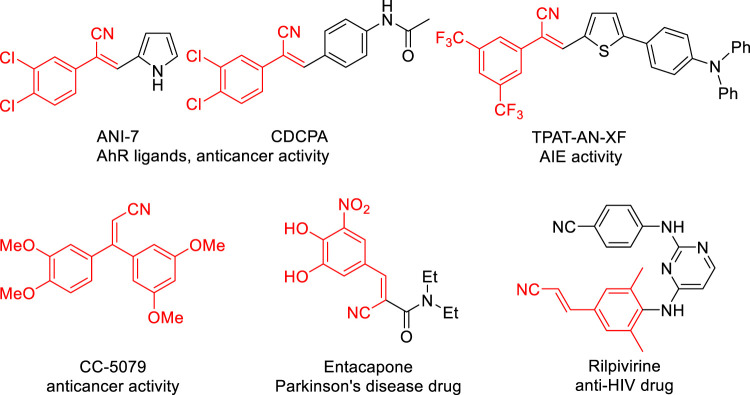
Representative examples of bioactive compounds with an aryl acrylonitrile.

**SCHEME 1:**
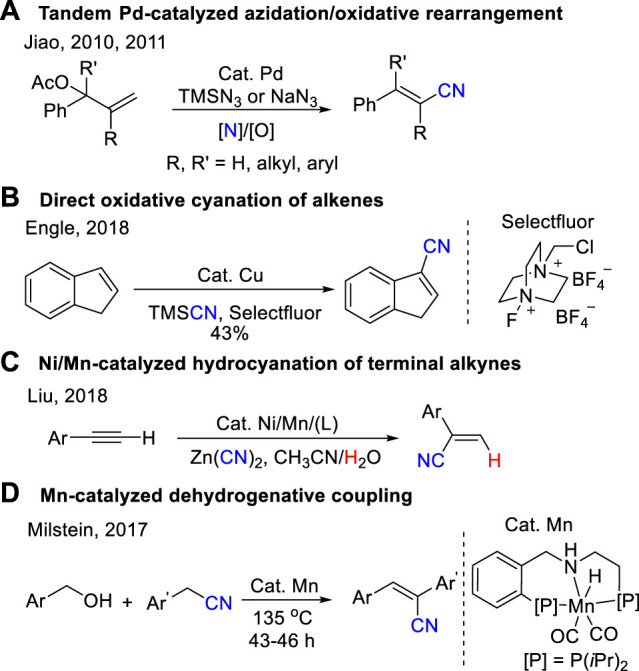
General strategies of aryl acrylonitriles.

Classical synthetic routes to acrylonitrile derivatives include the Wittig/Horner−Wadsworth−Emmons reaction (Zhang et al., 1998; Kojima et al., 2002; Fang et al., 2011; Ando et al., 2013) and Peterson type reactions (Kojima et al., 2004; Pabmo’ et al., 1990; Palomo et al., 1990). However, these procedures suffer from limitations such as a poor substrate scope, low efficiency for the synthesis of polysubstituted acrylonitriles. During the past decade, organic chemists keep searching new and efficient reactions, including oxidative Heck-type reactions (Zou et al., 2003; Zhang and Liebeskind, 2006), cyanation of alkenyl halides (Stuhl, 1985; Alterman and Hallberg, 2000; Pradal and Evano, 2014; Ahuja and Sudalai, 2015; Chaitanya and Anbarasan, 2015; Yang et al., 2018), alcohols (Oishi et al., 2009; Rokade et al., 2012; Thiyagarajan and Gunanathan, 2018; Yadav et al., 2020), aldehydes (Tomioka et al., 2011; Laulhe et al., 2012; Del Fiandra et al., 2016; Wu et al., 2016), acrylamide/oxime dehydration (Yamaguchi et al., 2007; Zhou et al., 2009), carbocyanation of alkynes (Nakao et al., 2007; Cheng et al., 2008; Minami et al., 2013; Yang et al., 2013; He et al., 2016; Qi et al., 2017), cross-metathesis (Crowe and Goldberg, 1995; Randl et al., 2001; Mu et al., 2019), and direct conversion of allylic carbon to nitrile (Qin and Jiao, 2010; Zhou et al., 2010) have been developed and could be applied for the synthesis of acrylonitriles. For example, Jiao developed a series of powerful synthesis of substituted acrylonitriles, which used allyl esters or halides and NaN_3_ or TMSN_3_ by a tandem Pd-catalyzed azidation and the subsequent oxidative rearrangement process (Scheme 1A) (Qin and Jiao, 2010; Zhou et al., 2010; Jiao et al., 2011; Wang and Jiao, 2014). Engle reported a direct oxidative cyanation of terminal and internal alkenes to prepare substituted acrylonitriles using a homogeneous copper catalyst and a bystanding N–F oxidant (Scheme 1B) (Gao et al., 2018). Recently, Liu reported an elegant synthesis of aryl substituted terminal acrylonitriles through Ni/Mn-catalyzed hydrocyanation of terminal alkynes with Zn(CN)_2_ (Scheme 1C) (Zhang et al., 2018). Milstein reported an effective synthesis of aryl acrylonitriles through dehydrogenative coupling of alcohols with nitriles catalyzed by a pincer complex of manganese at 135°C for 43–60 h (Scheme 1D) (Chakraborty et al., 2017).

Despite these advances, these motheds are generally restricted by the addition of dangerous reagents (cyanide reagents, azide reagents) and stoichiometric amount of oxidants (DDQ, Selectfluor), high catalyst loading, tedious synthetic procedures, low yielding and high reaction temperatures. Therefore, an optional method for the efficient synthesis of aryl acrylonitrile derivatives under mild reaction conditions using simple, easily available substrates are very necessary. Herein, we report an efficient synthesis of aryl acrylonitrile derivatives using a Palladium/NIXANTPHOS-based catalyst system. This approach furnishes efficient access to a variety of substituted and functionalized aryl acrylonitriles (21 examples, up to 95%). The scalability of the transformation was demonstrated and the derivatizations of the aryl acrylonitrile were conducted.

## Results and discussion

We initiated our reaction optimization by using phenylacetonitrile **1a** and 2-bromoprop-1-ene **2a** as the model substrates. At the outset, based on our experience with deprotonative cross-coupling processes of weakly acidic substrates (Yang et al., 2016; Duan et al., 2018; Liu et al., 2018), we have found that NIXANTPHOS can effectively implement these conversions. The high reactivity of the Pd/NIXANTPHOS-based system may be due to the presence of the main group metal and the deprotonation of the ligand N−H moiety under basic reaction conditions (Zhang et al., 2014). A variety of palladium source including different Pd^0^ and Pd^II^ precursors, phosphine ligands and six bases (LiN(SiMe_3_)_2_, NaN(SiMe_3_)_2_, KN(SiMe_3_)_2_, LiO^*t*^Bu, NaO^*t*^Bu and KO^*t*^Bu) were examined the coupling of phenylacetonitrile **1a** and 2-bromoprop-1-ene **2a** in DME at 65°C for 1 h (Table 1, entries 1–10) (see the optimization of reaction conditions on page S2 in Supplementary Material). The top Pd/L/base combination from this screen was Pd(OAc)_2_/NIXANTPHOS/NaO^*t*^Bu resulted in 20% assay yield (AY, determined by ^1^H NMR analysis). Other four solvents (dioxane, CPME, THF and Toluene) were tested, which only afforded trace amount of product (0%–13%) (entries 11–14). Raising the reaction temperature to 80 and 100°C led to increases to 57 and 28% AY, respectively (entries 15 and 16). Changing the equivalent of **2a** from 2 to 4 led to increases of AY (entries 17–19). When a 3 equivalent was employed, the AY increased to 77% (75% isolated yield, entry 18). Reducing the Pd/ligand ratio to 5:10, AY dropped to 57% (entry 20).

**TABLE 1:** Optimization of the reaction conditions^*a*^.

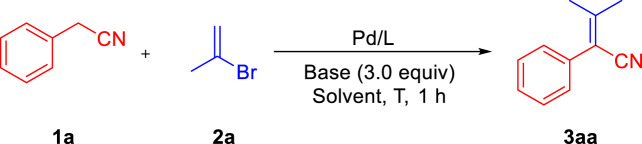								
Entry	Pd source	L	Base	Solvent	T (^o^C)	2a (equiv)	Pd/L (mol%)	AY (%)^*b*^
1	Pd(OAc)_2_	L1	B1	DME	65	1.5	10/20	10
2	PdCl_2_(cod)	L1	B1	DME	65	1.5	10/20	9
3	[PdCl(allyl)]_2_	L1	B1	DME	65	1.5	10/20	10
4	Pd(NCPh)_2_Cl_2_	L1	B1	DME	65	1.5	10/20	9
5	Pd (dba)_2_	L1	B1	DME	65	1.5	10/20	4
6	Pd_2_ (dba)_3_	L1	B1	DME	65	1.5	10/20	7
7	Pd(PPh_3_)_4_	L1	B1	DME	65	1.5	10/20	3
8	Pd(Cy_3_)_2_	L1	B1	DME	65	1.5	10/20	8
9	Pd(OAc)_2_	L2-L8	B1	DME	65	1.5	10/20	0–4
10	Pd(OAc)_2_	L1	B2-B6	DME	65	1.5	10/20	0–20
11	Pd(OAc)_2_	L1	B5	Dioxane	65	1.5	10/20	0
12	Pd(OAc)_2_	L1	B5	CPME	65	1.5	10/20	7
13	Pd(OAc)_2_	L1	B5	THF	65	1.5	10/20	3
14	Pd(OAc)_2_	L1	B5	Toluene	65	1.5	10/20	13
15	Pd(OAc)_2_	L1	B5	DME	80	1.5	10/20	57
16	Pd(OAc)_2_	L1	B5	DME	100	1.5	10/20	28
17	Pd(OAc)_2_	L1	B5	DME	80	2.0	10/20	73
18	Pd(OAc)_2_	L1	B5	DME	80	3.0	10/20	77 (75)^*c*^
19	Pd(OAc)_2_	L1	B5	DME	80	4.0	10/20	68
20	Pd(OAc)_2_	L1	B5	DME	80	3.0	5/10	57

^a^ Reactions conducted on a 0.1 mmol scale using **1a** and **2a**.

^b^ Assay yield determined by ^1^H NMR spectroscopy of the crude reaction mixture.

^c^ Isolated yield after chromatographic purification.

L1: NIXANTPHOS, L2: XANTPHOS, L3: PPh_3_, L4: P (*o*-TOL)_3_, L5: P (1-NAP)_3_, L6: *rac*-BINAP, L7: JOHNPHOS, L8: PCy_3_

B1: LiN(SiMe_3_)_2_, B2: NaN(SiMe_3_)_2_, B3: KN(SiMe_3_)_2_, B4: LiO^*t*^Bu, B5: NaO^*t*^Bu, B6: KO^*t*^Bu

With the optimized reaction conditions (Table 1, entry 18), we explored the structural diversity of vinyl halides/triflates using phenylacetonitrile **1a** as the model substrate. As shown in Table 2, 2-bromo-1-ene **2a** delivered aryl acrylonitrile **3aa** in 75% yield, while 2-chloro-1-ene **2a’** gave 55% yield. Vinyl chloride 1-chloro-2-methylprop-1-ene **2b** led to product **3ab** in 54% yield. (*E*)-(1-bromoprop-1-en-2-yl)benzene **2c** provided product **3ac** in 81% yield (67% yield for 5% Pd/10% L). Sterically hindered bromomethylenecyclohexane **2d** rendered product **3ad** with excellent yield of 84% yield (70% yield for 5% Pd/10% L). Trans- and cis-2-bromobut-2-ene (**2e** and **2f**) furnished products **3ae** and **3af** in overall 57% and 51% yields. 2-Bromo-3-methylbut-2-ene **2g** afforded product **3ag** in overall 65% yield. Cycloolefin halides/triflates were all suitable reaction partners in this transformation and provided a series of cycloalkane-functionalized aryl acrylonitriles in moderate yields. 1-Chlorocyclopent-1-ene **2h** led to product **3ah** in 50% yield. Six/seven/eight-membered cycloolefin triflates proceeded the corresponding products **3ai**, **3aj**, and **3ak** in 65%, 61% and 55% yields, respectively.

**TABLE 2:** Scope of vinyl halides/triflates^*a*^.

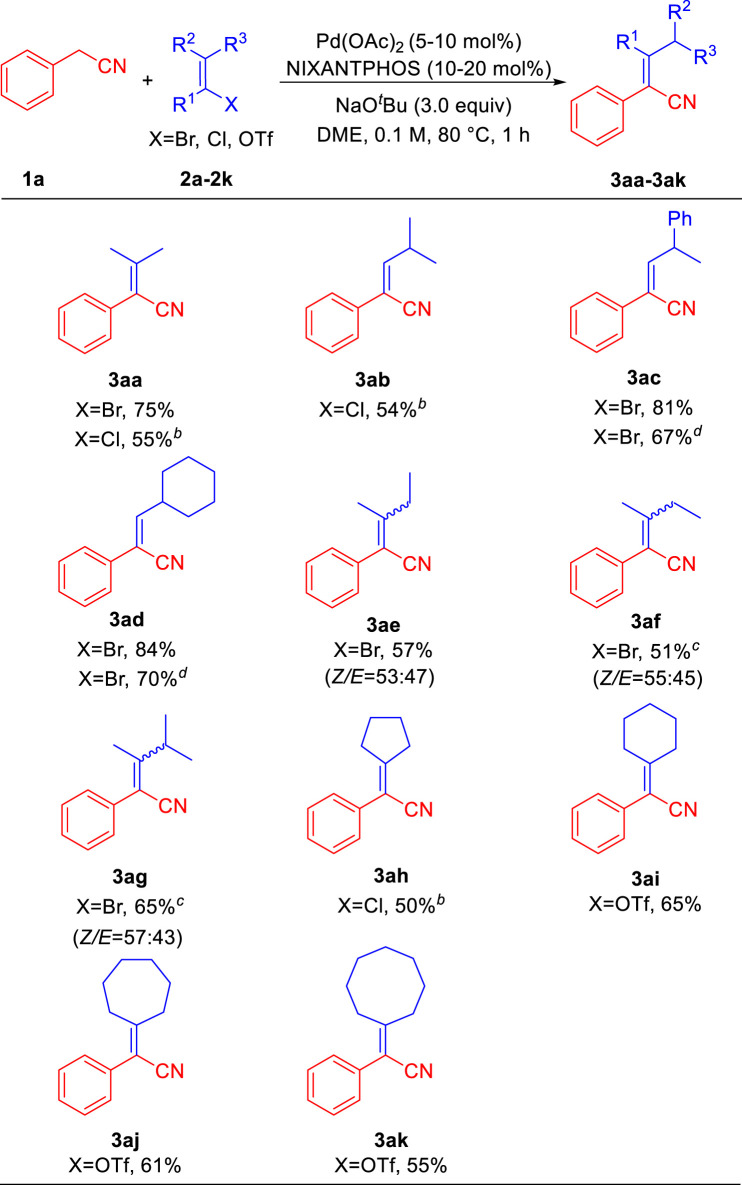

^a^ Reactions conducted on 0.3 mmol scale using 1.0 equiv of **1a** and 3.0 equiv of **2a**-**2k**. Isolated yield after chromatographic purification.

^b^ 7 h reaction time.

^c^ 100°C reaction temperature, 7 h reaction time.

^d^ 5 mol% Pd(OAc)_2_ and 10 mol% NIXANTPHOS, for the reaction.

We next explored the scope of arylacetonitriles using sterically hindered bromomethylenecyclohexane **2d** as the model substrate. As shown in Table 3, in general, arylacetonitriles bearing electron-donating and electron-withdrawing Ar groups or heterocyclic rendered good to excellent yields under the standard conditions (Table 3). Arylacetonitriles possessing alkyl 4-Me (**1b**) and 2-Me (**1c**) reacted with bromomethylenecyclohexane **2d** to give aryl acrylonitriles **3bd** and **3cd** in 84% and 81% yields (69% and 63% yields for 5% Pd). Arylacetonitrile with electro-donating (4-OMe, **1d**) substituents provided product **3dd** in 67% yield. Arylacetonitriles bearing electron-withdrawing 4-F (**1e**), 4-Cl (**1f**) and 4-Br (**1g**) generated the products **3ed**, **3fd** and **3gd** in 83% (78% yield for 5% Pd), 95% (79% yield for 5% Pd) and 50% yields, respectively. The sterically demanding 2-naphthyl acetonitrile (**1h**) was well tolerated, led to product **3hd** in 67% yield. Interesting, medicinally important heterocyclic-containing acetonitriles were suitable reaction partners. 2-(1-Methyl-1*H*-indol-3-yl)acetonitrile (**1i**) reacted with **2d** to generate the aryl acrylonitrile **3id** with excellent yield of 93% (79% yield for 5% Pd). 2-(Thiophen-2-yl)acetonitrile (**1j**) provided product **3jd** in 47% yield.

**TABLE 3:** Scope of arylacetonitriles^*a*^.

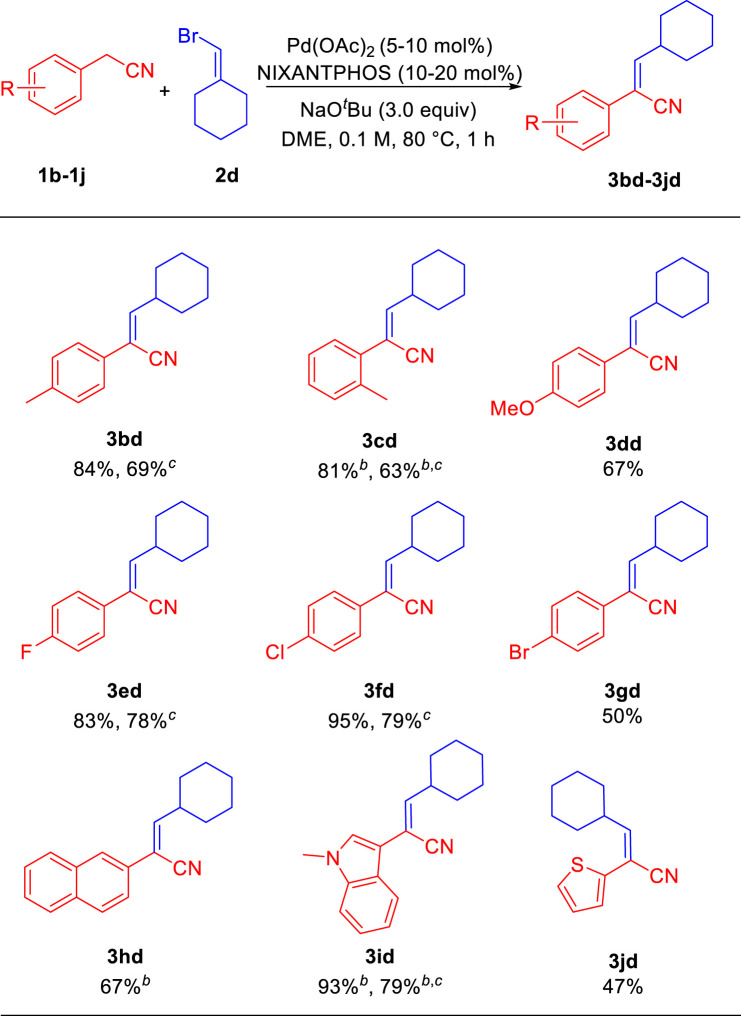

^a^ Reactions conducted on 0.3 mmol scale using 1.0 equiv of **1b**-**1j** and 3.0 equiv of **2d**. Isolated yield after chromatographic purification.

^b^ 7 h reaction time.

^c^ 5 mol% Pd(OAc)_2_ and 10 mol% NIXANTPHOS, for the reaction.

**SCHEME 2:**
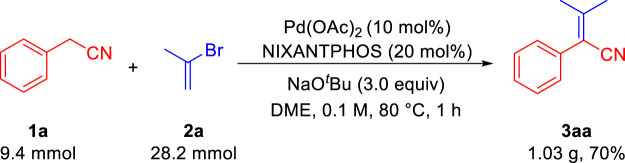
Synthesis of aryl acrylonitrile **3aa** in gram scale.

To evaluate the scalability of our transformation, we next carried out the reaction of phenylacetonitrile **1a** and 2-bromo-1-ene **2a** on a gram-scale under the optimal conditions (Scheme 2). The desired aryl acrylonitrile **3aa** was isolated in 1.03 g (70% yield), demonstrating the scalability of our method.

**SCHEME 3:**
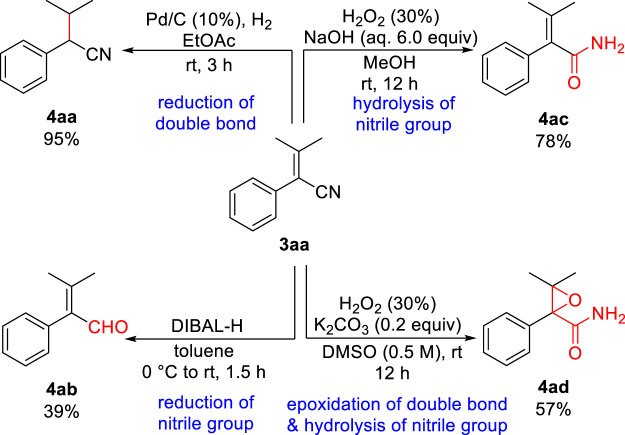
Derivatizations of aryl acrylonitrile **3aa**.

Finally, to illustrate further the synthetic versatility of the resulting aryl acrylonitrile, a series of derivatizations were performed on **3aa** (Scheme 3). Thus, the selective reduction of the carbon-carbon double bond of aryl acrylonitrile **3aa** using Pd/C and hydrogen led to the substituted saturated phenylacetonitrile **4aa** in 95% yield. Then, the selective reduction of the nitrile group of **3aa** employing DIBAL-H in toluene at 0°C generated the corresponding α,β-unsaturated aldehyde **4ab** in 39% yield (Chen et al., 2019). Meanwhile, the hydrolysis of the nitrile group of **3aa** using 30% H_2_O_2_ and NaOH in MeOH rendered the corresponding α,β-unsaturated amide **4ac** in 78% yield. Furthermore, the epoxidation of the carbon-carbon double bond and hydrolysis of the nitrile group of **3aa** using 30% H_2_O_2_ and K_2_CO_3_ in DMSO afforded the corresponding α,β-epoxy amide **4ad** in 57% yield.

**SCHEME 4:**
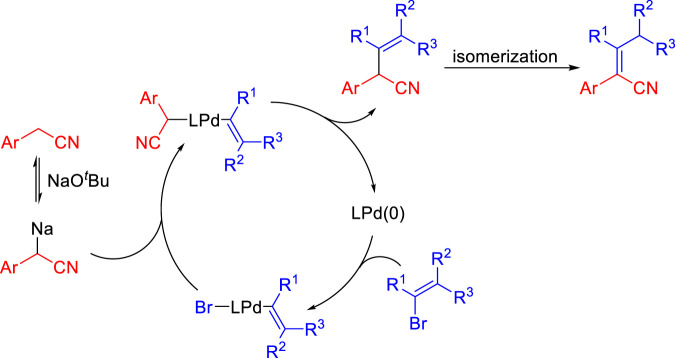
Plausible reaction mechanism.

A possible catalytic cycle is shown in Scheme 4 based on Walsh’s work on the palladium-catalyzed deprotonative cross-coupling processes (Hussain et al., 2014; Jia et al., 2014; Mao et al., 2014; Jia et al., 2015). The deprotonation of aryl acetonitrile by NaO^*t*^Bu gives benzyl anions. After oxidative addition of the vinyl bromide to Pd (0), the vinyl palladium intermediate is proposed to bind the benzyl anions to form the palladium complex. Then, reductive elimination occurs to afford the enenitrile and regenerates Pd (0). Finally, enenitrile isomerizes to obtain aryl acrylonitrile.

## Conclusion

In conclusion, we have successfully synthesized a series of aryl acrylonitrile derivatives employing a Pd/NIXANTPHOS-based catalyst system for the first time. In this protocol, commercially available arylacetonitriles and vinyl bromides/chlorides/triflates underwent palladium-catalyzed α-alkenylation to furnish efficient access to a variety of substituted and functionalized aryl acrylonitriles. The scalability of the mothed was demonstrated by the gram-scale reaction. A series of derivatization of aryl acrylonitrile were performed, including the selective reduction of the double bond or nitrile group, the hydrolysis of the nitrile group, and the epoxidation of the double bond, which demonstrated the synthetic versatility of aryl acrylonitrile. It is noteworthy that this approach does not require dangerous reagents and stoichiometric amount of oxidants, which enables the synthesis of a range of aryl acrylonitriles in an effective and straightforward means.

## Data Availability

The original contributions presented in the study are included in the article/Supplementary Material, further inquiries can be directed to the corresponding authors.
