# Supplementary material for: Aryl acrylonitriles synthesis enabled by palladium-catalyzed α-alkenylation of arylacetonitriles with vinyl halides/triflates
Source: Front Chem. 2022 Dec 15;10:1091566. doi: 10.3389/fchem.2022.1091566 (PMC9798101; doi:10.3389/fchem.2022.1091566)

Supplementary Material

Aryl Acrylonitriles Synthesis Enabled by Palladium-Catalyzed α-Alkenylation of Arylacetonitriles with Vinyl Halides/Triflates

Yonggang Jiang†, Bijun Wang†, Dongxiang Liu, Dazhen Xia, Zhengfen Liu, Liang Li, Guogang Deng*, Xiaodong Yang*

Key Laboratory of Medicinal Chemistry for Natural Resource, Ministry of Education; Yunnan Provincial Center for Research & Development of Natural Products; School of Pharmacy, Yunnan University, Kunming, P. R. China

*** Correspondence:**
Guogang Deng
ggdeng@ynu.edu.cn
Xiaodong Yang
xdyang@ynu.edu.cn

† These authors have contributed equally to this work.

TABLE OF CONTENT

General methods. S2

Procedure and characterization for the aryl acrylonitriles of arylacetonitriles with vinyl halides/triflates. S2

Gram scale synthesis of 3aa S11

Derivatization studies of product 3aa S11

NMR Spectra S15

General methods.

All air- and moisture-sensitive solutions and chemicals were handled under a nitrogen atmosphere of a glovebox and solutions were transferred via "Eppendorf" brand pipettor. Anhydrous solvents, including DME (dimethoxyethane), dioxane, CPME (cyclopentyl methyl ether) and tetrahydrofuran (THF) were purchased from Sigma-Aldrich and used without further purification. Toluene was dried through activated alumina columns. Unless otherwise stated, all reagents were commercially available and used as received without further purification. Chemicals were obtained from Sigma-Aldrich, Acros or Adamas-beta, TCI and Alfa-Aesar. TLC was performed with Merck TLC Silica gel60 F_{254} plates with detection under UV light at 254 nm. Silica gel (200-300 mesh, Qingdao) was used for flash chromatography. Proton nuclear magnetic resonance (^{1}H-NMR) spectra were recorded on a Bruker DRX 400 spectrometer at 400 MHz. Carbon-13 nuclear magnetic resonance (^{13}C-NMR) were recorded on Bruker DRX 400 spectrometer at 100 MHz. Chemical shifts were reported in units of parts per million (ppm) downfield from tetramethylsilane (TMS), and all coupling constants were reported in hertz. The infrared (IR) spectra were measured on a Nicolette Avatar FT-IR spectrometer with 4 cm^{-1} resolution and 32 scans between wavenumbers of 4000 cm^{-1} and 400 cm^{-1}. High Resolution Mass spectra were taken on Aglient LC-MSO/TOR mass spectrometer. Melting points were obtained on an XT-4 melting-point apparatus and were uncorrected.

Procedure and characterization for the aryl acrylonitriles of arylacetonitriles with vinyl halides/triflates.

General Procedure:

An oven-dried 8 mL reaction vial equipped with a stir bar was charged with $Pd(OAc)_2$ (6.8 mg, 10 mol%) or (3.4 mg, 5 mol%), NIXANTPHOS (33.0 mg, 20 mol%) or (16.5 mg, 10 mol%), NaO^{t}Bu (86.5 mg, 0.9 mmol), arylacetonitriles **1** (0.3 mmol) and vinyl halides/triflates **2** (0.9 mmol) under a nitrogen atmosphere in a glove box. Then a solvent of 3 mL dry DME was added to the reaction vial. The vial was sealed with a cap, removed from the glove box, and stirred for 1 – 7 h at 80 – 100 °C. The reaction mixture was cooled to room temperature and then opened to air, quenched with three drops of H_2O, diluted with 3 mL of ethyl acetate, and filtered over a 2 cm pad of silica. The pad was rinsed with ethyl acetate (3 X 3 mL), and the combined organic solutions were concentrated in vacuo. The crude product was chromatographed on silica gel to give the product **3**.

Optimization of the reaction conditions:

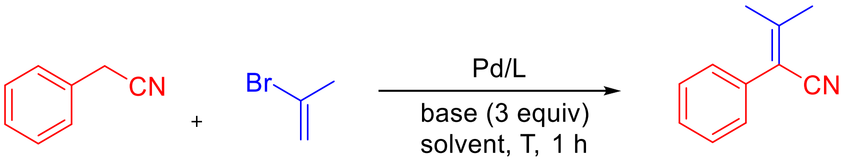

1a **2a** **3aa**

<table>
<tr><th>Entry</th><th>Pd source</th><th>L</th><th>Pd/L (mol %)</th><th>Base</th><th>Solvent</th><th>Temp. (°C)</th><th>Assay Yield (%)</th></tr>
<tr><td>1</td><td>$Pd(OAc)_2$</td><td rowspan="4">L1</td><td rowspan="4">10/20</td><td rowspan="4">$LiN(SiMe_3)_2$</td><td rowspan="4">DME</td><td rowspan="4">65</td><td>10</td></tr>
<tr><td>2</td><td>$PdCl_2(cod)$</td><td>9</td></tr>
<tr><td>3</td><td>$[PdCl(allyl)]_2$</td><td>10</td></tr>
<tr><td>4</td><td>$Pd(NCPh)_2Cl_2$</td><td>9</td></tr>
</table>

5	$Pd(dba)_2$						4
6	$Pd_2(dba)_3$						7
7	$Pd(PPh_3)_4$						3
8	$Pd(Cy_3)_2$						8
9		L2					4
10		L3					0
11		L4					0
12	$Pd(OAc)_2$	L5	10/20	$LiN(SiMe_3)_2$	DME	65	0
13		L6					0
14		L7					0
15		L8					0
16				$NaN(SiMe_3)_2$			0
17				$KN(SiMe_3)_2$			0
18	$Pd(OAc)_2$	L1	10/20	LiO^tBu	DME	65	16
19				NaO^tBu			20
20				KO^tBu			0
21					CPME		7
22					THF		3
23	$Pd(OAc)_2$	L1	10/20	NaO^tBu	Toluene	65	13
24					Dioxane		0
25	$Pd(OAc)_2$	L1	10/20	NaO^tBu	DME	45	3
26						80	57
27						100	28
28[c]	$Pd(OAc)_2$	L1	10/20	NaO^tBu	DME	80	73
29[d]							77
30[d]	$Pd(OAc)_2$	L1	2.5/5	NaO^tBu	DME	80	14
31[d]			5/10				57

[a] Reactions conducted on a 0.1 mmol scale using 1 equiv of **1a**, and 1.5 equiv of **2a**.

[b] Assay yield determined using CH_2Br_2 as the internal standard.

[c] Reactions conducted on a 0.1 mmol scale using 1 equiv of **1a**, and 2 equiv of **2a**.

[d] Reactions conducted on a 0.1 mmol scale using 1 equiv of **1a**, and 3 equiv of **2a**.

Ligands

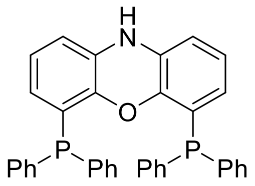

NIXANTPHOS (**L1**)

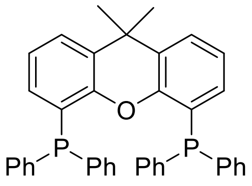

XANTPHOS (**L2**)

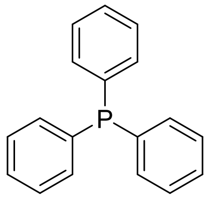

PPh_3 (**L3**)

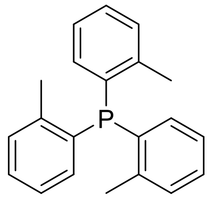

P(*o*-TOL)$_3$ (**L4**)

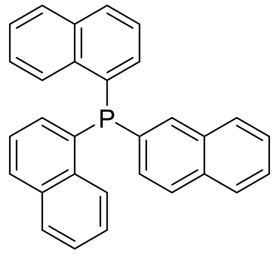

P(1-NAP)$_3$ (**L5**)

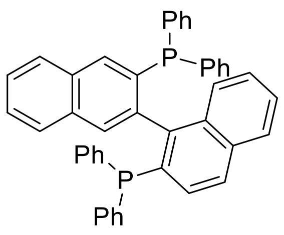

rac-BINAP (**L6**)

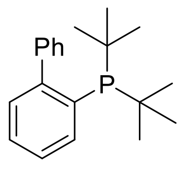

JOHNPHOS (**L7**)

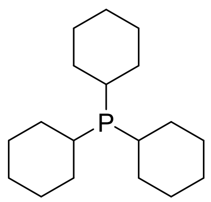

PCy_3 (**L8**)

3-methyl-2-phenylbut-2-enenitrile (3aa)

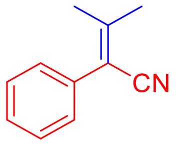

The reaction was performed following the General Procedure with 2-phenylacetonitrile **1a** (35.1 mg, 0.3 mmol) and 2-bromoprop-1-ene **2a** (108.9 mg, 0.9 mmol) or 2-chloroprop-1-ene **2a'** (68.9 mg, 0.9 mmol). The crude product was chromatographed on silica gel to give the product **3aa** (35.4 mg, 75% yield, from **2a**) or (25.9 mg, 55% yield, from **2a'**) as a colorless oil. R_f = 0.37 (petroleum ether:ethyl acetate = 20:1). ^{1}H NMR (400 MHz, Chloroform-*d*) δ 7.32 – 7.18 (m, 5H), 2.16 (s, 3H), 1.82 (s, 3H) ppm. ^{13}C{^{1}H} NMR (100 MHz, Chloroform-*d*) δ 154.7, 134.1, 129.1, 128.6, 128.2, 118.9, 111.0, 25.0, 21.7 ppm. IR (thin film): 3026, 2915, 2855, 2210, 1624, 1599, 1492, 1444, 1374, 1214, 1073, 996, 764, 701, 682 cm^{-1}. HRMS calc'd for $C_{11}H_{11}NNa^+$ 180.0784, found 180.0789 [M+Na]$^+$.

(*Z*)-4-methyl-2-phenylpent-2-enenitrile (3ab)

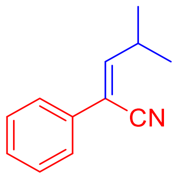

The reaction was performed following the General Procedure with 2-phenylacetonitrile **1a** (35.1 mg, 0.3 mmol) and 1-chloro-2-methylprop-1-ene **2b** (81.5 mg, 0.9 mmol). The crude product was chromatographed on silica gel to give the product **3ab** (27.7 mg, 54% yield) as a colorless oil. R_f = 0.63 (petroleum ether:ethyl acetate = 20:1). ^{1}H NMR (400 MHz, Chloroform-*d*) δ 7.47 – 7.44 (m, 2H), 7.35 – 7.27 (m, 3H), 6.58 (d, *J* = 10.0 Hz, 1H), 3.10 – 3.04 (m, 1H), 1.11 (d, *J* = 6.4 Hz, 6H) ppm. ^{13}C{^{1}H} NMR (100 MHz, Chloroform-*d*) δ 153.6, 133.3, 129.03, 128.96, 125.8, 116.7, 113.7, 32.1, 22.3 ppm. IR (thin film): 3063, 2966, 2930, 2872, 2218, 1616, 1493, 1465, 1448, 1386, 1253, 1097, 1078, 941, 764, 693 cm^{-1}. HRMS calc'd for $C_{12}H_{14}N^+$ 172.1121, found 172.1121 [M+H]$^+$.

(*Z*)-2,4-diphenylpent-2-enenitrile (3ac)

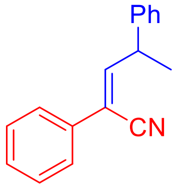

The reaction was performed following the General Procedure with 2-phenylacetonitrile **1a** (35.1 mg, 0.3 mmol) and (*E*)-(1-bromoprop-1-en-2-yl)benzene **2c** (177.4 mg, 0.9 mmol). The crude product was chromatographed on silica gel to give the product **3ac** (56.7 mg, 81% yield for 10% Pd/20% L; 46.9 mg, 67% yield for 5% Pd/10% L) as a colorless oil. R_f = 0.46 (petroleum ether:ethyl acetate = 20:1). ^{1}H NMR (400 MHz, Chloroform-*d*) δ 7.55 – 7.50 (m, 2H), 7.37 – 7.33 (m, 7H), 7.25 (td, *J* = 6.0, 2.8 Hz, 1H), 6.84 (d, *J* = 10.0 Hz, 1H), 4.27 – 4.19 (m, 1H), 1.54 (d, *J* = 6.8 Hz, 3H) ppm. ^{13}C{^{1}H} NMR (100 MHz, Chloroform-*d*) δ 150.4, 142.8, 133.0, 129.1, 129.02, 128.99, 127.2, 127.0, 125.9, 116.7, 114.2, 42.4, 20.7 ppm. IR (thin film): 3062, 3028, 2970, 2929, 2219, 1600, 1494, 1449, 1375, 1231, 1184, 1019, 909, 726, 668 cm^{-1}. HRMS calc'd for $C_{17}H_{16}N^+$ 234.1277, found 234.1277 [M+H]$^+$.

(*Z*)-3-cyclohexyl-2-phenylacrylonitrile (3ad)

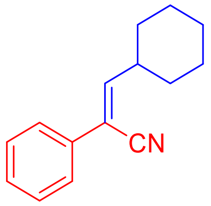

The reaction was performed following the General Procedure with 2-phenylacetonitrile **1a** (35.1 mg, 0.3 mmol) and (bromomethylene)cyclohexane **2d** (157.6 mg, 0.9 mmol). The crude product was chromatographed on silica gel to give the product **3ad** (53.3 mg, 84% yield for 10% Pd/20% L; 44.4 mg, 70% yield for 5% Pd/10% L) as a colorless oil. R_f = 0.53 (petroleum ether:ethyl acetate = 20:1). ^{1}H NMR (400 MHz, Chloroform-*d*) δ 7.45 – 7.43 (m, 2H), 7.31 – 7.24 (m, 3H), 6.57 (d, *J* = 10.0 Hz, 1H), 2.71 – 2.63 (m, 1H), 1.75 – 1.61 (m, 5H), 1.35 – 1.18 (m, 2H), 1.17 – 1.10 (m, 3H) ppm. ^{13}C{^{1}H} NMR (100 MHz, Chloroform-*d*) δ 152.1, 133.3, 128.9, 128.8, 125.7, 116.8, 113.8, 41.5, 32.2, 25.7, 25.3 ppm. IR (thin film): 3062, 3028, 2928, 2853, 2217, 1498, 1449, 1342, 1259, 1078, 989, 952, 904, 764, 692 cm^1. HRMS calc'd for $C_{15}H_{18}N^+$ 212.1434, found 212.1435 [M+H]$^+$.

(3-methyl-2-phenyl-1λ^3-pent-2-en-1-ylidene)-λ^2-azane (3ae)

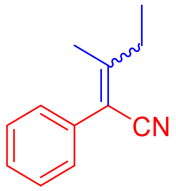

The reaction was performed following the General Procedure with 2-phenylacetonitrile **1a** (35.1 mg, 0.3 mmol) and (*E*)-2-bromobut-2-ene **2e** (121.5 mg, 0.9 mmol). The crude product was chromatographed on silica gel to give the product **3ae** (29.3 mg, 57% yield) as a yellow oil. *Z/E*=53:47. R_f = 0.43 (petroleum ether:ethyl acetate = 20:1). ^{1}H NMR (400 MHz, Chloroform-*d*) δ 7.33 – 7.19 (m, 5H, *E*+*Z*), 2.53 (q, *J* = 7.6 Hz, 0.81H, *Z*), 2.18 – 2.12 (m, 1.84H, *E*+*Z*), 1.83 (s, 1.24H, *E*), 1.14 (t, *J* = 7.6 Hz, 1.22H, *Z*), 0.99 (t, *J* = 7.6 Hz, 1.09H, *E*) ppm. ^{13}C{^{1}H} NMR (100 MHz, Chloroform-*d*) δ 160.2, 160.0, 134.2, 129.27, 129.26, 129.07, 129.05, 128.83, 128.81, 128.7, 128.4, 128.3, 119.0, 118.7, 110.9, 110.4, 31.9, 27.7, 21.9, 19.2, 12.7, 12.6 ppm. IR (thin film): 3059, 3026, 2974, 2937, 2877, 2210, 1618, 1493, 1445, 1377, 1263, 1073, 1019, 790, 701 cm^{-1}; HRMS calc'd for $C_{12}H_{14}N^+$ 172.1121, found 172.1119 [M+H]$^+$.

3-methyl-2-phenylpent-2-enenitrile (3af)

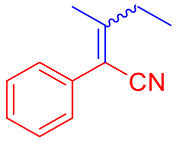

The reaction was performed following the General Procedure with 2-phenylacetonitrile **1a** (35.1 mg, 0.3 mmol) and (*Z*)-2-bromobut-2-ene **2f** (121.5 mg, 0.9 mmol). The crude product was chromatographed on silica gel to give the product **3af** (26.2 mg, 51% yield) as a colorless oil. *Z/E*=55:45. R_f = 0.43 (petroleum ether:ethyl acetate = 20:1). ^{1}H NMR (400 MHz, Chloroform-*d*) δ 7.29 – 7.14 (m, 5H, *E*+*Z*), 2.48 (q, *J* = 7.6 Hz, 1.06H, *Z*), 2.11 – 2.09 (m, 2.14H, *E*+*Z*), 1.78 (s, 1.56H, *E*), 1.09 (t, *J* = 7.6 Hz, 1.59H, *Z*), 0.94

(t, J = 7.6 Hz, 1.31H, E) ppm. $^{13}C\{^1H\}$ NMR (100 MHz, Chloroform-d) δ 160.2, 160.0, 134.3, 134.2, 129.3, 129.1, 128.8, 128.7, 128.4, 128.3, 119.0, 118.8, 110.9, 110.4, 32.0, 27.7, 21.9, 19.2 12.7, 12.6 ppm. IR (thin film): 3058, 3026, 2974, 2936, 2877, 2210, 1618, 1492, 1445, 1377, 1073, 764, 701 cm^{-1}; HRMS calc'd for $C_{12}H_{14}N^+$ 172.1121, found 172.1120 $[M+H]^+$.

3,4-dimethyl-2-phenylpent-2-enenitrile (3ag)

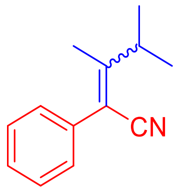

The reaction was performed following the General Procedure with 2-phenylacetonitrile **1a** (35.1 mg, 0.3 mmol) and 2-bromo-3-methylbut-2-ene **2g** (134.1 mg, 0.9 mmol). The crude product was chromatographed on silica gel to give the product **3ag** (36.1 mg, 65% yield) as a yellow oil. Z/E=57:43. R_f = 0.63 (petroleum ether:ethyl acetate = 20:1). ^1H NMR (400 MHz, Chloroform-d) δ 7.29 – 7.12 (m, 5H, E+Z), 3.24 (ddt, J = 13.6, 10.0, 5.2 Hz, 0.49H, Z), 2.75 (ddt, J = 13.6, 10.0, 5.2 Hz, 0.35H, E), 2.01 (s, 1.16H, E), 1.66 (t, J = 2.0 Hz, 1.67H, Z), 1.05 (dd, J = 7.2, 3.2 Hz, 3.40H, Z), 0.89 – 0.87 (m, 2.68H, E) ppm. $^{13}C\{^1H\}$ NMR (100 MHz, Chloroform-d) δ 163.8, 163.6, 134.4, 134.2, 129.4, 129.1, 128.9, 128.72, 128.67, 128.4, 128.3, 119.0, 118.6, 110.2, 109.5, 35.9, 31.0, 20.7, 20.6, 16.8, 14.3 ppm. IR (thin film): 3060, 2969, 2932, 2873, 2209, 1610, 1493, 1465, 1445, 1378, 1365, 1076, 762, 726, 700 cm^{-1}; HRMS calc'd for $C_{13}H_{15}NNa^+$ 208.1097, found 208.1097 $[M+Na]^+$.

2-cyclopentylidene-2-phenylacetonitrile (3ah)

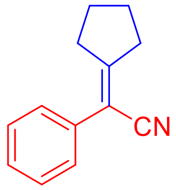

The reaction was performed following the General Procedure with 2-phenylacetonitrile **1a** (35.1 mg, 0.3 mmol) and 1-chlorocyclopent-1-ene **2h** (92.3 mg, 0.9 mmol). The crude product was chromatographed on silica gel to give the product **3ah** (27.5 mg, 50% yield) as a colorless oil. R_f = 0.37 (petroleum ether:ethyl acetate = 20:1). ^1H NMR (400 MHz, Chloroform-d) δ 7.36 – 7.30 (m, 4H), 7.26 – 7.20 (m, 1H), 2.76 – 2.73 (m, 2H), 2.55 – 2.51 (m, 2H), 1.79 – 1.69 (m, 4H) ppm. $^{13}C\{^1H\}$ NMR (100 MHz, Chloroform-d) δ 167.1, 134.4, 128.7, 128.1, 128.0, 118.8, 107.2, 36.0, 34.0, 27.1, 25.6 ppm. IR (thin film): 3057, 3027, 2926, 2873, 2212, 1621, 1493, 1446, 1420, 1262, 1018, 763, 668 cm^{-1}. HRMS calc'd for $C_{13}H_{13}NNa^+$ 206.0940, found 206.0937 $[M+Na]^+$.

2-cyclohexylidene-2-phenylacetonitrile (3ai)

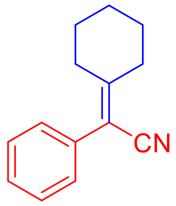

The reaction was performed following the General Procedure with 2-phenylacetonitrile **1a** (35.1 mg, 0.3 mmol) and cyclohex-1-en-1-yl trifluoromethanesulfonate **2i** (207.2 mg, 0.9 mmol). The crude product was chromatographed on silica gel to give the product **3ai** (38.5 mg, 65% yield) as a colorless oil. R_f = 0.47 (petroleum ether:ethyl acetate = 20:1). ^{1}H NMR (400 MHz, Chloroform-*d*) δ 7.31 – 7.18 (m, 5H), 2.61 – 2.58 (m, 2H), 2.23 – 2.21 (m, 2H), 1.71 – 1.66 (m, 2H), 1.58 – 1.46 (m, 4H) ppm. ^{13}C{^{1}H} NMR (100 MHz, Chloroform-*d*) δ 161.9, 133.9, 129.3, 128.7, 128.2, 118.7, 107.7, 35.4, 31.3, 28.1, 28.0, 25.9 ppm. IR (thin film): 3058, 2934, 2857, 2208, 1617, 1492, 1445, 1319, 1264, 1235, 1075, 1020, 1002, 982, 763, 701 cm^{-1}. HRMS calc'd for $C_{14}H_{16}N^+$ 198.1277, found 198.1276 $[M+H]^+$.

2-cycloheptylidene-2-phenylacetonitrile (3aj)

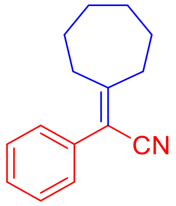

The reaction was performed following the General Procedure with 2-phenylacetonitrile **1a** (35.1 mg, 0.3 mmol) and cyclohept-1-en-1-yl trifluoromethanesulfonate **2j** (219.8 mg, 0.9 mmol). The crude product was chromatographed on silica gel to give the product **3aj** (38.7 mg, 61% yield) as a colorless oil. R_f = 0.40 (petroleum ether:ethyl acetate = 20:1). ^{1}H NMR (400 MHz, Chloroform-*d*) δ 7.31 – 7.18 (m, 5H), 2.75 – 2.66 (m, 2H), 2.33 (t, *J* = 12.0 Hz, 2H), 1.75 – 1.68 (m, 2H), 1.56 – 1.50 (m, 4H), 1.45 – 1.41 (m, 2H) ppm. ^{13}C{^{1}H} NMR (100 MHz, Chloroform-*d*) δ 164.5, 134.3, 129.1, 128.6, 128.2, 118.8, 110.4, 36.1, 32.8, 29.6, 28.7, 27.3, 26.9 ppm. IR (thin film): 3057, 2926, 2854, 2207, 1604, 1491, 1444, 1352, 1272, 1076, 1021, 957, 845, 767, 700 cm^{-1}. HRMS calc'd for $C_{15}H_{18}N^+$ 212.1434, found 212.1434 $[M+H]^+$.

2-cyclooctylidene-2-phenylacetonitrile (3ak)

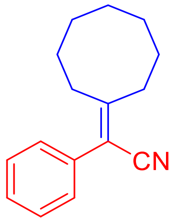

The reaction was performed following the General Procedure with 2-phenylacetonitrile **1a** (35.1 mg, 0.3 mmol) and (*E*)-cyclooct-1-en-1-yl trifluoromethanesulfonate **2k** (232.4 mg, 0.9 mmol). The crude product was chromatographed on silica gel to give the product **3ak** (37.2 mg, 55% yield) as a colorless oil. R_f = 0.50 (petroleum ether:ethyl acetate = 20:1). ^{1}H NMR (400 MHz, Chloroform-*d*) δ 7.41 – 7.27 (m, 5H), 2.72 (t, *J* = 16.0 Hz, 2H), 2.36 (dd, *J* = 7.2, 4.8 Hz, 2H), 1.98 – 1.92 (m, 2H), 1.63 (dt, *J* = 29.2, 5.6 Hz, 4H), 1.47 (dt, *J* = 6.0, 2.8 Hz, 4H) ppm. ^{13}C{^{1}H} NMR (100 MHz, Chloroform-*d*) δ 166.3, 134.6, 129.1, 128.8, 128.2, 119.0, 109.9, 34.4, 32.8, 28.5, 27.7, 25.9, 25.6, 23.8 ppm. IR (thin film): 3057, 2928, 2859, 2205, 1605, 1492, 1471, 1445, 1359, 1073, 1017, 992, 761, 701 cm^{-1}. HRMS calc'd for $C_{16}H_{20}N^+$ 226.1590, found 226.1590 $[M+H]^+$.

(*Z*)-3-cyclohexyl-2-(p-tolyl)acrylonitrile (3bd)

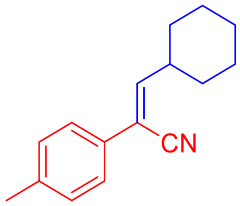

The reaction was performed following the General Procedure with 2-(*p*-tolyl)acetonitrile **1b** (39.4 mg, 0.3 mmol) and (bromomethylene)cyclohexane **2d** (157.6 mg, 0.9 mmol). The crude product was chromatographed on silica gel to give the product **3bd** (56.8 mg, 84% yield for 10% Pd/20% L; 46.6 mg, 69% yield for 5% Pd/10% L) as a colorless oil. R_f = 0.57 (petroleum ether:ethyl acetate = 20:1). ^{1}H NMR (400 MHz, Chloroform-*d*) δ 7.41 (d, *J* = 8.0 Hz, 2H), 7.19 (d, *J* = 8.0 Hz, 2H), 6.60 (d, *J* = 10.0 Hz, 1H), 2.78 – 2.70 (m, 1H), 2.36 (s, 3H), 1.83 – 1.70 (m, 5H), 1.46 – 1.34 (m, 2H), 1.28 – 1.18 (m, 3H) ppm. ^{13}C{^{1}H} NMR (100 MHz, Chloroform-*d*) δ 151.2, 139.0, 130.7, 129.7, 125.6, 117.0, 113.7, 41.4, 32.3, 25.8, 25.4, 21.3 ppm. IR (thin film): 3028, 2927, 2853, 2218, 1614, 1513, 1449, 1350, 1301, 1260, 1191, 1127, 953, 904, 817 cm^{-1}. HRMS calc'd for $C_{16}H_{19}NNa^+$ 248.1410, found 248.1408 $[M+Na]^+$.

(*Z*)-3-cyclohexyl-2-(*o*-tolyl)acrylonitrile (3cd)

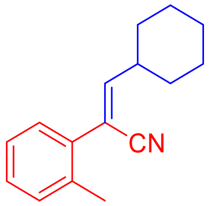

The reaction was performed following the General Procedure with 2-(*o*-tolyl)acetonitrile **1c** (39.4 mg, 0.3 mmol) and (bromomethylene)cyclohexane **2d** (157.6 mg, 0.9 mmol). The crude product was chromatographed on silica gel to give the product **3cd** (54.8 mg, 81% yield for 10% Pd/20% L; 42.6 mg, 63% yield for 5% Pd/10% L) as a colorless oil. R_f = 0.53 (petroleum ether:ethyl acetate = 20:1). ^{1}H NMR (400 MHz, Chloroform-*d*) δ 7.26 – 7.17 (m, 4H), 6.27 (d, *J* = 10.0 Hz, 1H), 2.81 – 2.71 (m, 1H), 2.40 (s, 3H), 1.86 – 1.69 (m, 5H), 1.46 – 1.35 (m, 2H), 1.24 (m, 3H) ppm. ^{13}C{^{1}H} NMR (100 MHz, Chloroform-*d*) δ 157.4, 136.0, 134.3, 130.8, 129.4, 129.0, 126.4, 116.9, 113.2, 41.2, 32.2, 25.7, 25.3, 20.1 ppm. IR (thin film): 3020, 2928, 2853, 2214, 1486, 1449, 1383, 988, 953, 908, 765, 736 cm^{-1}. HRMS calc'd for $C_{16}H_{19}NNa^+$ 248.1410, found 248.1411 $[M+Na]^+$.

(*Z*)-3-cyclohexyl-2-(4-methoxyphenyl)acrylonitrile (3dd)

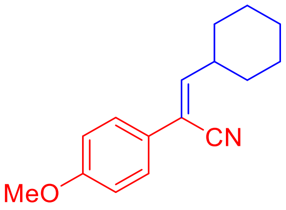

The reaction was performed following the General Procedure with 2-(4-methoxyphenyl)acetonitrile **1d** (44.2 mg, 0.3 mmol) and (bromomethylene)cyclohexane **2d** (157.6 mg, 0.9 mmol). The crude product was chromatographed on silica gel to give the product **3dd** (48.5 mg, 67% yield) as a colorless oil. R_f = 0.33 (petroleum ether:ethyl acetate = 20:1). ^{1}H NMR (400 MHz, Chloroform-*d*) δ 7.46 – 6.91 (m, 2H), 6.90 – 6.89 (m, 2H), 6.52 (d, *J* = 10.0 Hz, 1H), 3.82 (s, 3H), 2.77 – 2.67 (m, 1H), 1.83 – 1.69 (m, 5H),

1.41 – 1.34 (m, 2H), 1.27 – 1.16 (m, 3H) ppm. $^{13}C\{^1H\}$ NMR (100 MHz, Chloroform-*d*) δ 160.2, 150.1, 127.0, 126.0, 117.1, 114.3, 113.2, 55.5, 41.4, 32.4, 25.8, 25.4 ppm. IR (thin film): 2928, 2852, 2217, 1668, 1576, 1514, 1464, 1449, 1290, 1252, 1182, 1104, 953, 831 cm^{-1}. HRMS calc'd for $C_{16}H_{19}NONa^+$ 264.1359, found 264.1360 [M+Na]$^+$

(*Z*)-3-cyclohexyl-2-(4-fluorophenyl)acrylonitrile (3ed)

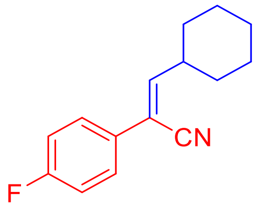

The reaction was performed following the General Procedure with 2-(4-fluorophenyl)acetonitrile **1e** (40.5 mg, 0.3 mmol) and (bromomethylene)cyclohexane **2d** (157.6 mg, 0.9 mmol). The crude product was chromatographed on silica gel to give the product **3ed** (57.1 mg, 83% yield for 10% Pd/20% L; 53.6 mg, 78% yield for 5% Pd/10% L) as a colorless oil. R_f = 0.50 (petroleum ether:ethyl acetate = 20:1). ^{1}H NMR (400 MHz, Chloroform-*d*) δ 7.49 (dd, *J* = 8.8, 5.2 Hz, 2H), 7.07 (t, *J* = 8.4 Hz, 2H), 6.58 (d, *J* = 10.0 Hz, 1H), 2.78 – 2.68 (m, 1H), 1.83 – 1.70 (m, 5H), 1.48 – 1.34 (m, 2H), 1.28 – 1.17 (m, 3H) ppm. $^{13}C\{^1H\}$ NMR (100 MHz, Chloroform-*d*) δ 163.1 (d, $^1J_{C\text{-}F}$ = 247.7 Hz), 152.1, 152.0, 129.6 (d, $^4J_{C\text{-}F}$ = 3.3 Hz), 127.6 (d, $^3J_{C\text{-}F}$ = 8.4 Hz), 116.7, 116.0 (d, $^2J_{C\text{-}F}$ = 21.8 Hz), 112.9, 41.5, 32.2, 25.7, 25.4 ppm. IR (thin film): 2929, 2853, 2219, 1603, 1511, 1449, 1415, 1351, 1304, 1290, 1238, 1163, 1104, 953, 835 cm^{-1}. HRMS calc'd for $C_{15}H_{16}FNNa^+$ 252.1159, found 252.1158 [M+Na]$^+$.

(*Z*)-2-(4-chlorophenyl)-3-cyclohexylacrylonitrile (3fd)

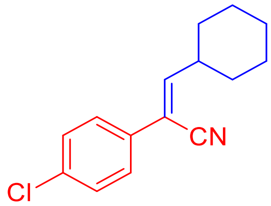

The reaction was performed following the General Procedure with 2-(4-chlorophenyl)acetonitrile **1f** (45.5 mg, 0.3 mmol) and (bromomethylene)cyclohexane **2d** (157.6 mg, 0.9 mmol). The crude product was chromatographed on silica gel to give the product **3fd** (70.0 mg, 95% yield for 10% Pd/20% L; 58.2 mg, 79% yield for 5% Pd/10% L) as a colorless oil. R_f = 0.50 (petroleum ether:ethyl acetate = 20:1). ^{1}H NMR (400 MHz, Chloroform-*d*) δ 7.45 (d, *J* = 8.8 Hz, 2H), 7.35 (d, *J* = 8.8 Hz, 2H), 6.64 (d, *J* = 10.0 Hz, 1H), 2.79 – 2.69 (m, 1H), 1.82 – 1.70 (m, 5H), 1.44 – 1.34 (m, 2H), 1.29 – 1.17 (m, 3H) ppm. $^{13}C\{^1H\}$ NMR (100 MHz, Chloroform-*d*) δ 152.6, 134.9, 131.9, 129.2, 127.0, 116.5, 112.9, 41.6, 32.2, 25.7, 25.3 ppm. IR (thin film): 2929, 2853, 2220, 1616, 1595, 1493, 1449, 1406, 1339, 1096, 1014, 952, 906, 828 cm^{-1}. HRMS calc'd for $C_{15}H_{17}ClN^+$ 246.1044, found 246.1042 [M+H]$^+$.

(*Z*)-2-(4-bromophenyl)-3-cyclohexylacrylonitrile (3gd)

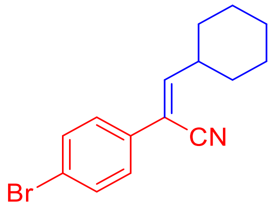

The reaction was performed following the General Procedure with 2-(4-bromophenyl)acetonitrile **1g** (58.8 mg, 0.3 mmol) and (bromomethylene)cyclohexane **2d** (157.6 mg, 0.9 mmol). The crude product was chromatographed on silica gel to give the product **3gd** (43.5 mg, 50% yield) as a colorless oil. R_f = 0.57 (petroleum ether:ethyl acetate = 20:1). ^{1}H NMR (400 MHz, Chloroform-*d*) δ 7.45 – 7.12 (m, 4H), 6.57 (d, *J* = 10.0 Hz, 1H), 2.71 – 2.63 (m, 1H), 1.75 – 1.63 (m, 5H), 1.38 – 1.29 (m, 2H), 1.21 – 1.13 (m, 3H) ppm. ^{13}C{^{1}H} NMR (100 MHz, Chloroform-*d*) δ 152.8, 132.4, 132.2, 127.3, 123.1, 116.4, 113.0, 41.6, 32.2, 25.7, 25.3 ppm. IR (thin film): 2928, 2852, 2219, 1589, 1489, 1448, 1402, 1337, 1261, 1076, 1010, 989, 951, 823 cm^{-1}. HRMS calc'd for $C_{15}H_{17}BrN^+$ 290.0539, found 290.0539 [M+H]$^+$.

(*Z*)-3-cyclohexyl-2-(naphthalen-2-yl)acrylonitrile (3hd)

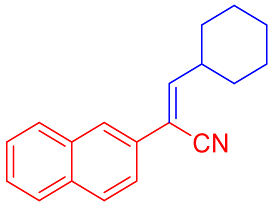

The reaction was performed following the General Procedure with 2-(naphthalen-2-yl)acetonitrile **1h** (50.2 mg, 0.3 mmol) and (bromomethylene)cyclohexane **2d** (157.6 mg, 0.9 mmol). The crude product was chromatographed on silica gel to give the product **3hd** (52.5 mg, 67% yield) as a colorless oil. R_f = 0.50 (petroleum ether:ethyl acetate = 20:1). ^{1}H NMR (400 MHz, Chloroform-*d*) δ 8.09 (d, *J* = 8.0 Hz, 1H), 7.88 (td, *J* = 7.6, 1.6 Hz, 2H), 7.60 – 7.52 (m, 2H), 7.48 – 7.41 (m, 2H), 6.49 (d, *J* = 10.0 Hz, 1H), 3.00 – 2.86 (m, 1H), 1.97 – 1.92 (m, 2H), 1.85 – 1.73 (m, 3H), 1.52 – 1.41 (m, 2H), 1.28 (m, 3H) ppm. ^{13}C{^{1}H} NMR (100 MHz, Chloroform-*d*) δ 158.5, 133.8, 132.4, 130.8, 129.6, 128.7, 127.3, 127.0, 126.4, 125.4, 124.5, 117.5, 112.0, 41.4, 32.3, 25.7, 25.4 ppm. IR (thin film): 3061, 2927, 2852, 2214, 1593, 1509, 1448, 1396, 1347, 1250, 1176, 1020, 976, 801, 776 cm^{-1}. HRMS calc'd for $C_{19}H_{19}NNa^+$ 248.1410, found 248.1408 [M+Na]$^+$.

(*Z*)-3-cyclohexyl-2-(1-methyl-1*H*-indol-3-yl)acrylonitrile (3id)

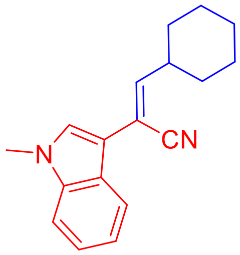

The reaction was performed following the General Procedure with 2-(1-methyl-1*H*-indol-3-yl)acetonitrile **1i** (51.1 mg, 0.3 mmol) and (bromomethylene)cyclohexane **2d** (157.6 mg, 0.9 mmol). The crude product was chromatographed on silica gel to give the product **3id** (73.8 mg, 93% yield for 10% Pd/20% L; 62.7 mg, 79% yield for 5% Pd/10% L) as a colorless oil. R_f = 0.40 (petroleum ether:ethyl

acetate = 10:1). ^{1}H NMR (400 MHz, Chloroform-*d*) δ 7.82 (d, *J* = 8.0 Hz, 1H), 7.33 – 7.26 (m, 3H), 7.23 – 7.18 (m, 1H), 6.59 (dd, *J* = 10.0, 1.2 Hz, 1H), 3.76 (d, *J* = 1.6 Hz, 3H), 2.79 – 2.68 (m, 1H), 1.88 – 1.70 (m, 5H), 1.46 – 1.35 (m, 2H), 1.32 – 1.21 (m, 3H) ppm. ^{13}C{^{1}H} NMR (100 MHz, Chloroform-*d*) δ 147.4, 137.8, 128.9, 124.8, 122.7, 120.6, 119.8, 117.6, 110.01, 109.98, 107.5, 41.4, 33.0, 32.8, 25.8, 25.5 ppm. IR (thin film): 3052, 2926, 2851, 2218, 1617, 1535, 1475, 1448, 1381, 1335, 1210, 1138, 1156, 1108, 972, 740 cm^{-1}. HRMS calc'd for $C_{18}H_{20}N_2Na^+$ 287.1519, found 287.1519 [M+Na]$^+$.

(*Z*)-3-cyclohexyl-2-(thiophen-2-yl)acrylonitrile (3jd)

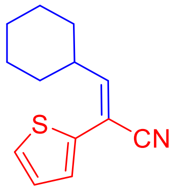

The reaction was performed following the General Procedure with 2-(thiophen-2-yl)acetonitrile **1j** (37.0 mg, 0.3 mmol) and (bromomethylene)cyclohexane **2d** (157.6 mg, 0.9 mmol). The crude product was chromatographed on silica gel to give the product **3jd** (30.6 mg, 47% yield) as a colorless oil. R_f = 0.40 (petroleum ether:ethyl acetate = 20:1). ^{1}H NMR (400 MHz, Chloroform-*d*) δ 7.23 – 7.20 (m, 2H), 7.01 (dd, *J* = 5.2, 3.6 Hz, 1H), 6.48 (d, *J* = 10.0 Hz, 1H), 2.73 – 2.63 (m, 1H), 1.82 – 1.70 (m, 5H), 1.43 – 1.33 (m, 2H), 1.28 – 1.17 (m, 3H) ppm. ^{13}C{^{1}H} NMR (100 MHz, Chloroform-*d*) δ 150.2, 137.9, 127.9, 126.4, 125.6, 115.8, 108.5, 41.3, 32.2, 25.7, 25.4 ppm. IR (thin film): 3110, 2928, 2852, 2223, 1612, 1448, 1433, 1333, 1255, 1233, 1047, 974, 901, 848, 831, 700 cm^{-1}. HRMS calc'd for $C_{13}H_{15}NSNa^+$ 240.0817, found 240.0819 [M+Na]$^+$.

Gram scale synthesis of 3aa

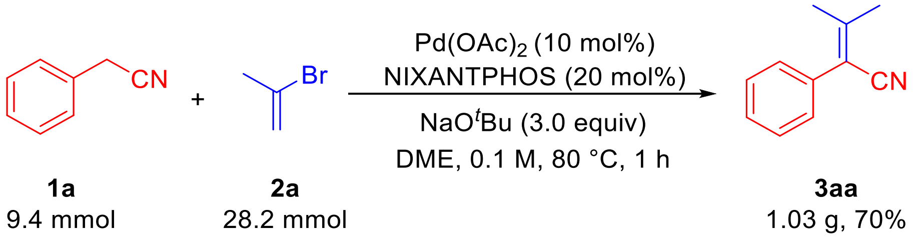

An oven-dried 250 mL Schlenk tube equipped with a stir bar was charged with $Pd(OAc)_2$ (211.0 mg, 10 mol%), NIXANTPHOS (1.0 g, 20 mol%), NaO^{t}Bu (2.7 g, 28.2 mmol), 2-phenylacetonitrile **1a** (1.1 g, 9.4 mmol) and 2-bromoprop-1-ene **2** (3.4 g, 28.2 mmol) under a nitrogen atmosphere in a glove box. Then a solvent of 94 mL dry DME was added to the reaction vial. The vial was sealed with a cap, removed from the glove box, and stirred for 1 h at 80 °C. The reaction mixture was cooled to room temperature and then opened to air, quenched with 5 mL of H_2O, diluted with 15 mL of ethyl acetate, and filtered over a 3 cm pad of silica. The pad was rinsed with ethyl acetate (3 X 15 mL), and the combined organic solutions were concentrated in vacuo. The crude product was chromatographed on silica gel (petroleum ether:ethyl acetate = 20:1) to give the product **3aa** (1.03 g, 70% yield) as a colorless oil.

Derivatization studies of product 3aa

a. Reduction of carbon-carbon double bond

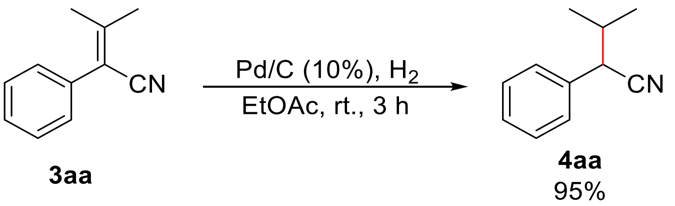

Pd/C (10%, 21 mg) was added to a solution of **3aa** (62.9 mg, 0.4 mmol) in EtOAc (15 mL) at room temperature in a nonreactant borosilicate glass vessel under the shaker hydrogenation apparatus. Hydrogen gas was installed to the reaction mixture at room temperature. The reaction mixture was stirred at room temperature for 3 h. Then the reaction mixture was diluted with 3 mL of ethyl acetate, and filtered over a 2 cm pad of silica. The pad was rinsed with ethyl acetate (3 X 3 mL), and the combined organic solutions were concentrated in vacuo. The crude product was chromatographed on silica gel (petroleum ether:ethyl acetate = 20:1) to give the product **4aa** (60.5 mg, 95% yield) as a colorless oil.

3-methyl-2-phenylbutanenitrile (4aa)

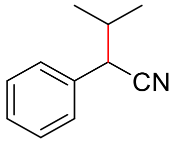

R_f = 0.34 (petroleum ether:ethyl acetate = 20:1). ^{1}H NMR (400 MHz, Chloroform-*d*) δ 7.29 – 7.19 (m, 5H), 3.57 (d, *J* = 8.4 Hz, 1H), 2.09 – 1.98 (m, 1H), 1.00 – 0.91 (m, 6H) ppm. ^{13}C{^{1}H} NMR (100 MHz, Chloroform-*d*) δ 135.1, 129.0, 128.1, 128.0, 120.0, 45.3, 34.0, 21.0, 19.0 ppm. IR (thin film): 3065, 3031, 2976, 2934, 2238, 1602, 1493, 1454, 1390, 1172, 1033, 913, 750, 700 cm^{-1}. HRMS calc'd for $C_{11}H_{14}N^+$ 160.1121, found 160.1121 $[M+H]^+$.

b. Reduction of nitrile group

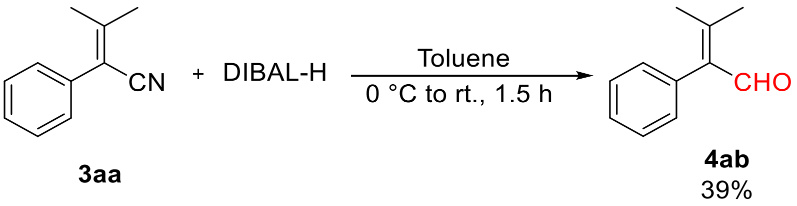

To a solution of **3aa** (78.6 mg, 0.5 mmol) in toluene (5 mL) at 0 °C was added a solution of DIBAL-H in hexane (1.5 M, 1.5 mL, 1.0 mmol). The resulting mixture was stirred at 0 °C for 5 min and then warmed to room temperature for 1.5 h. After completion of this reaction, the reaction mixture was quenched with brine (5 mL). Then, the mixture was separated and the aqueous phases were extracted with ethyl acetate (3 X 10 mL). The organic phases were combined and dried over anhydrous sodium sulfate. After filtration and removal of the solvent, the residue was chromatographed on silica gel (petroleum ether:ethyl acetate = 10:1) to give the product **4ab** (31.2 mg, 39%) as a colorless oil.

3-methyl-2-phenylbut-2-enal (4ab)

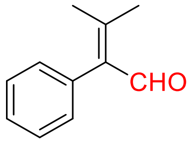

R_f = 0.34 (petroleum ether:ethyl acetate = 5:1). ^{1}H NMR (400 MHz, Chloroform-*d*) δ 10.21 (s, 1H), 7.31

(dd, J = 8.0, 6.4 Hz, 2H), 7.29 – 7.21 (m, 1H), 7.03 – 7.00 (m, 2H), 2.29 (s, 3H), 1.79 (s, 3H) ppm. $^{13}C\{^{1}H\}$ NMR (100 MHz, Chloroform-*d*) δ 190.5, 156.6, 139.1, 136.1, 130.0, 128.4, 127.4, 25.2, 19.8 ppm. IR (thin film): 3081, 3022, 2918, 2859, 1672, 1598, 1492, 1443, 1372, 1309, 1072, 722, 700, 607 cm^{-1}; HRMS calc'd for $C_{11}H_{13}NO^{+}$ 161.0961, found 161.0956 $[M+H]^{+}$.

c. Hydrolysis of nitrile group

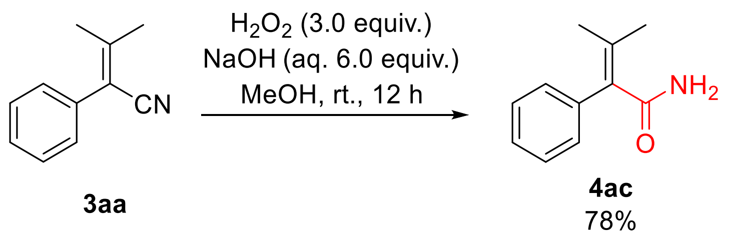

To a solution of **3aa** (78.6 mg, 0.5 mmol) in MeOH (5 mL) at room temperature was added H_2O_2 (30%, 45 μL, 1.5 mmol) and 50% NaOH aq. (240 μL, 3.0 mmol). The reaction mixture was then stirred at room temperature for 12 h. A saturated aqueous solution of ammonium chloride (10 mL) was added. The aqueous phase was extracted with dichloromethane (3 X 10 mL) and the combined organic phases were dried over anhydrous sodium sulfate. After filtration, the solvents were removed under reduced pressure and the residue was chromatographed on silica gel (petroleum ether:ethyl acetate = 5:1) to give the product **4ac** (68.3 mg, 78%) as a colorless oil.

3-methyl-2-phenylbut-2-enamide (4ac)

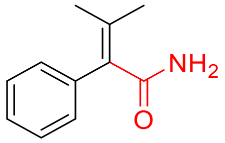

R_f = 0.68 (petroleum ether:ethyl acetate = 3:1). ^{1}H NMR (400 MHz, Chloroform-*d*) δ 7.31 (dd, J = 8.0, 6.8 Hz, 2H), 7.31 – 7.17 (m, 3H), 5.72 (s, 1H), 5.20 (s, 1H), 2.12 (s, 3H), 1.61 (s, 3H) ppm. $^{13}C\{^{1}H\}$ NMR (100 MHz, Chloroform-*d*) δ 171.4, 142.9, 138.8, 131.79, 131.76, 129.6, 128.8, 127.6, 23.5, 23.4, 22.6 ppm. IR (thin film): 3871, 3177, 2934, 1641, 1499, 1442, 1399, 1261, 1125, 1070, 863, 762, 701 cm^{-1}. HRMS calc'd for $C_{11}H_{14}NO^{+}$ 176.1070, found 176.1072 $[M+H]^{+}$.

d. Epoxidation of carbon-carbon double bond and hydrolysis of nitrile group

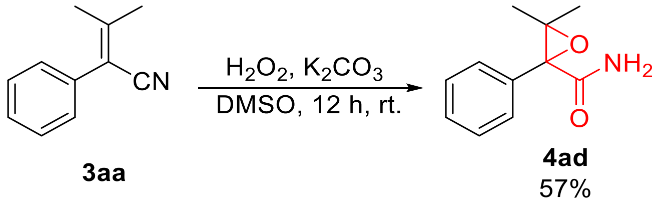

To a solution of **3aa** (78.6 mg, 0.5 mmol) in DMSO (5 mL) at room temperature was added K_2CO_3 (14.0 mg, 0.1 mmol) and H_2O_2 (30%, 140 μL, 1.10 mmol). The reaction mixture was then stirred at room temperature for 12 h. A saturated aqueous solution of ammonium chloride (10 mL) was added. The aqueous phase was extracted with dichloromethane (3 X 15 mL) and the combined organic phases were dried over anhydrous sodium sulfate. After filtration, the solvents were removed under reduced pressure and the residue was chromatographed on silica gel (petroleum ether:ethyl acetate = 5:1) to give the product **4ad** (54.5 mg, 57%) as a white solid.

3,3-dimethyl-2-phenyloxirane-2-carboxamide (4ad)

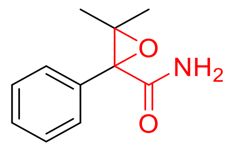

Mp 161 – 162 ºC. R_f = 0.5 (petroleum ether:ethyl acetate = 3:1). ^{1}H NMR (400 MHz, Chloroform-*d*) δ 7.52 – 7.57 (m, 2H), 7.36 – 7.30 (m, 3H), 6.47 (d, *J* = 31.3 Hz, 2H), 1.51 (s, 3H), 1.04 (s, 3H) ppm. ^{13}C{^{1}H} NMR (100 MHz, Chloroform-*d*) δ 172.1, 134.5, 128.2, 128.1, 127.4, 69.5, 66.3, 20.9, 20.7 ppm. IR (thin film): 3409, 3170, 2990, 2929, 1652, 1492, 1448, 1416, 1377, 1220, 1091, 1073, 948, 890, 755, 698 cm^{-1}. HRMS calc'd for $C_{11}H_{13}NO_2Na^+$ 214.0838, found 214.0840 $[M+Na]^+$.

NMR Spectra

Figure S1. ^{1}**H NMR spectra (400 MHz, Chloroform-*d*) of 3-methyl-2-phenylbut-2-enenitrile (3aa).**

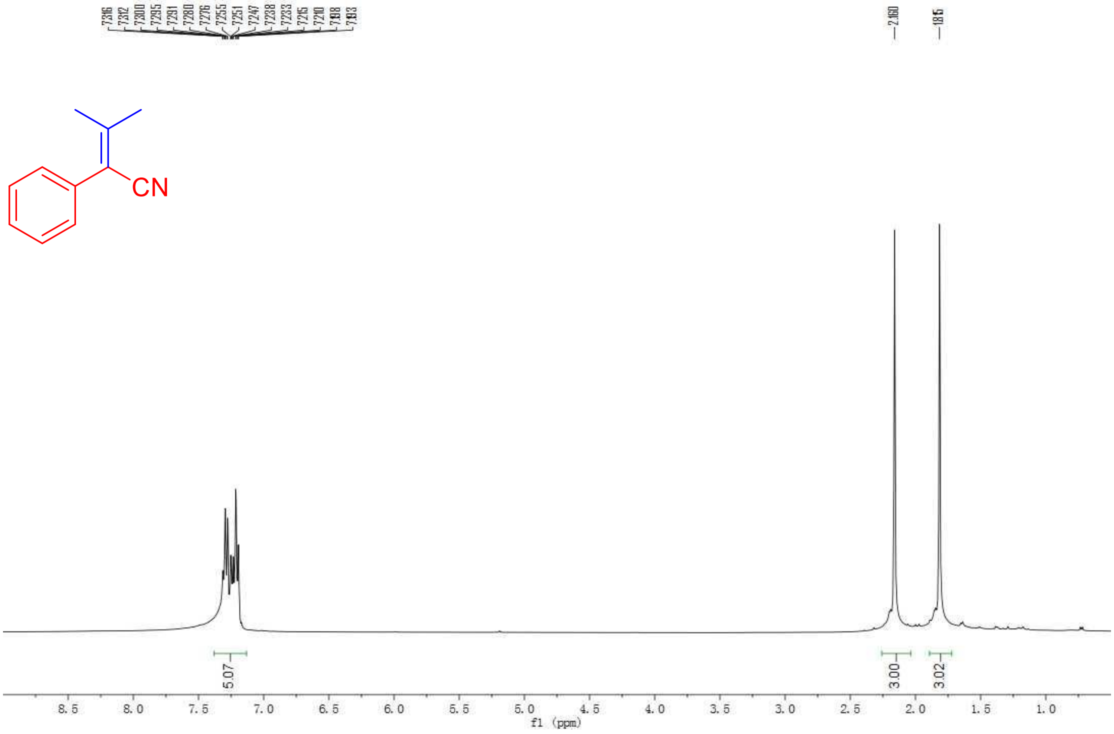

Figure S2. ^{13}**C{**^{1}**H} NMR spectra (100 MHz, Chloroform-*d*) of 3-methyl-2-phenylbut-2-enenitrile (3aa).**

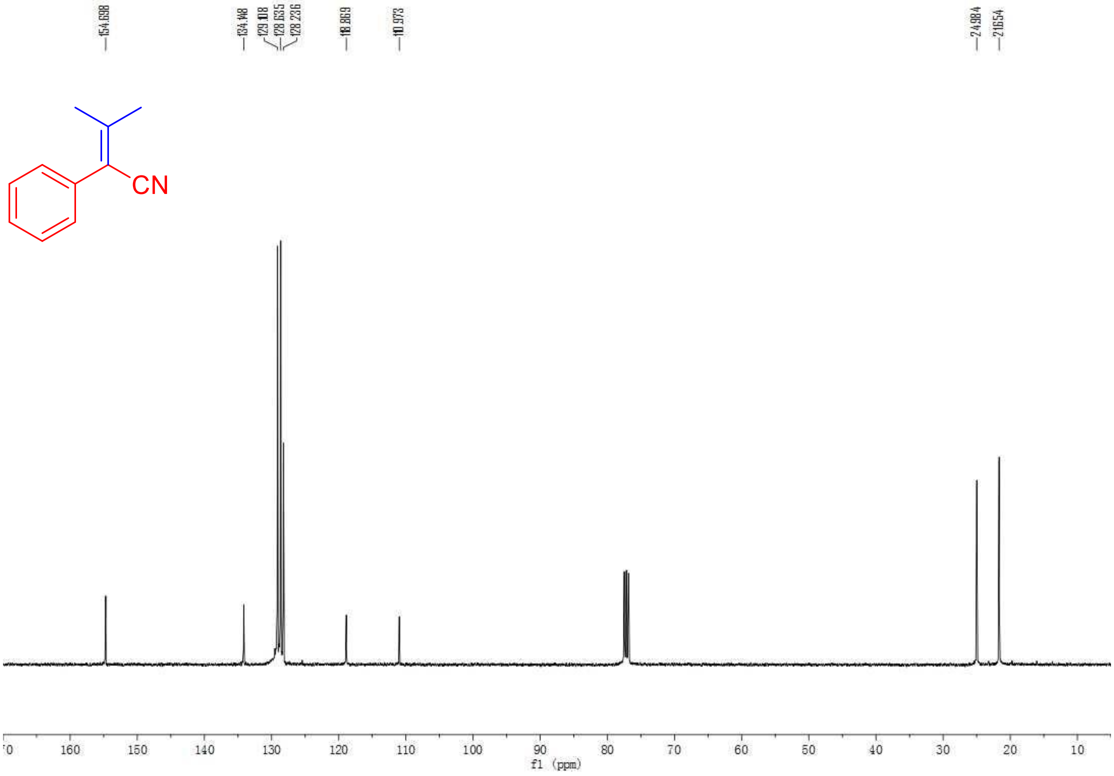

Figure S3. ^{1}H NMR spectra (400 MHz, Chloroform-*d*) of (*Z*)-4-methyl-2-phenylpent-2-enenitrile (3ab).

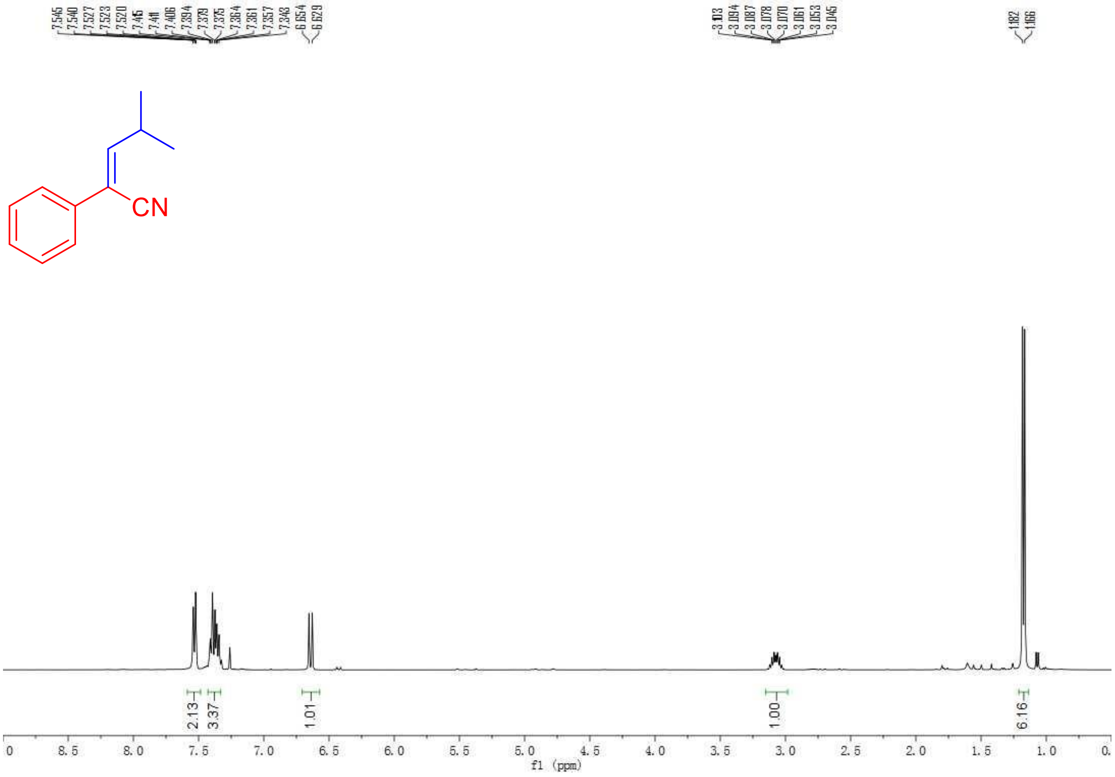

Figure S4. ^{13}C{^{1}H} NMR spectra (100 MHz, Chloroform-*d*) of (*Z*)-4-methyl-2-phenylpent-2-enenitrile (3ab).

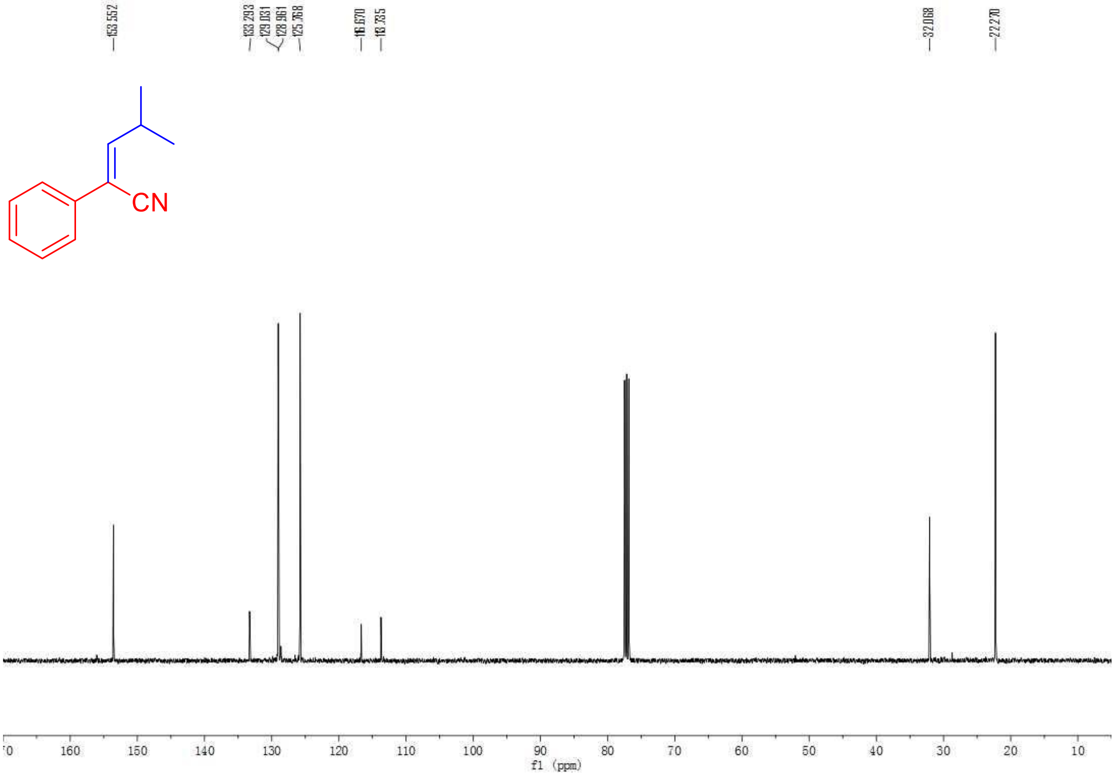

Figure S5. ^{1}H NMR spectra (400 MHz, Chloroform-*d*) of (*Z*)-2,4-diphenylpent-2-enenitrile (3ac).

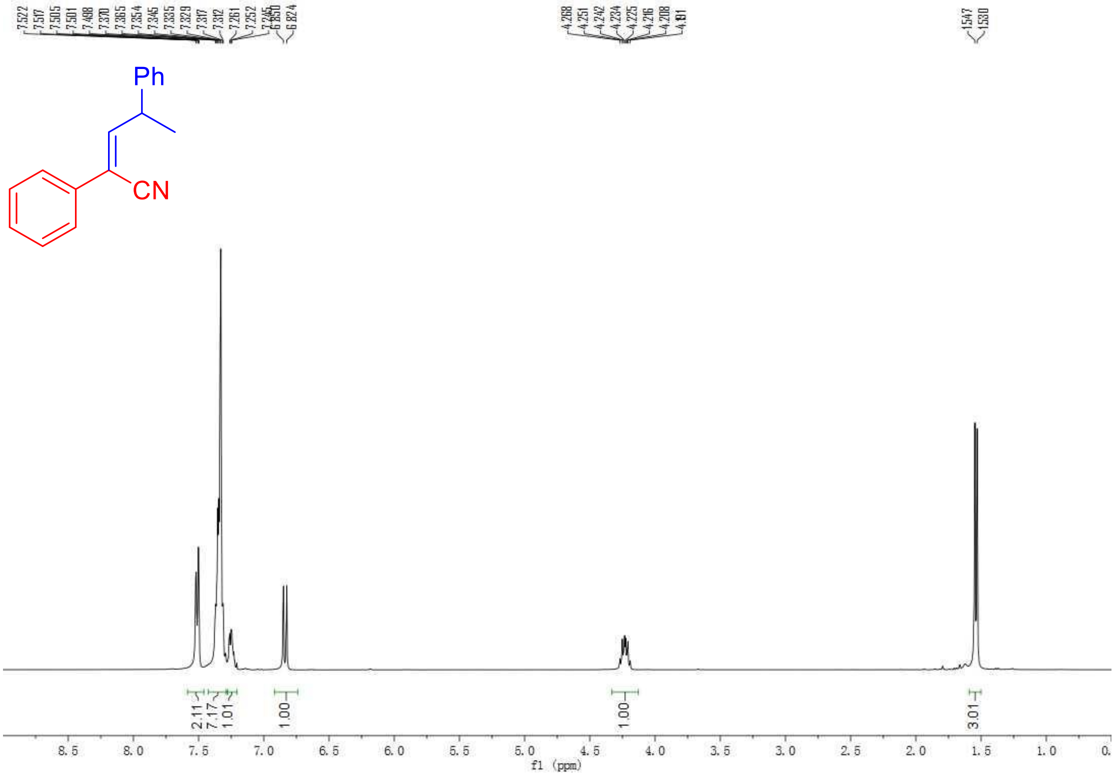

Figure S6. ^{13}C{^{1}H} NMR spectra (100 MHz, Chloroform-*d*) of (*Z*)-2,4-diphenylpent-2-enenitrile (3ac).

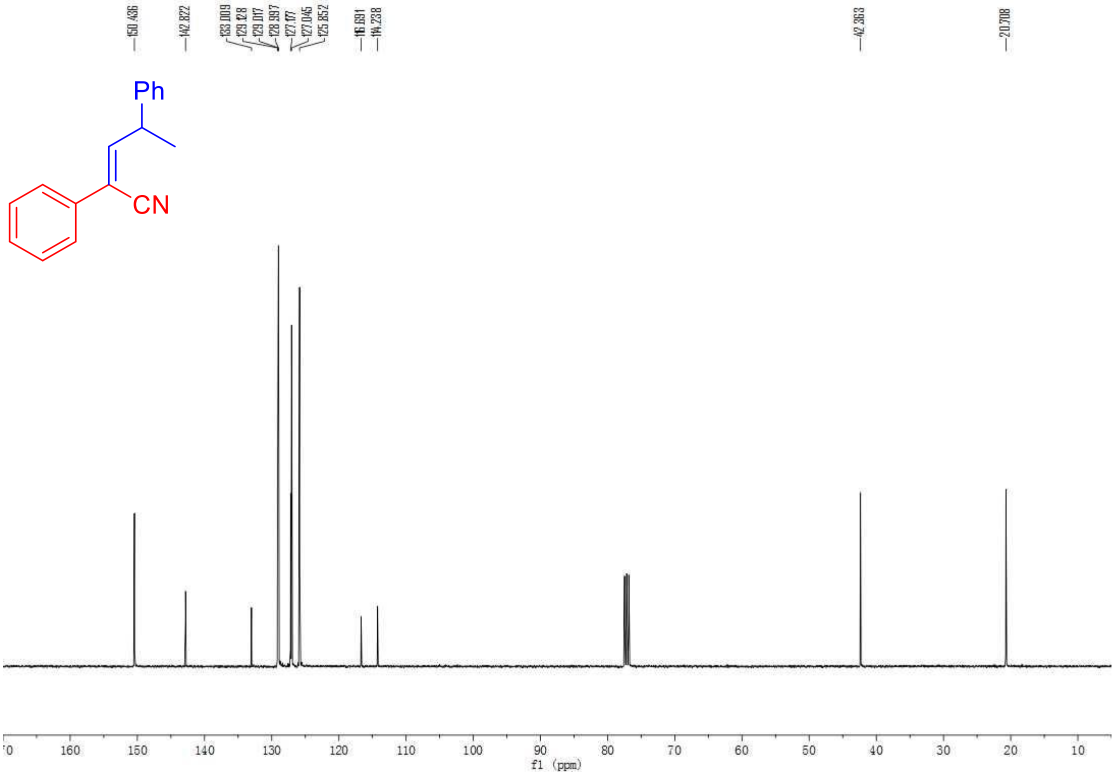

Figure S7. ^{1}H **NMR spectra (400 MHz, Chloroform-*d*) of (*Z*)-3-cyclohexyl-2-phenylacrylonitrile (3ad).**

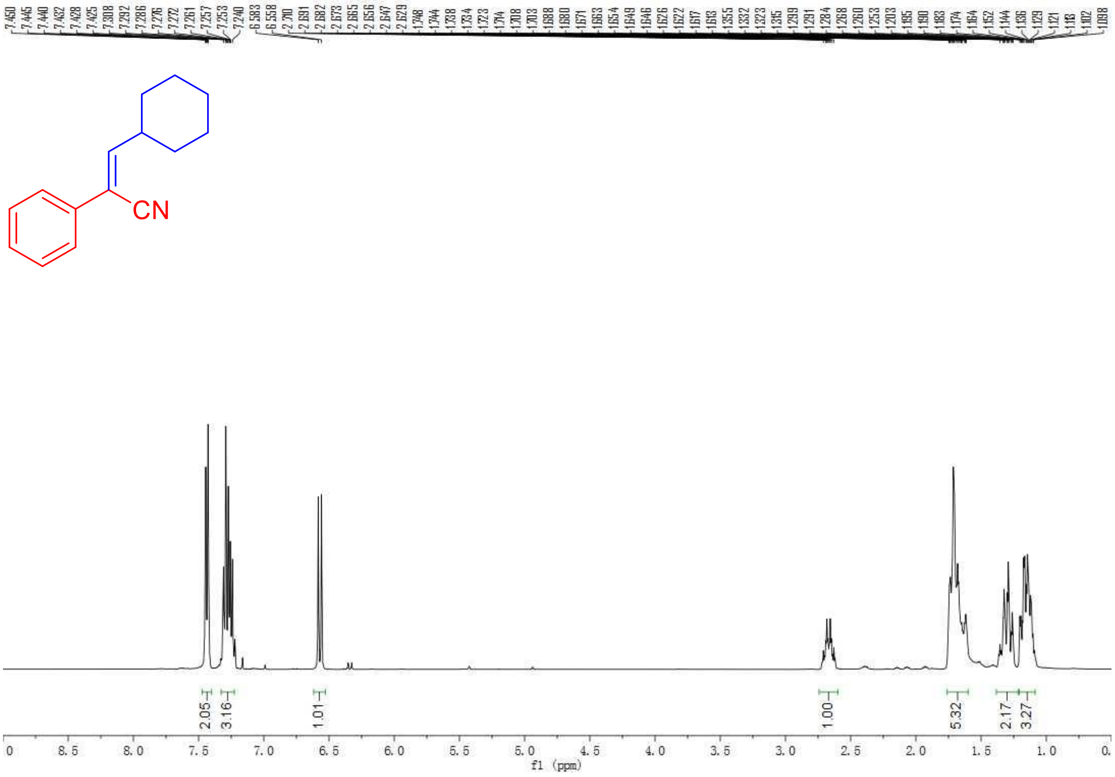

Figure S8. $^{13}C\{^{1}H\}$ **NMR spectra (100 MHz, Chloroform-*d*) of (*Z*)-3-cyclohexyl-2-phenylacrylonitrile (3ad).**

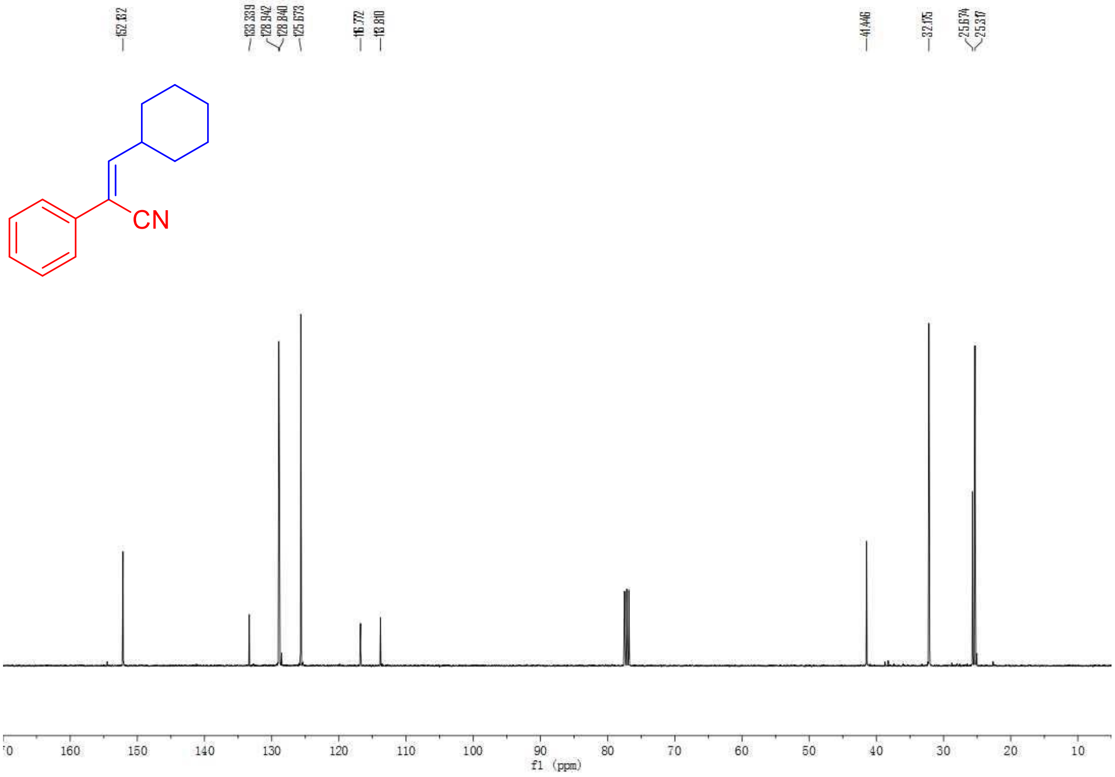

Figure S9. ^{1}H **NMR spectra (400 MHz, Chloroform-*d*) of (3-methyl-2-phenyl-1λ^{3}-pent-2-en-1-ylidene)-λ^{2}-azane (3ae).**

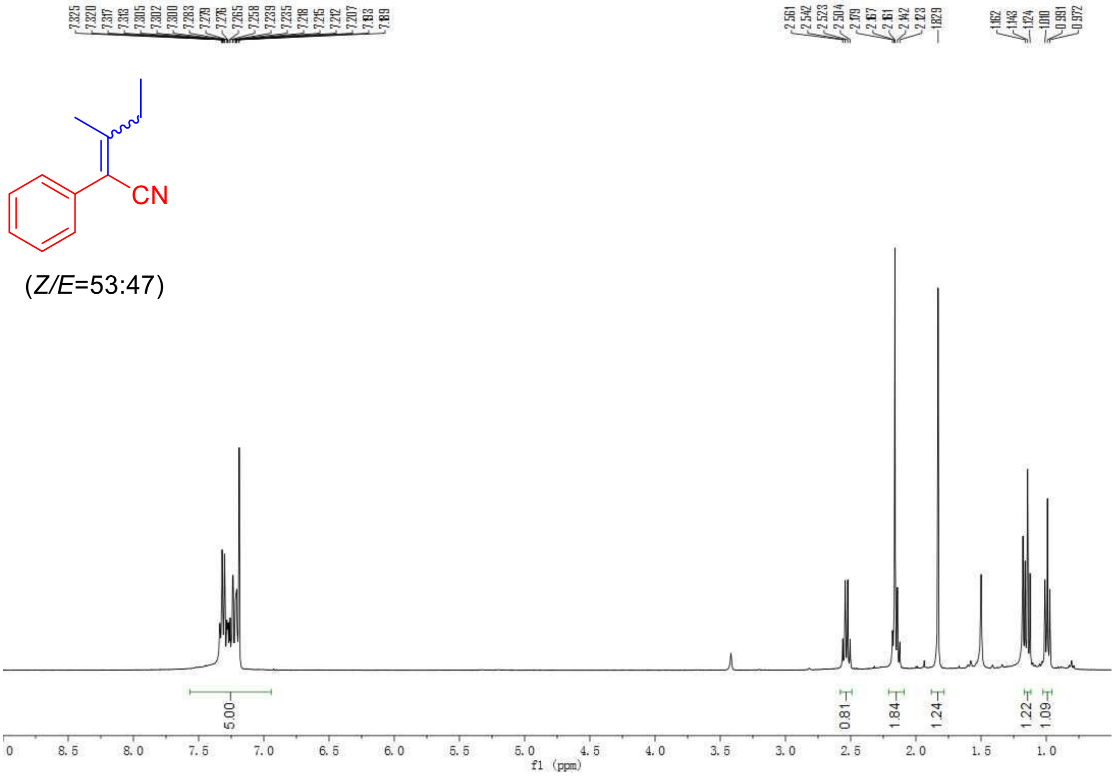

Figure S10. ^{13}C{^{1}H} **NMR spectra (100 MHz, Chloroform-*d*) of (3-methyl-2-phenyl-1λ^{3}-pent-2-en-1-ylidene)-λ^{2}-azane (3ae).**

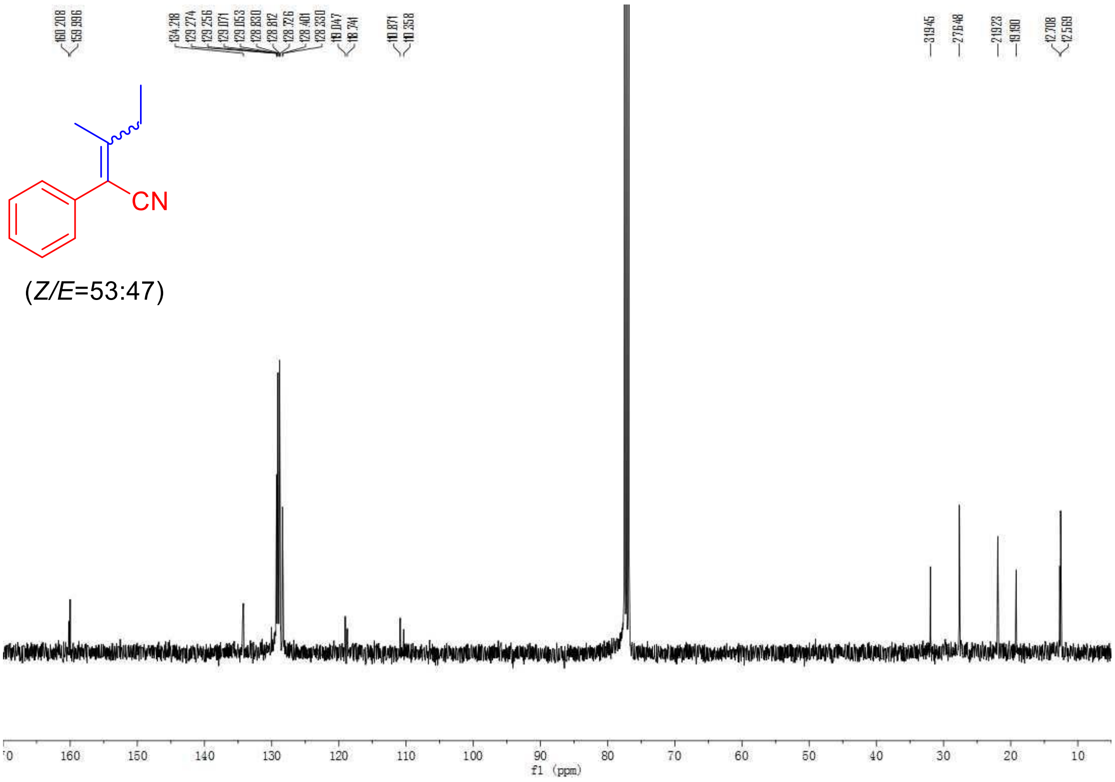

Figure S11. ^{1}H **NMR spectra (400 MHz, Chloroform-*d*) of 3-methyl-2-phenylpent-2-enenitrile (3af).**

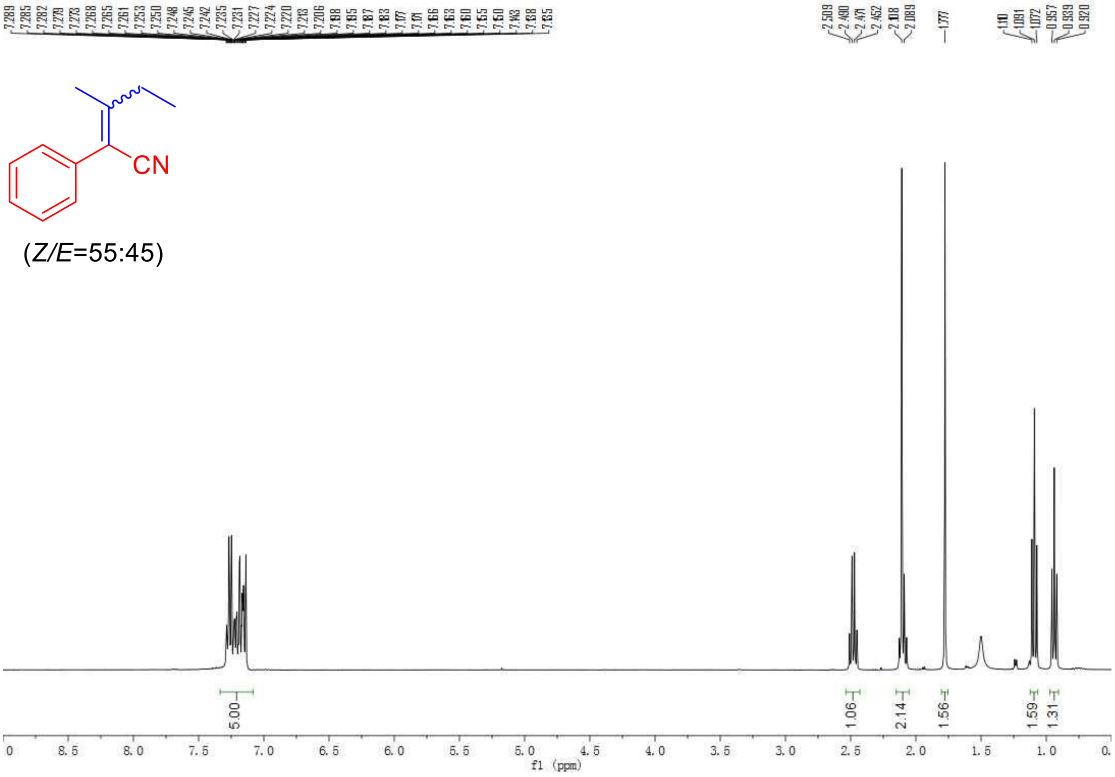

Figure S12. $^{13}C\{^{1}H\}$ **NMR spectra (100 MHz, Chloroform-*d*) of 3-methyl-2-phenylpent-2-enenitrile (3af).**

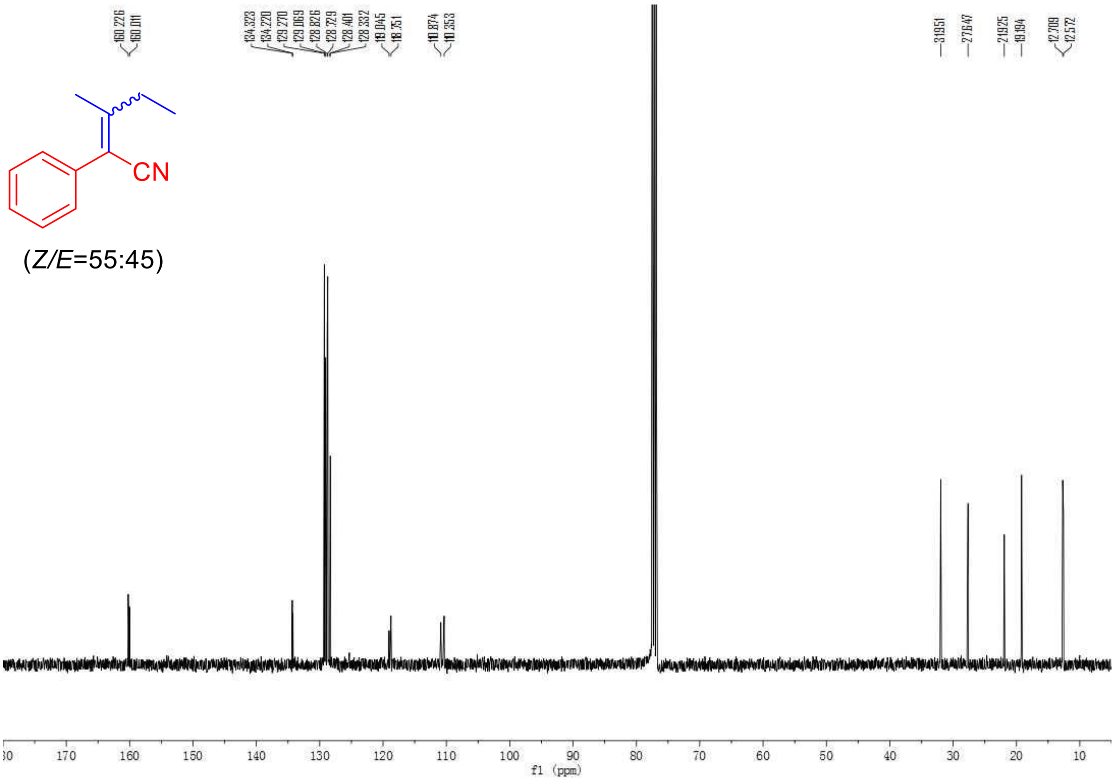

Figure S13. **^{1}H NMR spectra (400 MHz, Chloroform-*d*) of 3,4-dimethyl-2-phenylpent-2-enenitrile (3ag).**

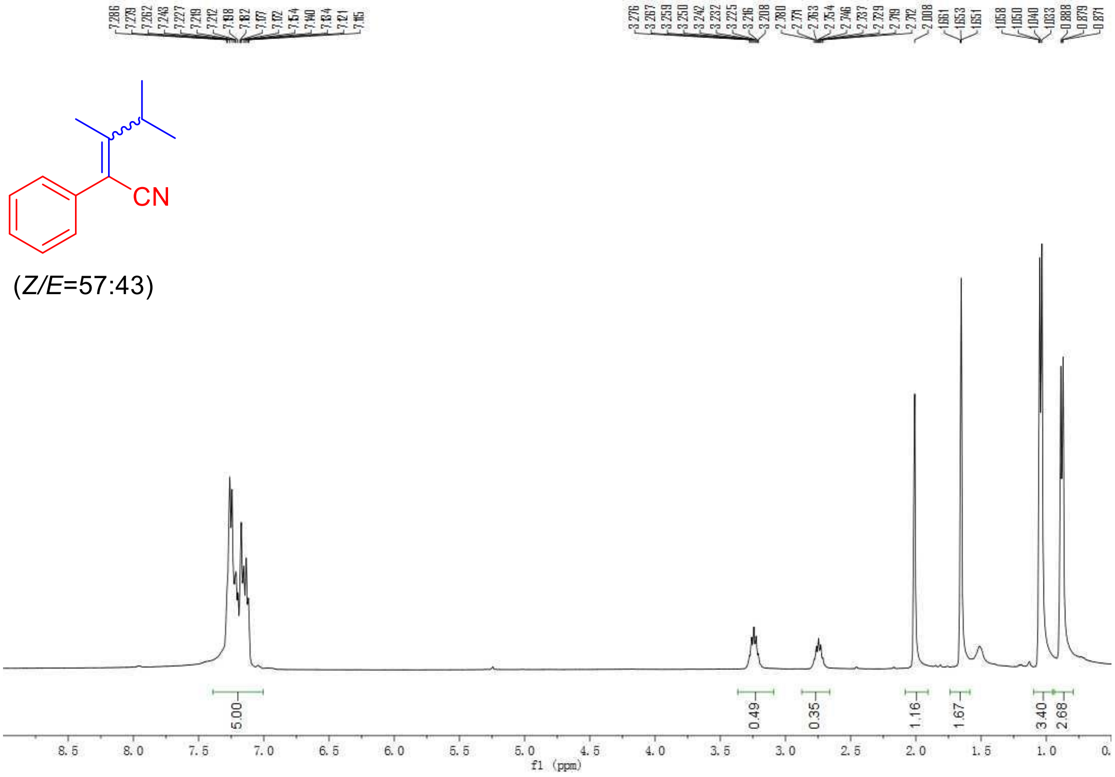

Figure S14. **^{13}C{^{1}H} NMR spectra (100 MHz, Chloroform-*d*) of 3,4-dimethyl-2-phenylpent-2-enenitrile (3ag).**

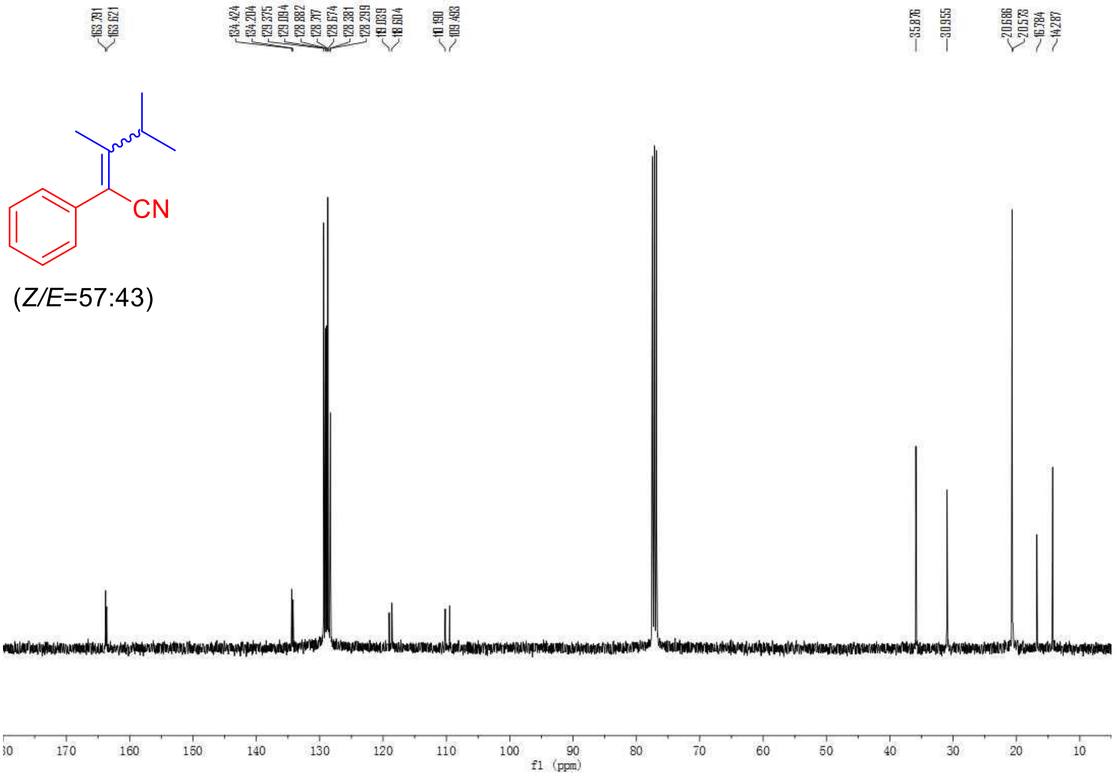

Figure S15. **^{1}H NMR spectra (400 MHz, Chloroform-*d*) of 2-cyclopentylidene-2-phenylacetonitrile (3ah).**

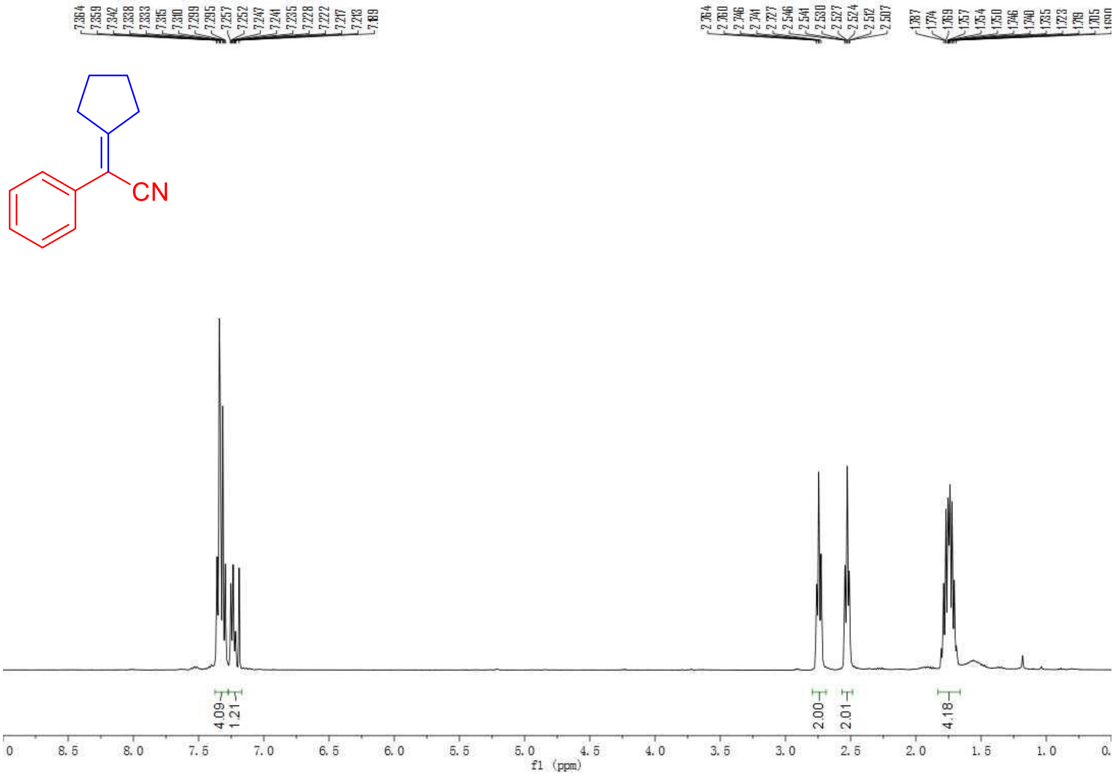

Figure S16. **^{13}C{^{1}H} NMR spectra (100 MHz, Chloroform-*d*) of 2-cyclopentylidene-2-phenylacetonitrile (3ah).**

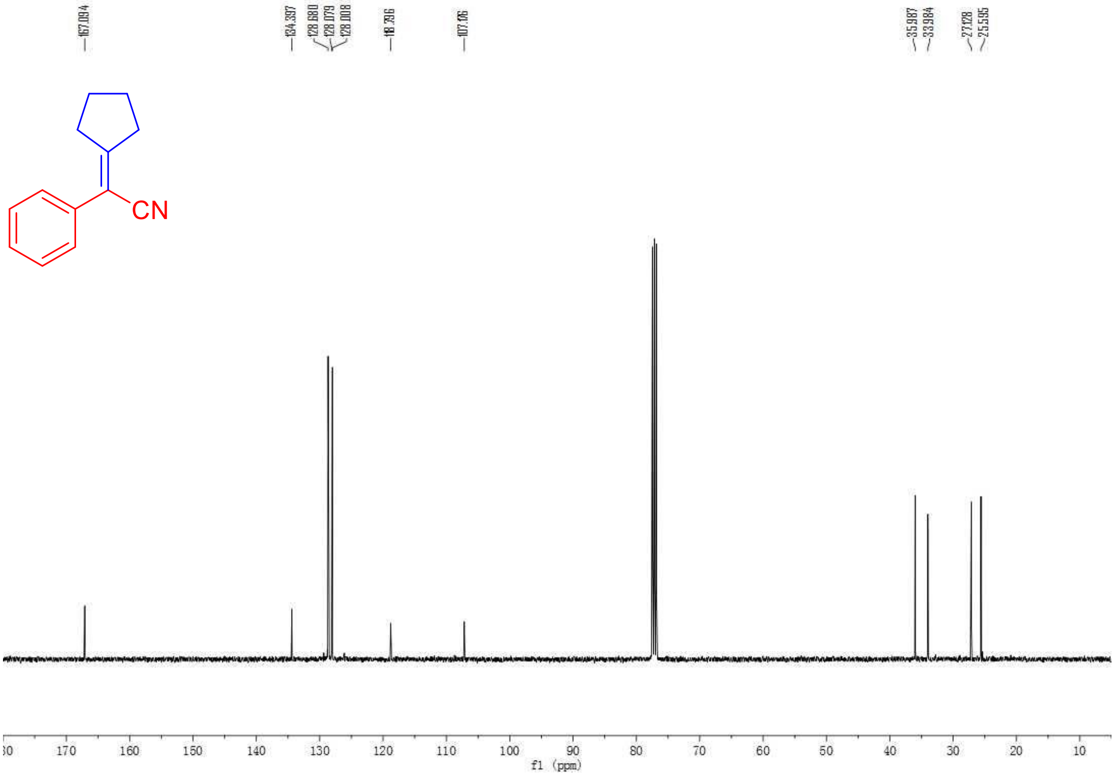

Figure S17. ^{1}H **NMR spectra (400 MHz, Chloroform-*d*) of 2-cyclohexylidene-2-phenylacetonitrile (3ai).**

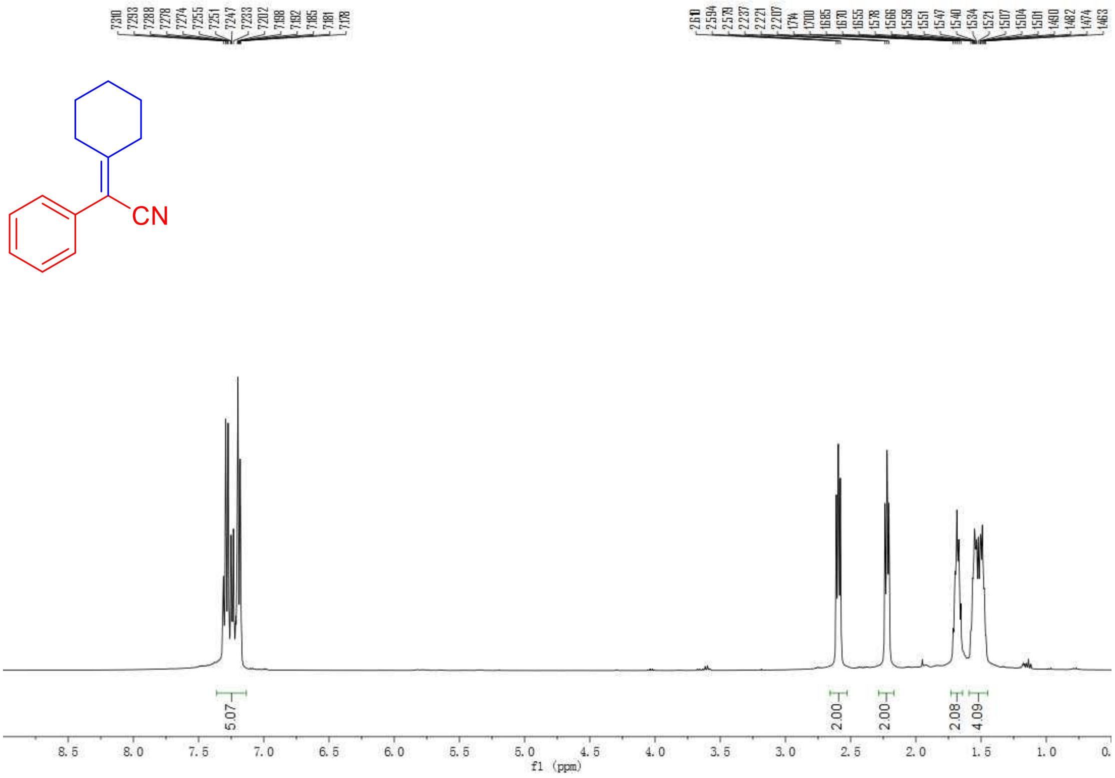

Figure S18. $^{13}C\{^{1}H\}$ **NMR spectra (100 MHz, Chloroform-*d*) of 2-cyclohexylidene-2-phenylacetonitrile (3ai).**

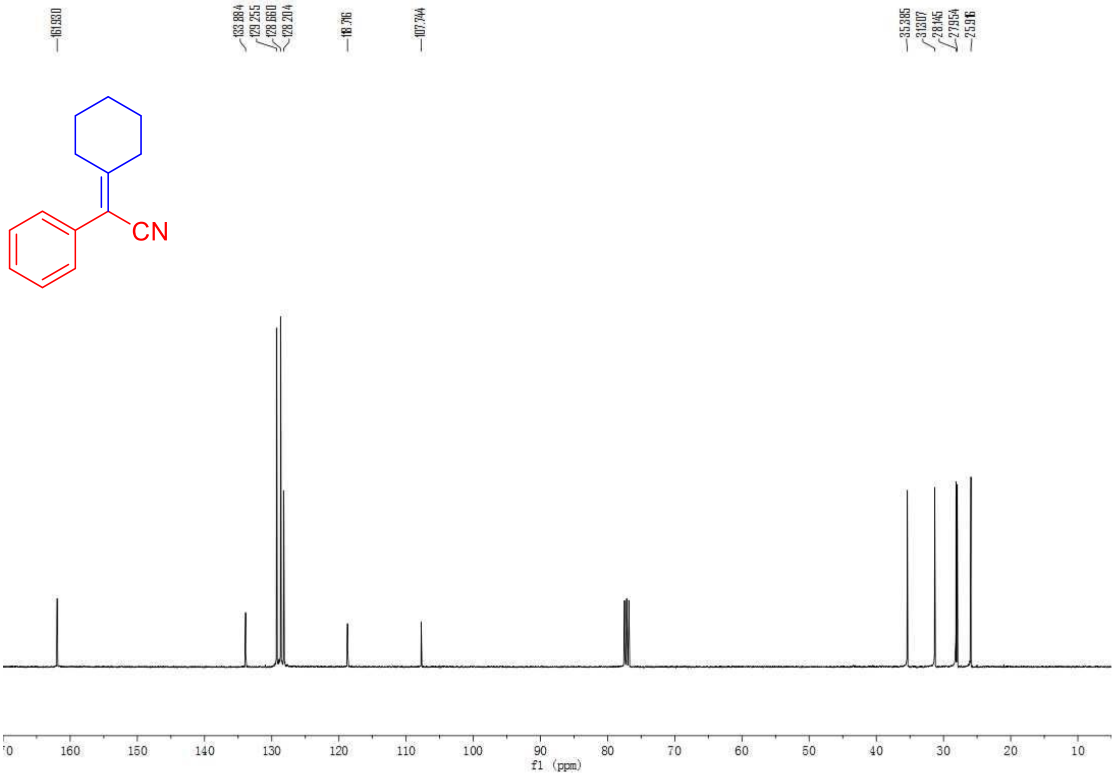

Figure S19. ^{1}H NMR spectra (400 MHz, Chloroform-*d*) of 2-cycloheptylidene-2-phenylacetonitrile (3aj).

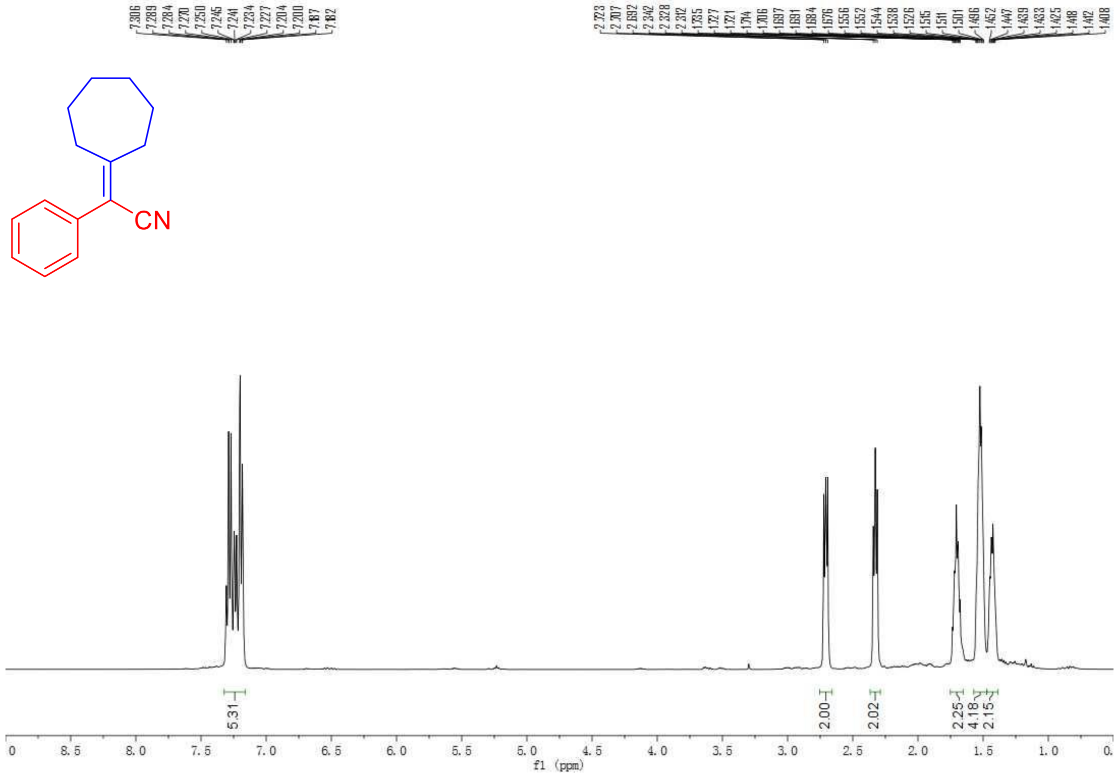

Figure S20. ^{13}C{^{1}H} NMR spectra (100 MHz, Chloroform-*d*) of 2-cycloheptylidene-2-phenylacetonitrile (3aj).

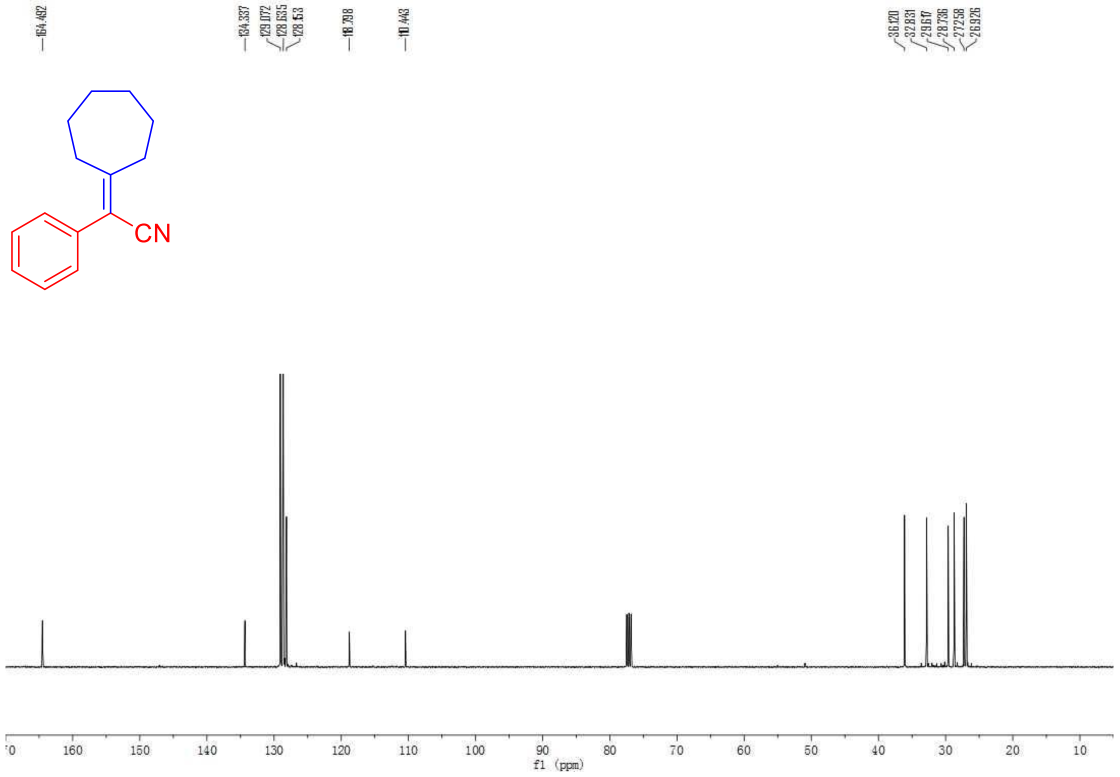

Figure S21. ^{1}H NMR spectra (400 MHz, Chloroform-*d*) of 2-cyclooctylidene-2-phenylacetonitrile (3ak).

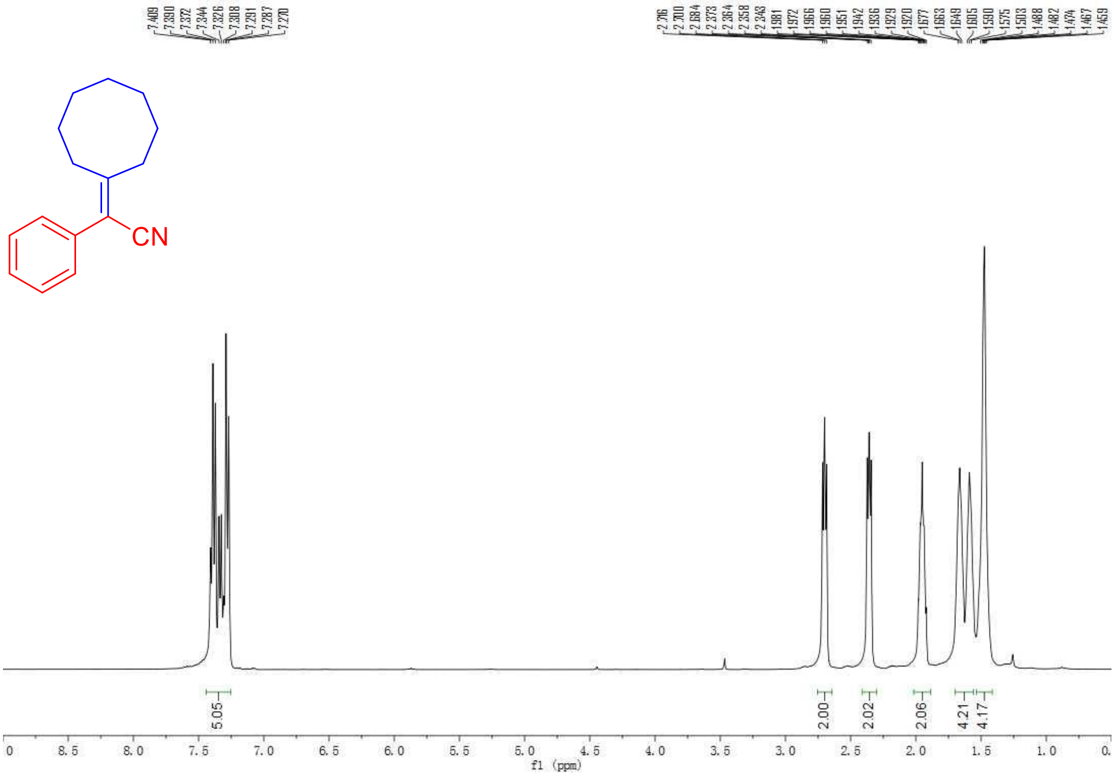

Figure S22. ^{13}C{^{1}H} NMR spectra (100 MHz, Chloroform-*d*) of 2-cyclooctylidene-2-phenylacetonitrile (3ak).

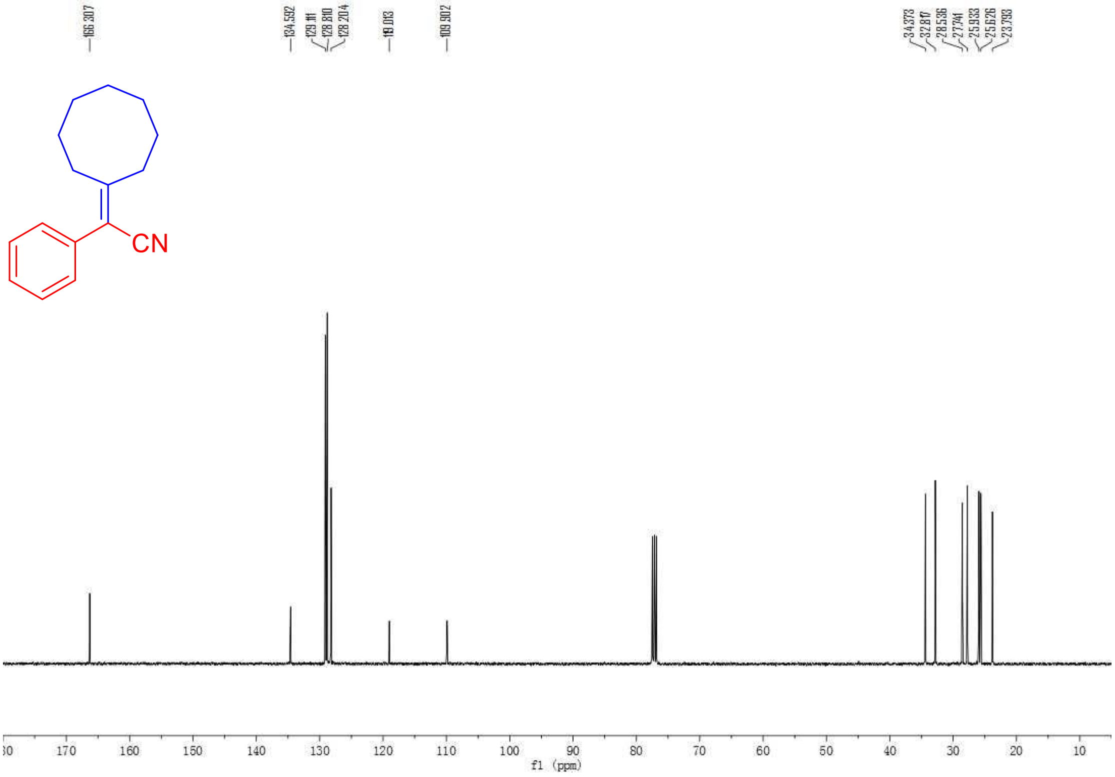

Figure S23. [1]**H NMR spectra (400 MHz, Chloroform-*d*) of (*Z*)-3-cyclohexyl-2-(p-tolyl)acrylonitrile (3bd).**

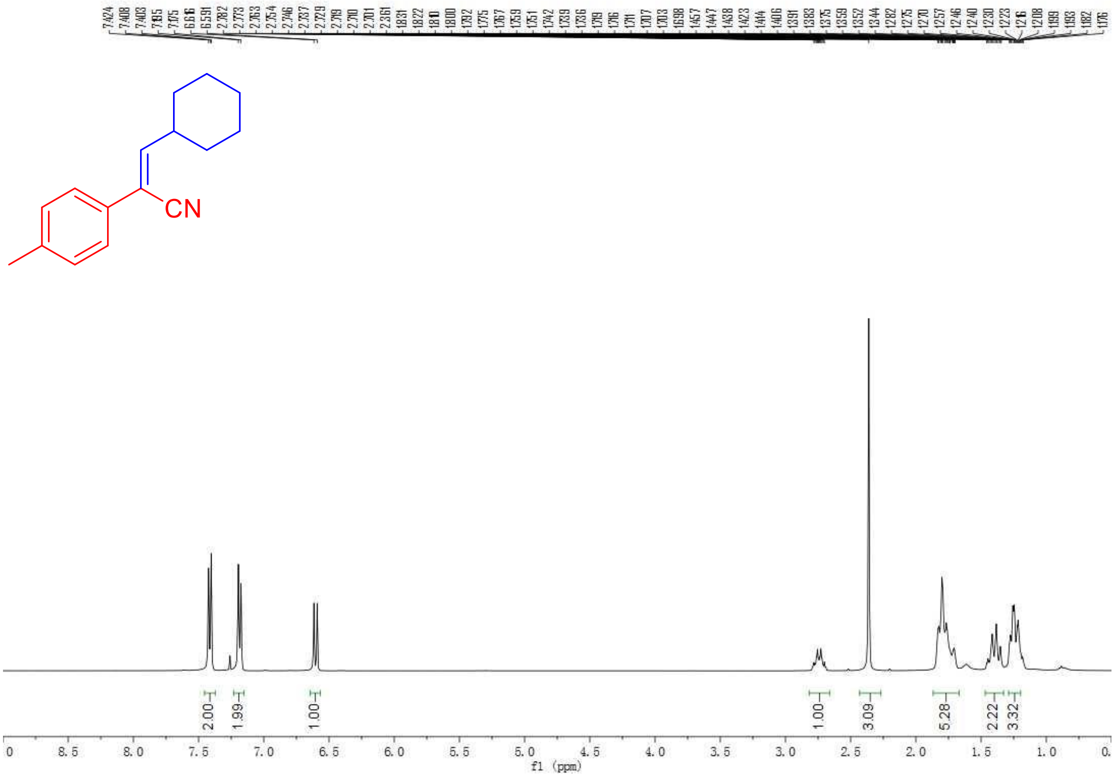

Figure S24. [13]**C{**[1]**H} NMR spectra (100 MHz, Chloroform-*d*) of (*Z*)-3-cyclohexyl-2-(p-tolyl)acrylonitrile (3bd).**

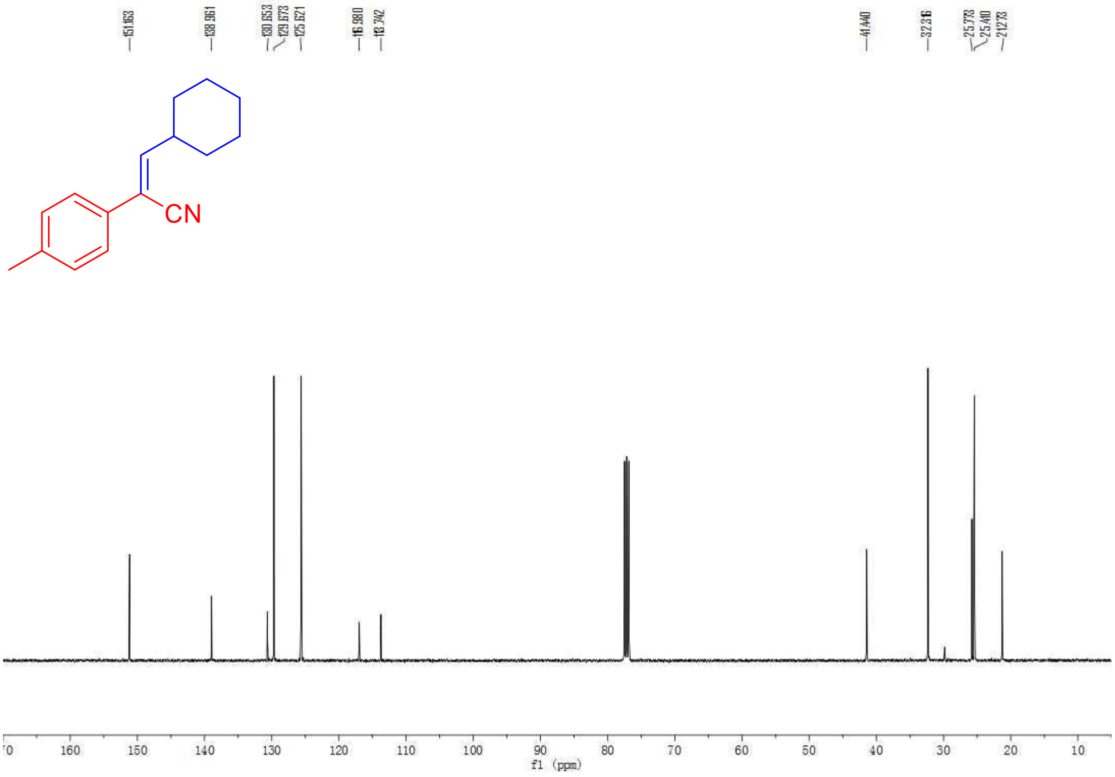

Figure S25. **^{1}H NMR spectra (400 MHz, Chloroform-*d*) of (*Z*)-3-cyclohexyl-2-(*o*-tolyl)acrylonitrile (3cd).**

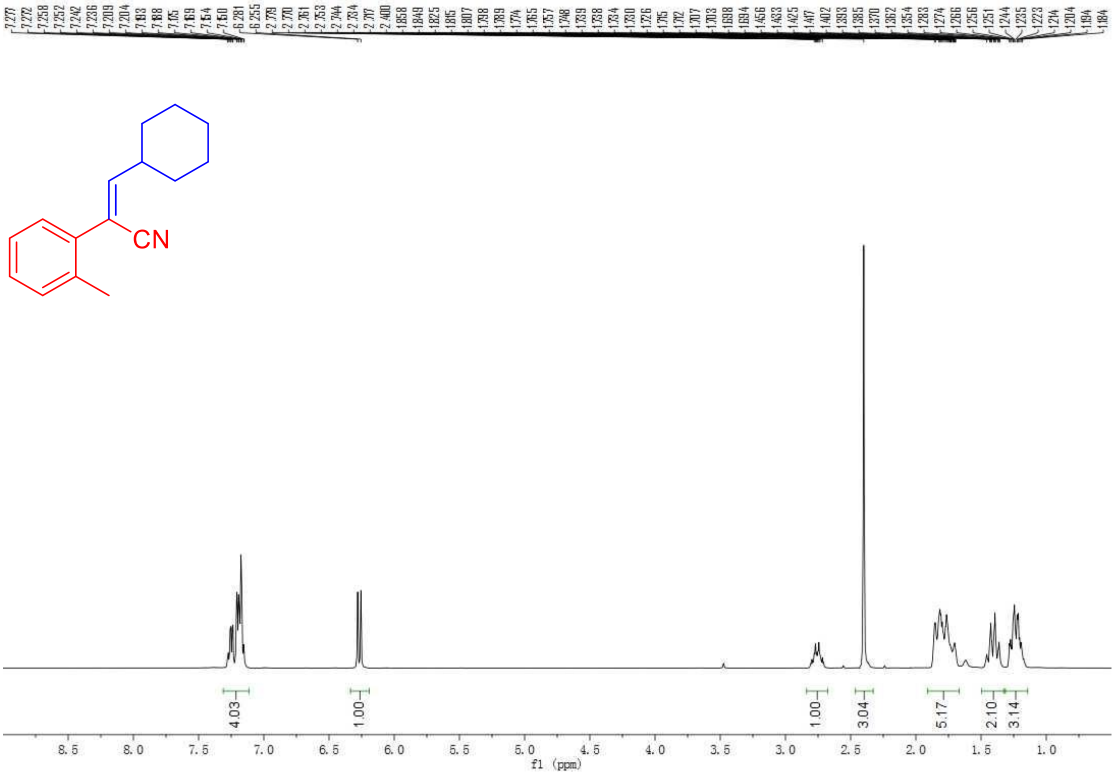

Figure S26. **^{13}C{^{1}H} NMR spectra (100 MHz, Chloroform-*d*) of (*Z*)-3-cyclohexyl-2-(*o*-tolyl)acrylonitrile (3cd).**

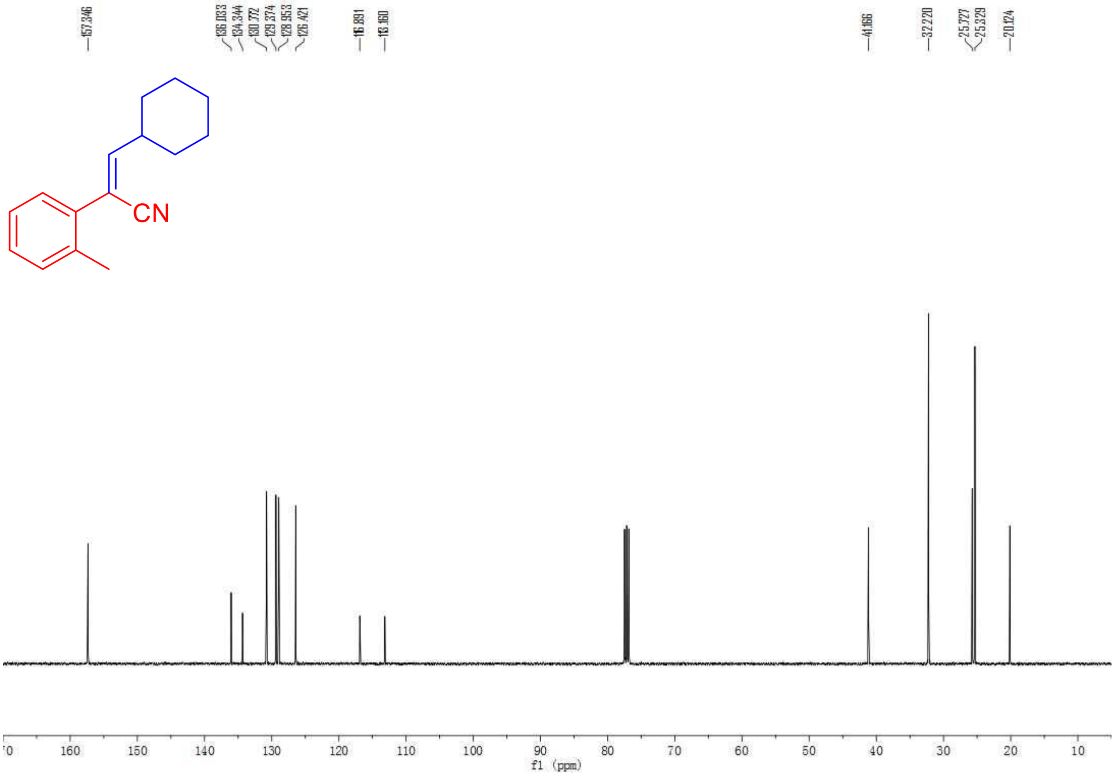

Figure S27. ^{1}H **NMR spectra (400 MHz, Chloroform-*d*) of (*Z*)-3-cyclohexyl-2-(4-methoxyphenyl)acrylonitrile (3dd).**

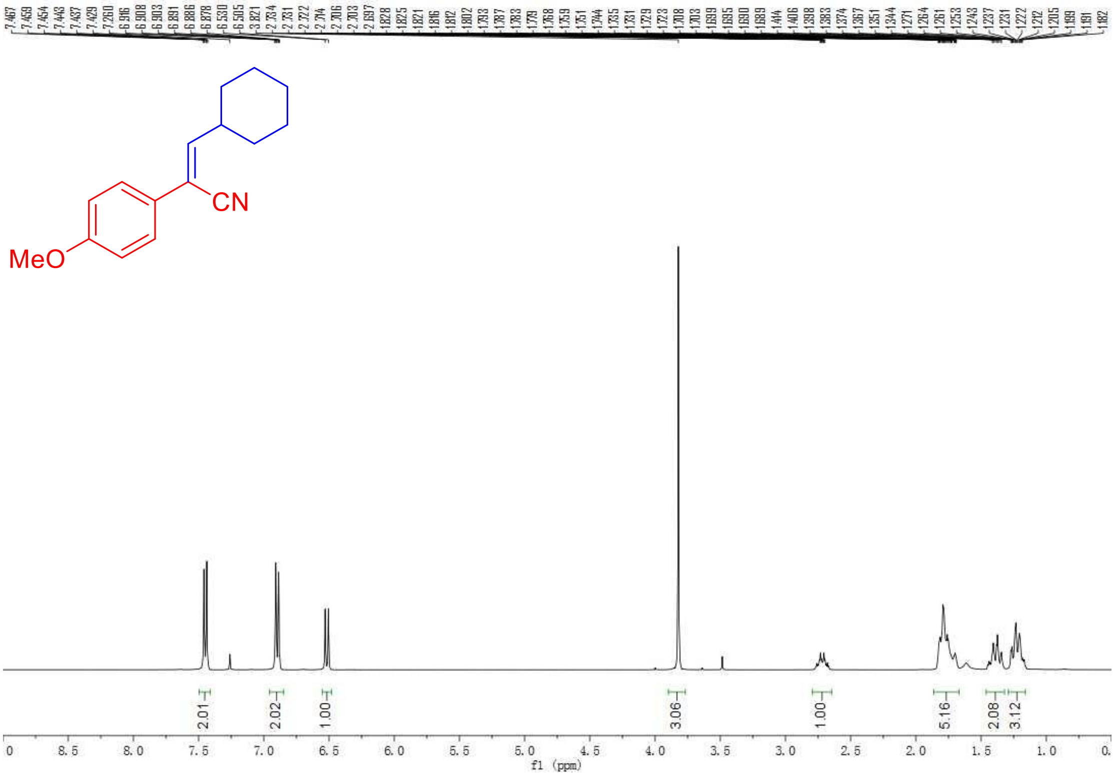

Figure S28. $^{13}C\{^{1}H\}$ **NMR spectra (100 MHz, Chloroform-*d*) of (*Z*)-3-cyclohexyl-2-(4-methoxyphenyl)acrylonitrile (3dd).**

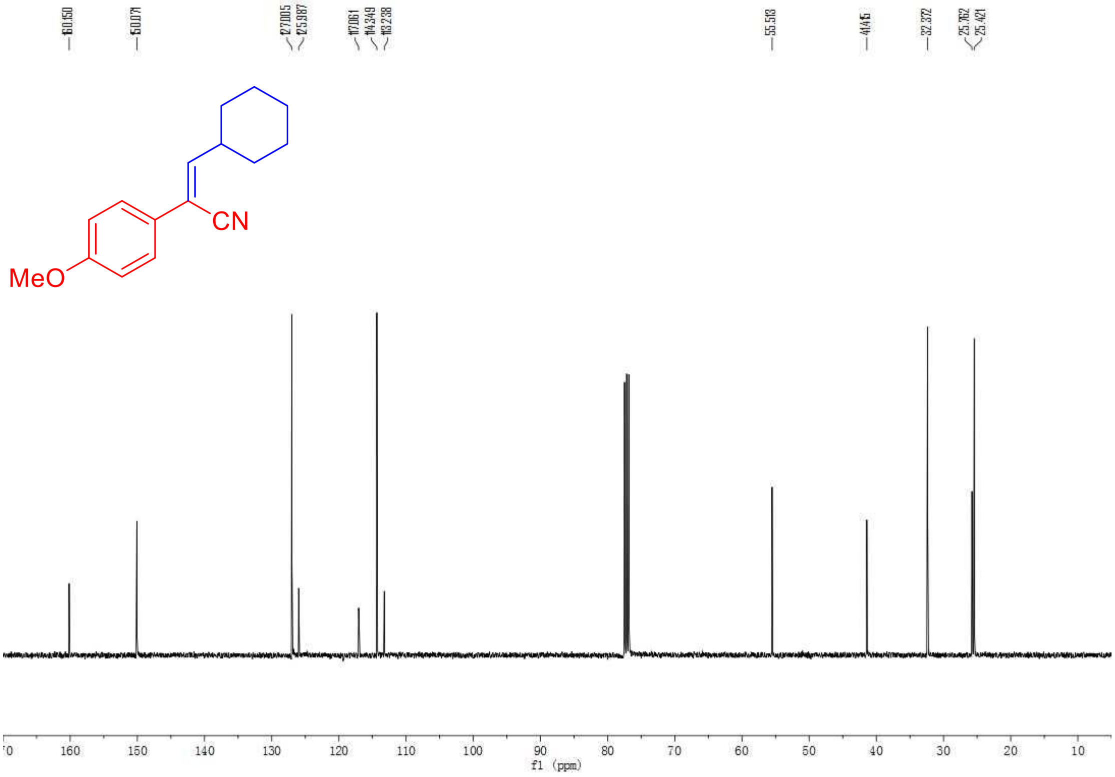

Figure S29. ^{1}H NMR spectra (400 MHz, Chloroform-*d*) of (*Z*)-3-cyclohexyl-2-(4-fluorophenyl)acrylonitrile (3ed).

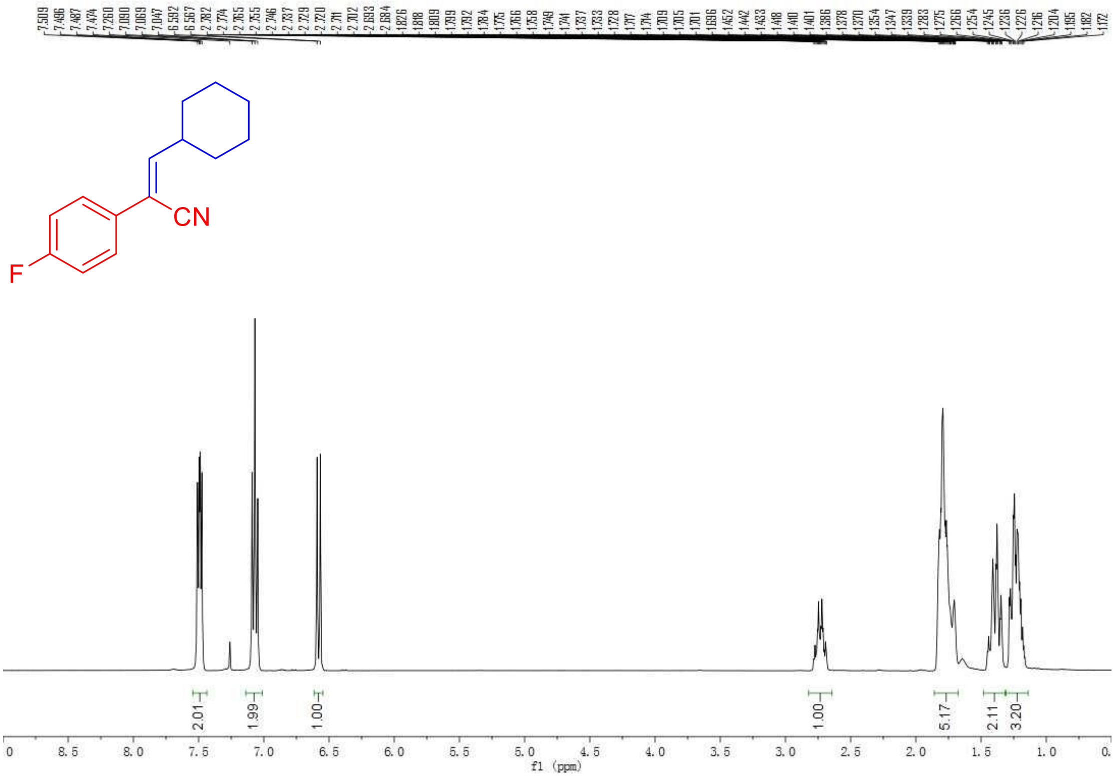

Figure S30. ^{13}C{^{1}H} NMR spectra (100 MHz, Chloroform-*d*) of (*Z*)-3-cyclohexyl-2-(4-fluorophenyl)acrylonitrile (3ed).

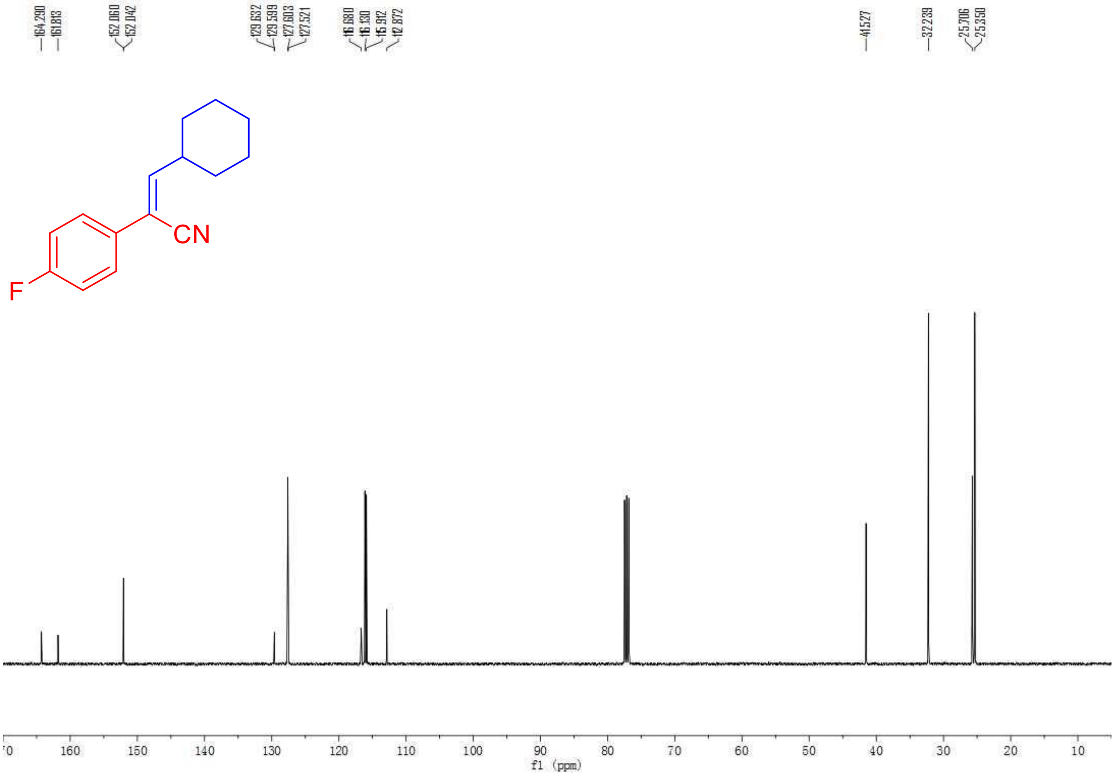

Figure S31. ^{1}H **NMR spectra (400 MHz, Chloroform-*d*) of (*Z*)-2-(4-chlorophenyl)-3-cyclohexylacrylonitrile (3fd).**

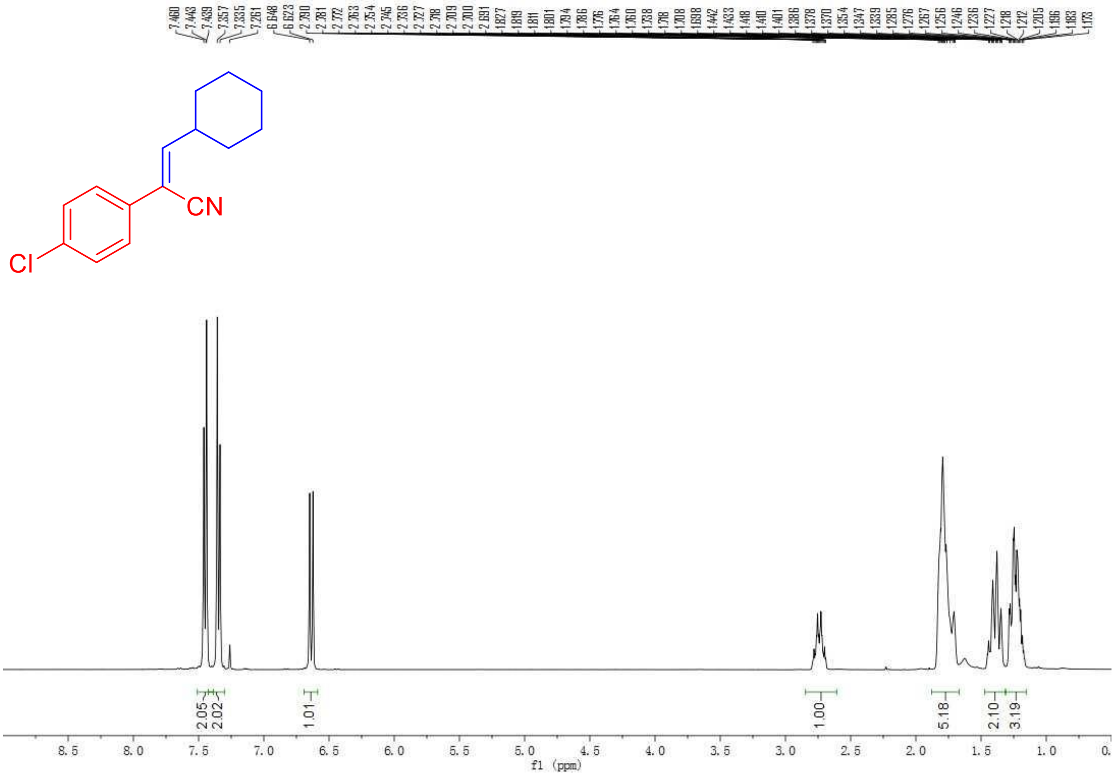

Figure S32. ^{13}C{^{1}H} **NMR spectra (100 MHz, Chloroform-*d*) of (*Z*)-2-(4-chlorophenyl)-3-cyclohexylacrylonitrile (3fd).**

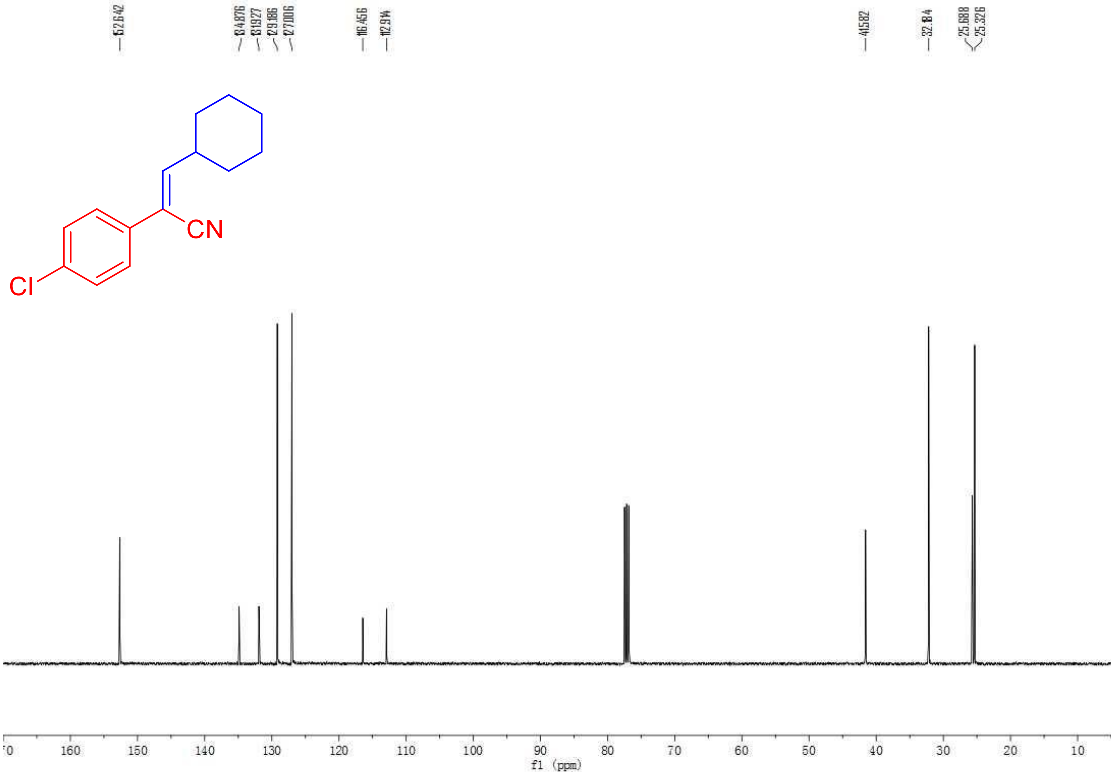

Figure S33. ^{1}H **NMR spectra (400 MHz, Chloroform-*d*) of (*Z*)-2-(4-bromophenyl)-3-cyclohexylacrylonitrile (3gd).**

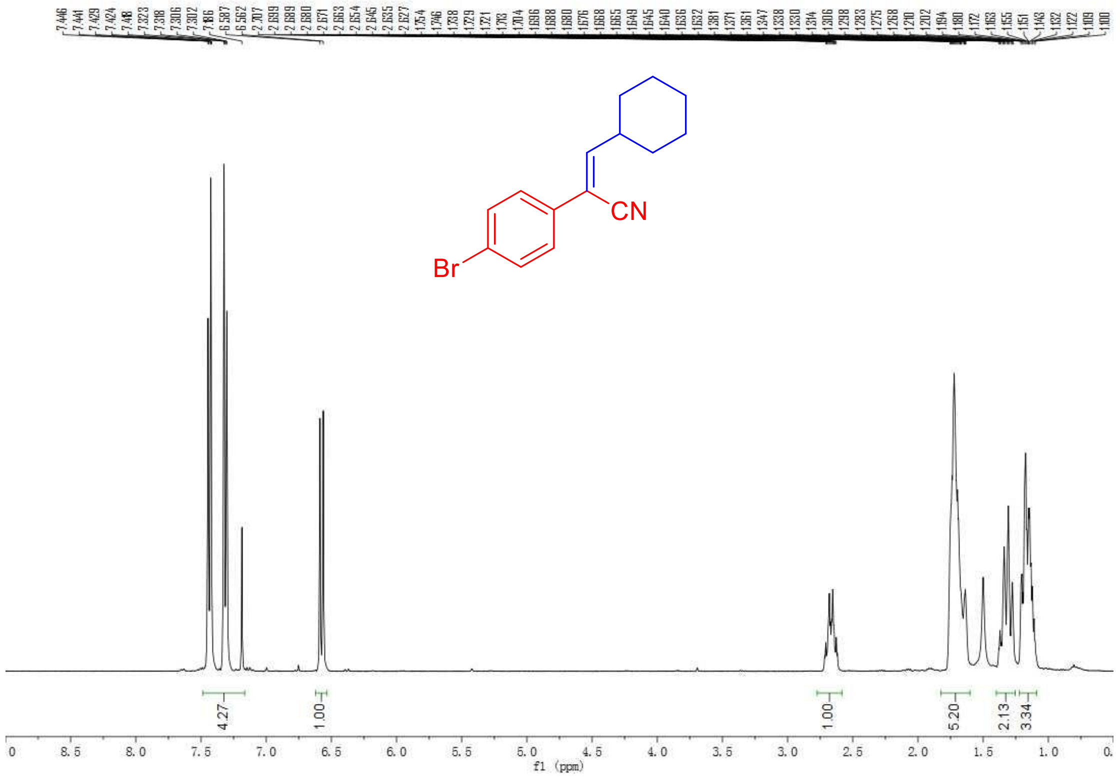

Figure S34. $^{13}C\{^{1}H\}$ **NMR spectra (100 MHz, Chloroform-*d*) of (*Z*)-2-(4-bromophenyl)-3-cyclohexylacrylonitrile (3gd).**

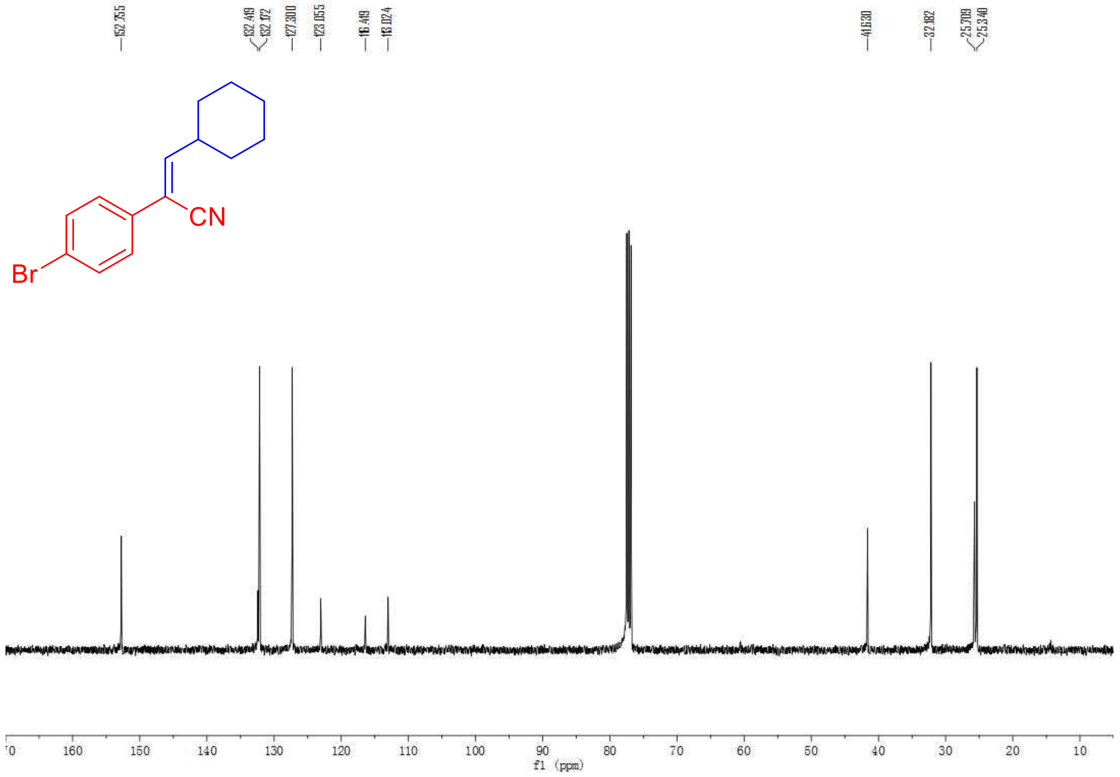

Figure S35. ^{1}H **NMR spectra (400 MHz, Chloroform-*d*) of (*Z*)-3-cyclohexyl-2-(naphthalen-2-yl)acrylonitrile (3hd).**

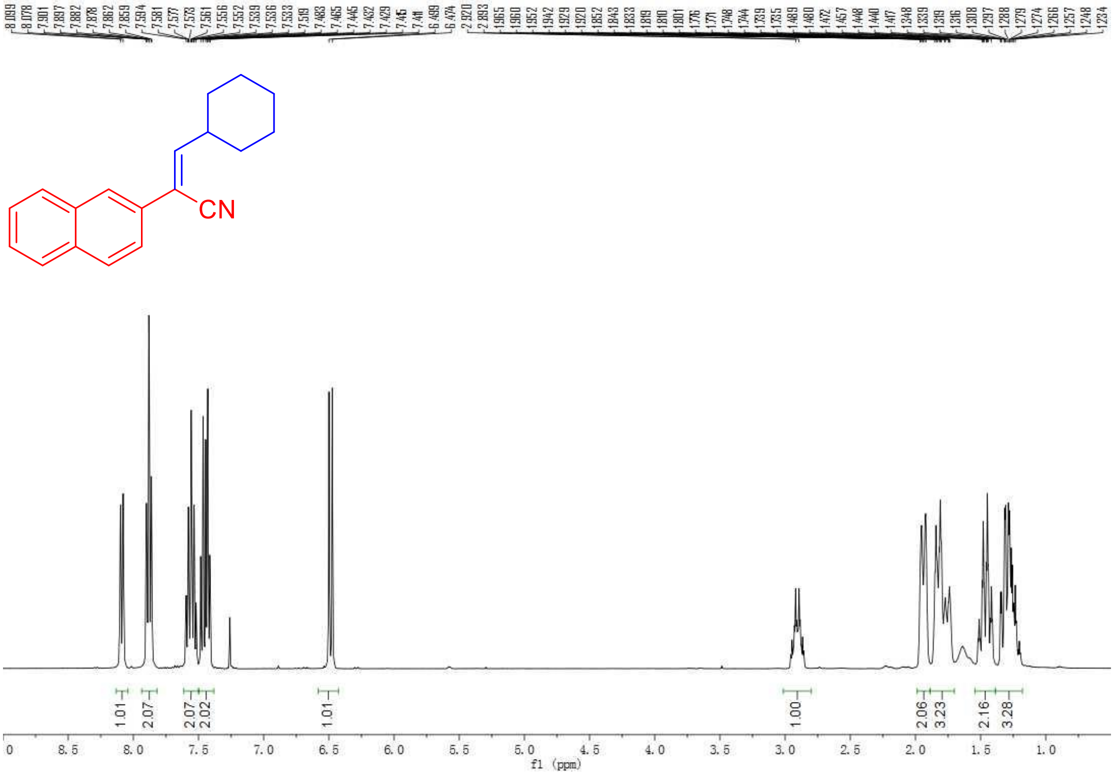

Figure S36. $^{13}C\{^{1}H\}$ **NMR spectra (100 MHz, Chloroform-*d*) of (*Z*)-3-cyclohexyl-2-(naphthalen-2-yl)acrylonitrile (3hd).**

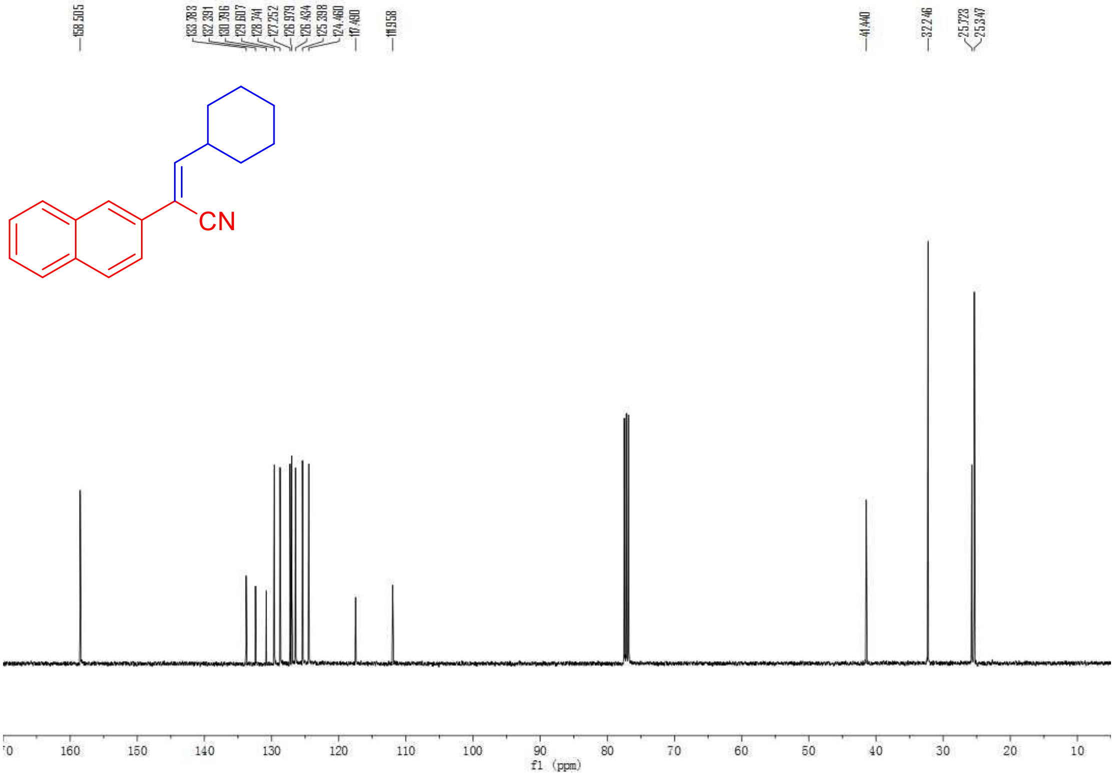

Figure S37. ^{1}**H NMR spectra (400 MHz, Chloroform-*d*) of (*Z*)-3-cyclohexyl-2-(1-methyl-1*H*-indol-3-yl)acrylonitrile (3id).**

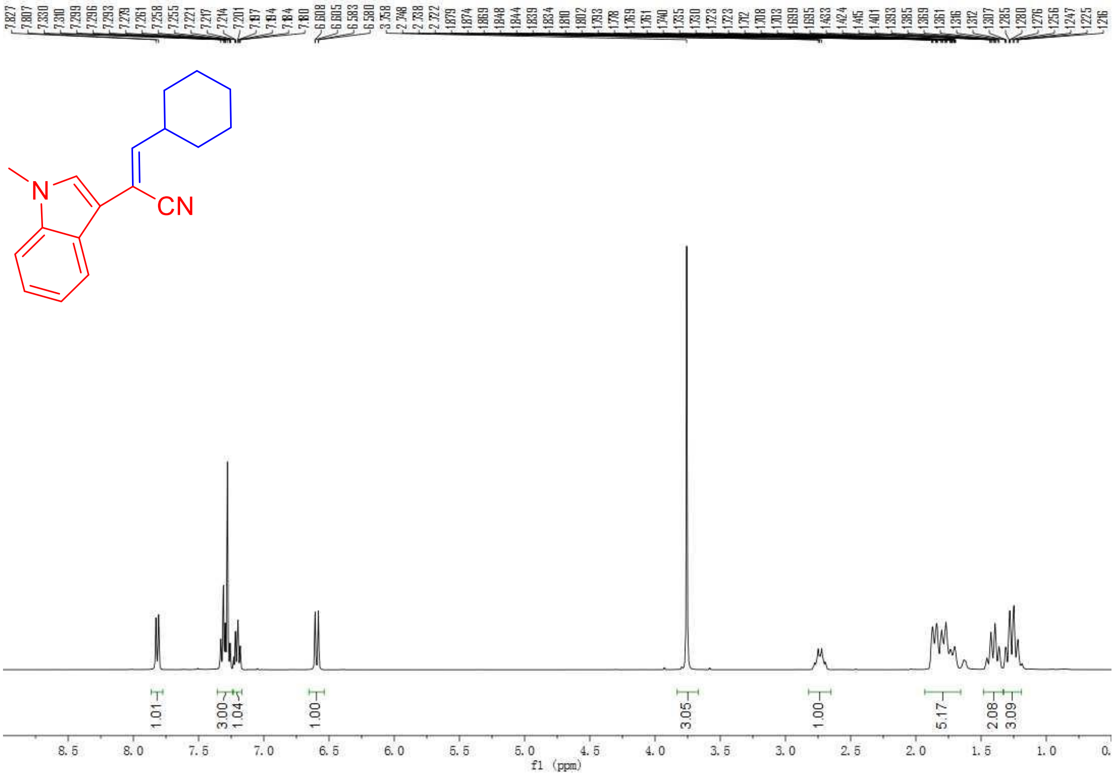

Figure S38. ^{13}**C{**^{1}**H} NMR spectra (100 MHz, Chloroform-*d*) of (*Z*)-3-cyclohexyl-2-(1-methyl-1*H*-indol-3-yl)acrylonitrile (3id).**

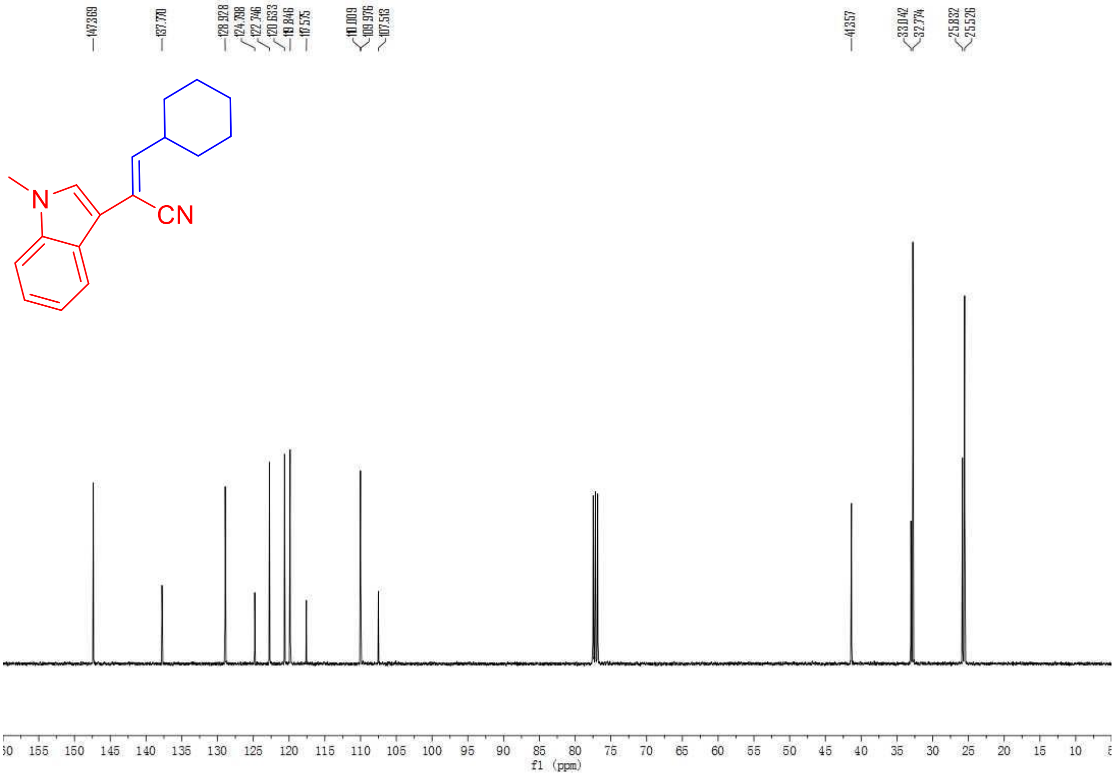

Figure S39. ^{1}H **NMR spectra (400 MHz, Chloroform-*d*) of (*E*)-3-cyclohexyl-2-(thiophen-2-yl)acrylonitrile (3jd).**

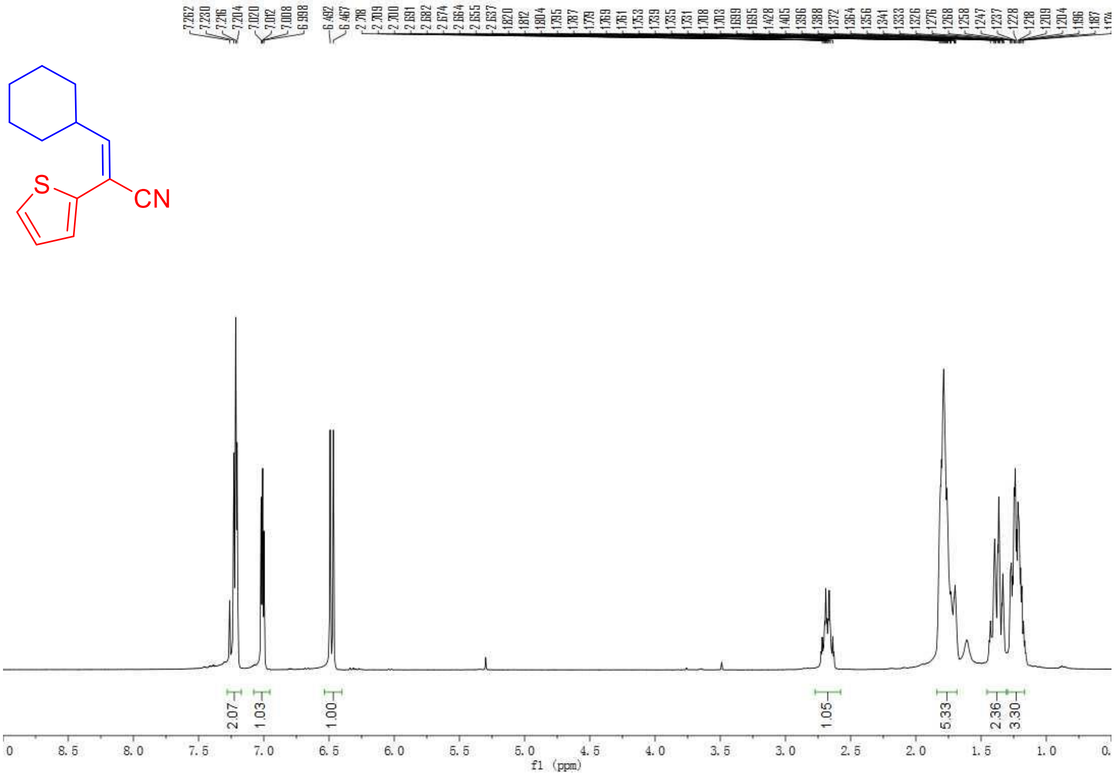

Figure S40. $^{13}C\{^{1}H\}$ **NMR spectra (100 MHz, Chloroform-*d*) of (*E*)-3-cyclohexyl-2-(thiophen-2-yl)acrylonitrile (3jd).**

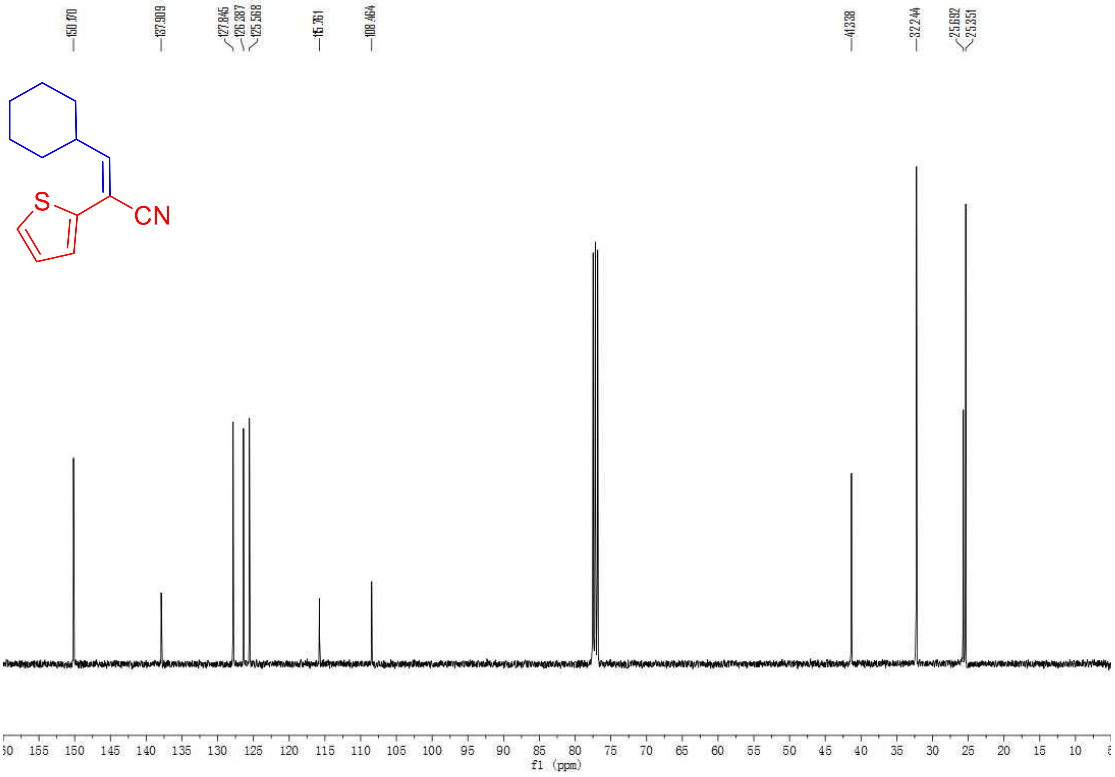

Figure S41. ^{1}**H NMR spectra (400 MHz, Chloroform-*d*) of 3-methyl-2-phenylbutanenitrile (4aa).**

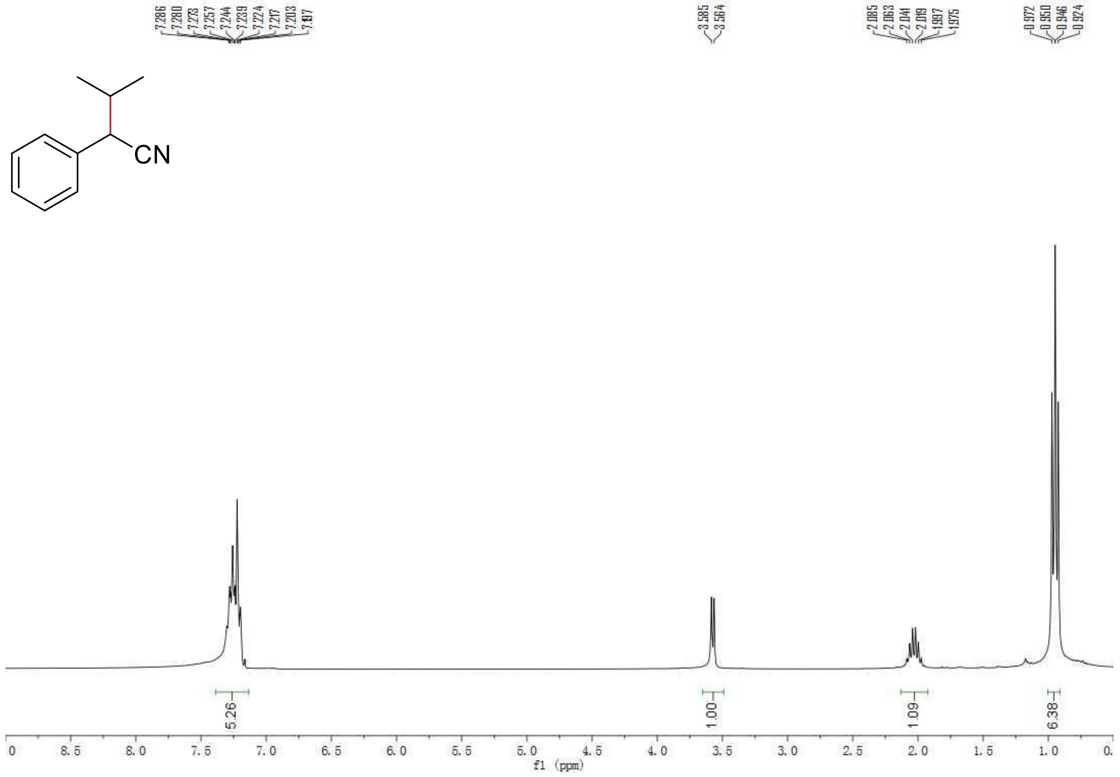

Figure S42. ^{13}**C{**^{1}**H} NMR spectra (100 MHz, Chloroform-*d*) of 3-methyl-2-phenylbutanenitrile (4aa).**

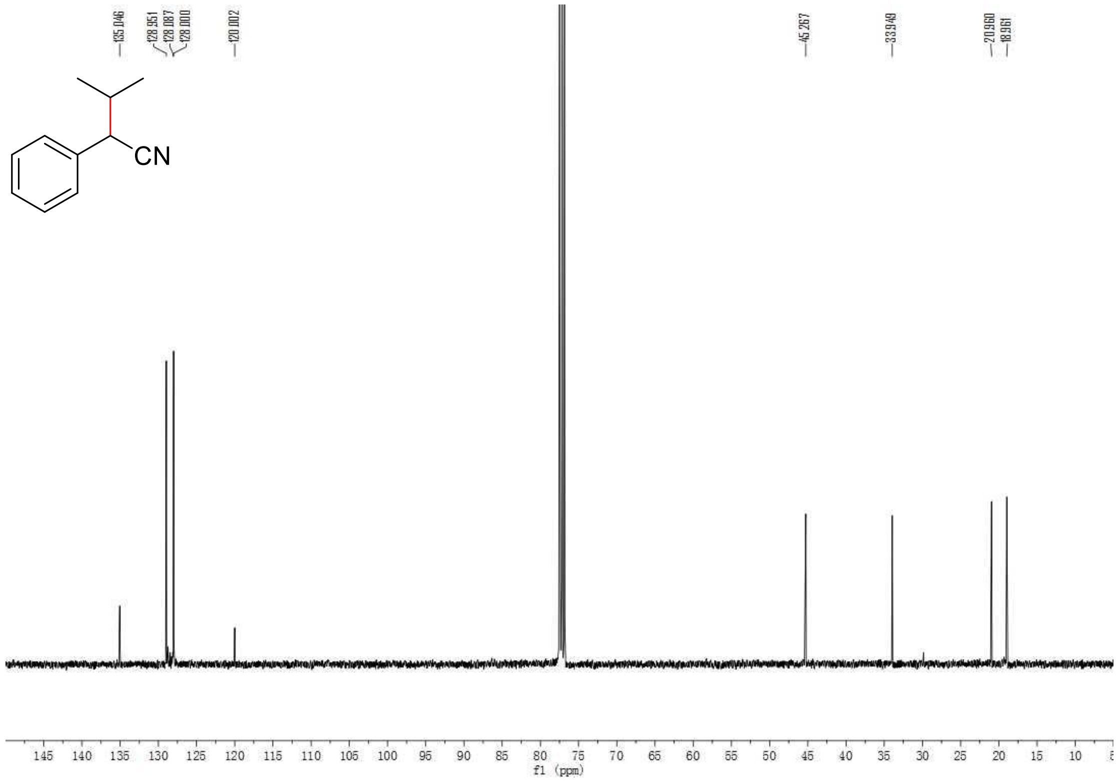

Figure S43. ^{1}H **NMR spectra (400 MHz, Chloroform-*d*) of 3-methyl-2-phenylbut-2-enal (4ab).**

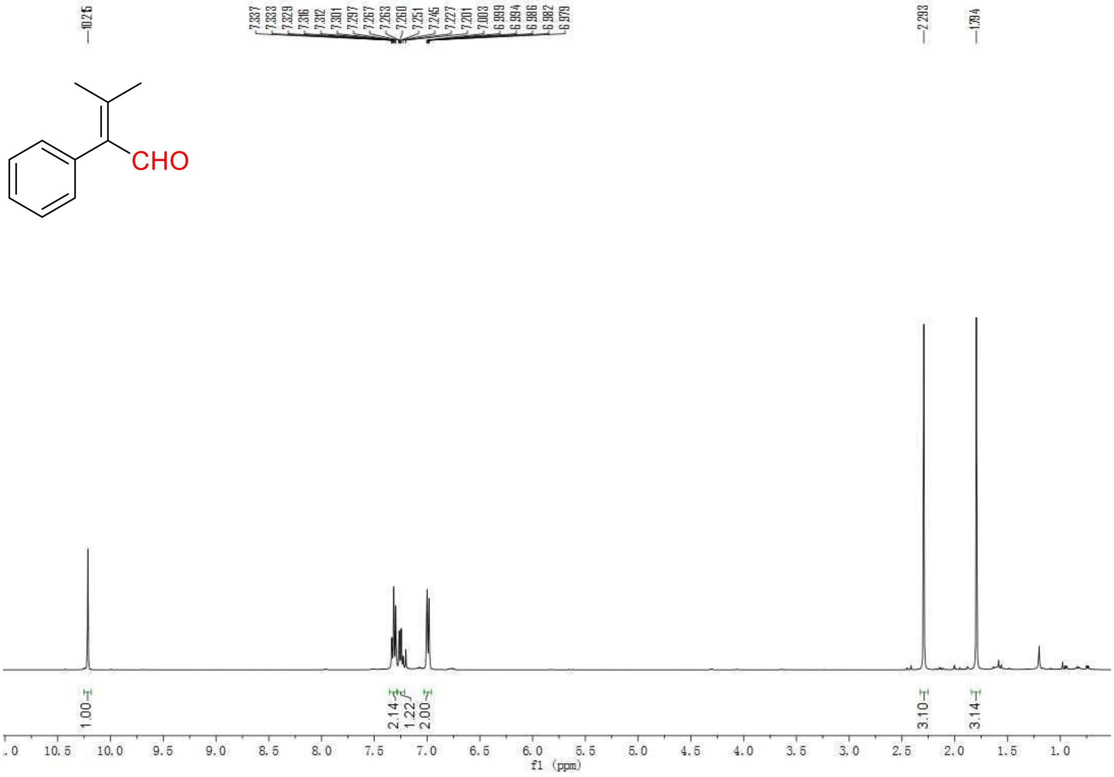

Figure S44. ^{13}C{^{1}H} **NMR spectra (100 MHz, Chloroform-*d*) of 3-methyl-2-phenylbut-2-enal (4ab).**

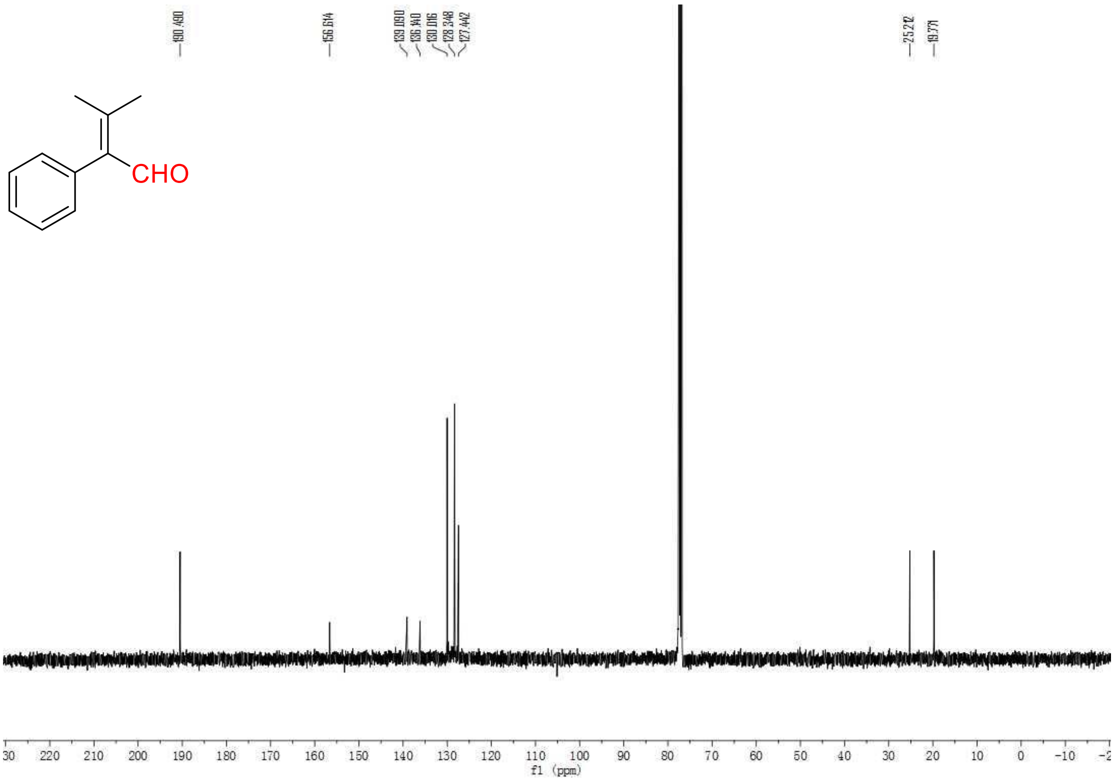

Figure S45. ^{1}H NMR spectra (400 MHz, Chloroform-*d*) of 3-methyl-2-phenylbut-2-enamide (4ac).

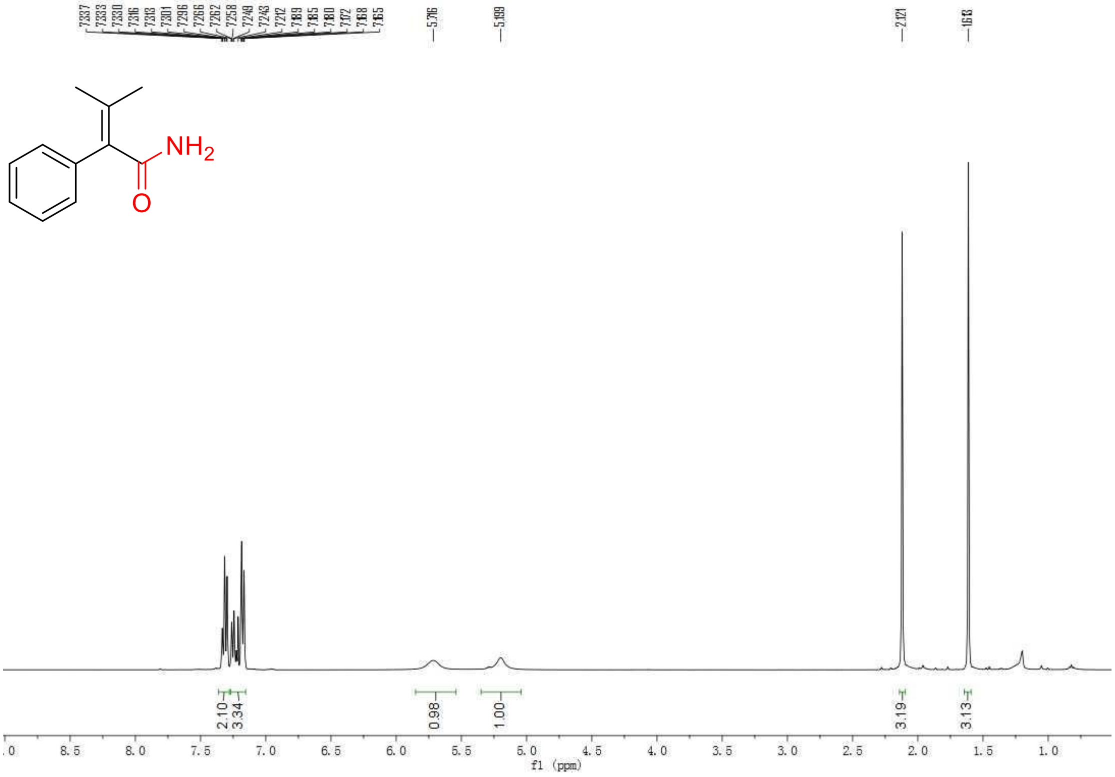

Figure S46. ^{13}C{^{1}H} NMR spectra (100 MHz, Chloroform-*d*) of 3-methyl-2-phenylbut-2-enamide (4ac).

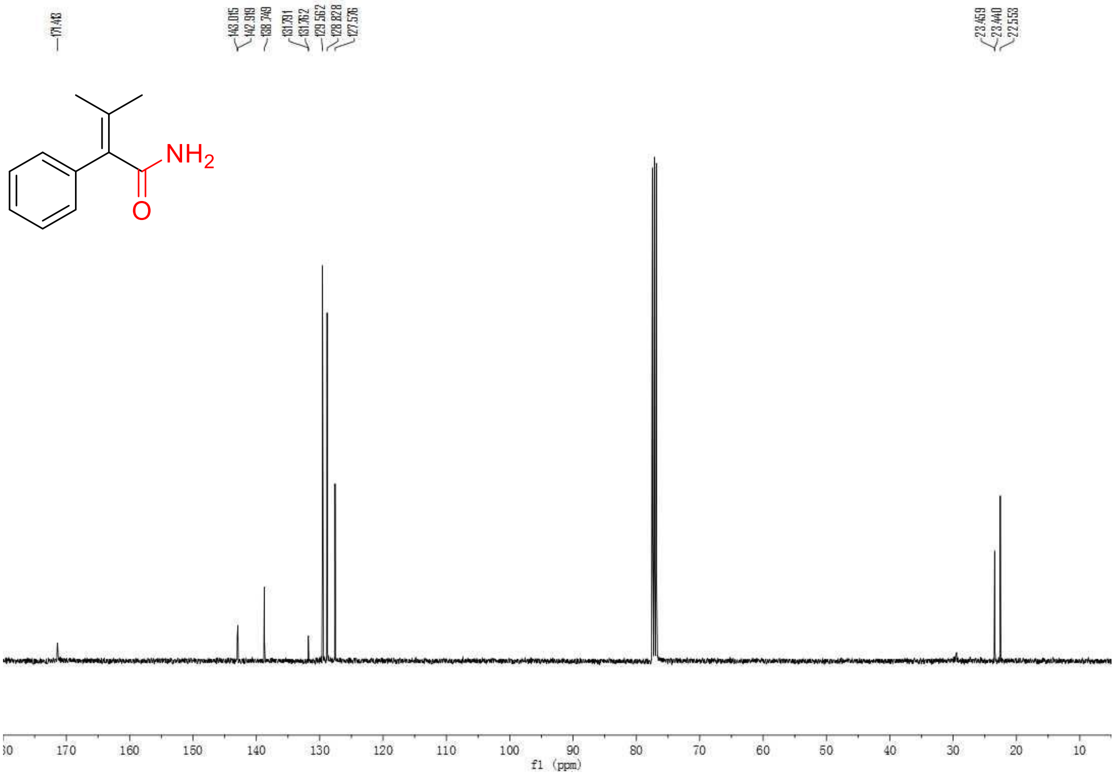

Figure S47. ^{1}H NMR spectra (400 MHz, Chloroform-*d*) of 3,3-dimethyl-2-phenyloxirane-2-carboxamide (4ad).

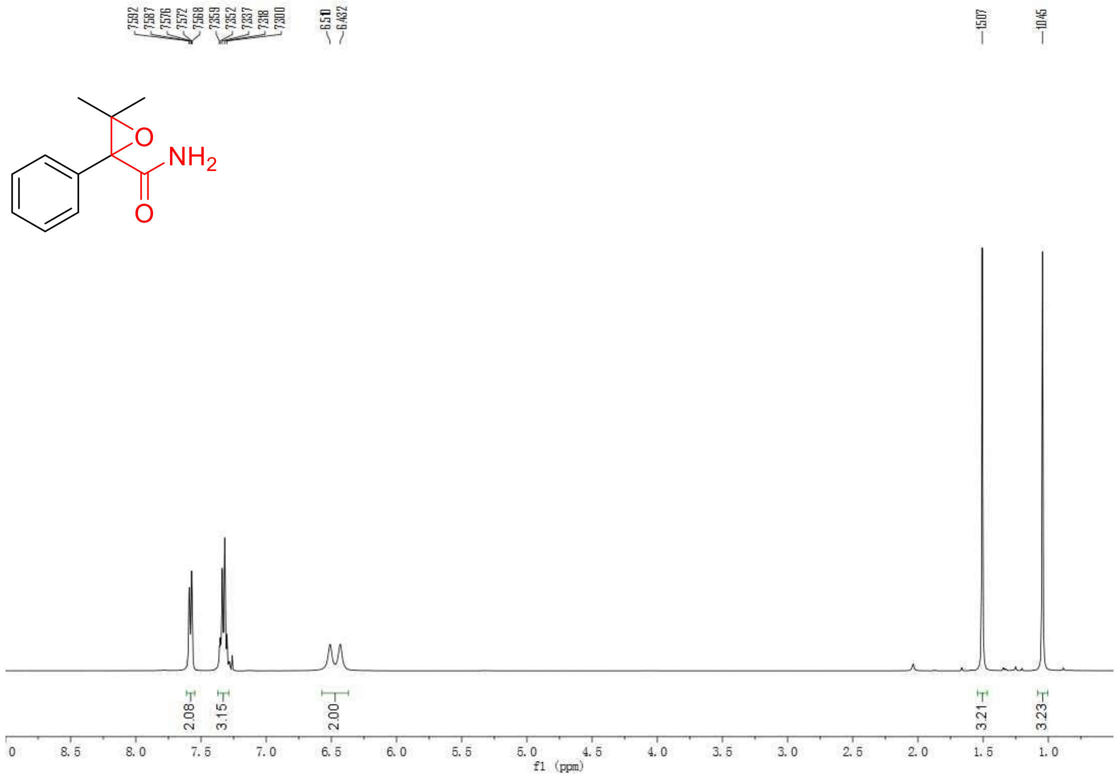

Figure S48. $^{13}C\{^{1}H\}$ NMR spectra (100 MHz, Chloroform-*d*) of 3,3-dimethyl-2-phenyloxirane-2-carboxamide (4ad).

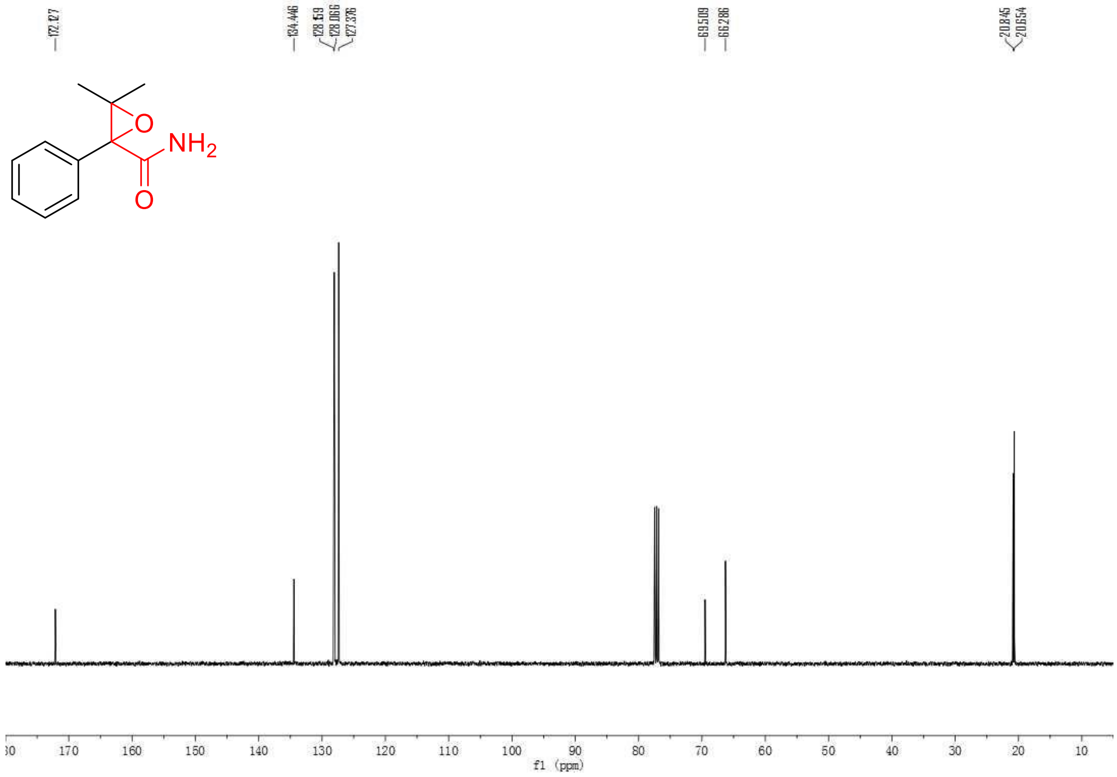

Supplement: Supplementary file 1 [file DataSheet1.pdf]
